# Circadian regulation of hedonic appetite in mice by clocks in dopaminergic neurons of the VTA

**DOI:** 10.1038/s41467-020-16882-6

**Published:** 2020-06-17

**Authors:** C. E. Koch, K. Begemann, J. T. Kiehn, L. Griewahn, J. Mauer, M. E. Hess, A. Moser, S. M. Schmid, J. C. Brüning, H. Oster

**Affiliations:** 1 Institute of Neurobiology, University of Lübeck, CBBM, Marie Curie Street, 23562 Lübeck, Germany; 2 Department of Neuronal Control of Metabolism, Max Planck Institute for Metabolism Research, Gleueler Street 50, 50931 Cologne, Germany; 3 Department of Neurology, University of Lübeck, CBBM, Marie Curie Street, 23562 Lübeck, Germany; 4 Institute of Endocrinology and Diabetes, University of Lübeck, CBBM, Marie Curie Street, 23562 Lübeck, Germany; 5 Deutsches Zentrum für Diabetesforschung e. V. (DZD), Neuherberg, Deutschland

**Keywords:** Circadian regulation, Feeding behaviour

## Abstract

Unlimited access to calorie-dense, palatable food is a hallmark of Western societies and substantially contributes to the worldwide rise of metabolic disorders. In addition to promoting overconsumption, palatable diets dampen daily intake patterns, further augmenting metabolic disruption. We developed a paradigm to reveal differential timing in the regulation of food intake behavior in mice. While homeostatic intake peaks in the active phase, conditioned place preference and choice experiments show an increased sensitivity to overeating on palatable food during the rest phase. This hedonic appetite rhythm is driven by endogenous circadian clocks in dopaminergic neurons of the ventral tegmental area (VTA). Mice with disrupted clock function in the VTA lose their hedonic overconsumption rhythms without affecting homeostatic intake. These findings assign a functional role of VTA clocks in modulating palatable feeding behaviors and identify a potential therapeutic route to counteract hyperphagy in an obesogenic environment.

## Abstract (web summary)

In addition to promoting overconsumption, palatable diets dampen daily intake patterns, which further augments metabolic dysfunction. Here, the authors find that in mice, circadian clocks in dopaminergic neurons in the ventral tegmental area drive hedonic appetite rhythms.

## Introduction

Modern societies are exposed to an obesity-promoting environment, with virtually unlimited access to and choice of different palatable, high-caloric foods and beverages. This environment facilitates a behavioral trend toward a dissociation of caloric intake from energy demands, promoting obesity and related metabolic disorders^1,2^. Besides increasing the risk of caloric overconsumption, ad libitum access to energy-dense food disrupts the regulation of endogenous circadian clocks^3^ evolved to anticipate and adapt to daily recurring environmental changes induced by the rotation of the earth around its axis. When food intake and environmental light conditions are aligned, a master circadian pacemaker located in the suprachiasmatic nucleus (SCN) synchronizes subordinated cellular clocks throughout the body to the environmental light/dark cycle^4^. Shifts in meal timing, however, can reset clocks of metabolically active tissues and uncouple them from the light-controlled SCN^5,6^. Accordingly, food intake during the rest phase leads to a state of internal circadian desynchrony promoting obesity, diabetes mellitus type 2 and other metabolic disorders in rodents^7–9^ and humans^10–13^. Thus, consumption of energy-dense food impairs metabolic control in a two-fold way, promoting obesity and its comorbidities in mammals^3,14^. The underlying mechanisms of how circadian disruption promotes metabolic abnormalities, however, are still poorly understood. Nuclei of the mediobasal hypothalamus (MBH) receive peripheral information about the energy state of the body to adjust food intake to homeostatic energetic requirements^2^. In addition, central reward circuits, especially dopaminergic projections from the ventral tegmental area (VTA) to the nucleus accumbens (NAc), affect the motivation to eat independent of homeostatic requirements^15–18^. While MBH clocks have been implicated in the regulation of rhythmic homeostatic intake behavior, it is less clear how hedonic aspects of appetite are controlled across the day^19–21^. There is accumulating evidence that the VTA is a key structure in the regulation of hedonic behaviors. Intake of energy-dense, palatable food activates VTA/NAc connections promoting dopamine release^18,22,23^ while low dopamine levels suppress the motivation to eat^24,25^. Further, optogenetic activation of VTA/NAc connections stimulates food reward-related behavior^24,26^. While it is still a matter of debate whether the VTA harbors a functional circadian clock, basal neuronal activity and gene expression patterns in this area show daily oscillations^27–35^. Since food intake is regulated via complex neuronal circuits involving several brain areas^2,15^, we hypothesize that homeostatic and hedonic aspects of appetite are regulated by spatially discrete CNS clocks, resulting in diet-dependent daily food intake patterns.

Using place preference and diet choice experiments, we aim at distinguishing homeostatic from hedonic aspects of circadian appetite regulation. Surprisingly, our experiments reveal a circadian vulnerability to palatable foods peaking in the rest phase, roughly anti-phasic to homeostatic intake rhythms. This hedonic appetite rhythm is controlled by clocks located in dopaminergic neurons of the VTA.

## Results

### Anti-phasic chow- and snack-induced conditioned place preference

**Fig. 1:**
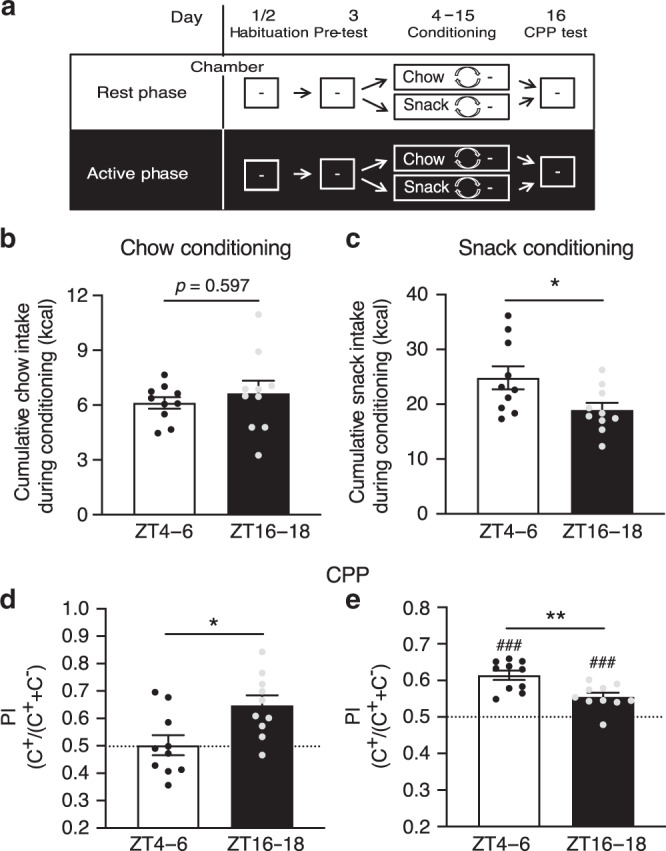
Time-of-day-dependent food conditioned place preference depends on the hedonic value. **a** Timeline and experimental design of the conditioned place preference (CPP) experiment. **b**, **c** Cumulative chow (**b**) and snack (**c**) intake during the conditioning sessions (C^+^) on days 4, 6, 8, 10, 12, and 14 at ZT6 or ZT18. **d**, **e** Calculated preference index (PI) of either chow (**d**) or snack (**e**) induced place preference assessed on day 16 (test day) at ZT6 or ZT18. C^+^ = conditioning with snack or chow; C^−^ = conditioning in an empty chamber; dashed line denotes chance level (PI = 0.5). Data are shown as mean ± SEM; *n* = 10; unpaired two-tailed *t*-test, ZT6 vs. ZT18, *p < 0.05; **p < 0.01 (**c**: *p* = 0.0285; **d**: *p* = 0.0107; **e**: *p* = 0.0027); one-sample two-tailed *t*-test against chance level (0.5), ^###^*p* < 0.001 (ZT4–6: *p* < 0.0001; ZT16–18: *p* = 0.007). All replicates are biologically independent samples. Source data are provided as a Source data file.

By modifying an unbiased conditioned place preference (CPP) paradigm, a routine method to assess the reinforcing efficacy of addictive substances, we analyzed time-of-day effects on the addictive capacity of bland (chow) and palatable (cookie) food (Fig. 1a). Homeostatic and hedonic aspects of food wanting were determined by measuring chow or hedonic snack intake during the conditioning sessions and the stimulus-induced place preference at the end of the experiment (day 16). Interestingly, while chow intake during the conditioning sessions was slightly, but not significantly, elevated during the dark phase (Fig. 1b), snack intake during conditioning was 25% higher during the light phase (Zeitgeber time (ZT) 4–6, in the normal rest phase of mice) compared with the dark/active phase (ZT16–18; Fig. 1c). Chow-induced place preference was only observed after conditioning in the active phase (preference index (PI) > 0.5; Fig. 1d). Snack-conditioned mice, however, showed place preference after conditioning at both time points (Fig. 1e). Moreover, snack-induced place preference was significantly stronger after conditioning during the rest phase (ZT4–6; Fig. 1e). Such anti-phasic reinforcing capacity of palatable (hedonic) and bland (homeostatic) foods suggests the involvement of distinct circadian clocks. However, CPP heavily depends on spatial learning, memory consolidation and the recall of information, all of which are under circadian control^16^ and may thereby influence the experimental outcome.

### Anti-phasic homeostatic and hedonic appetite rhythms

**Fig. 2:**
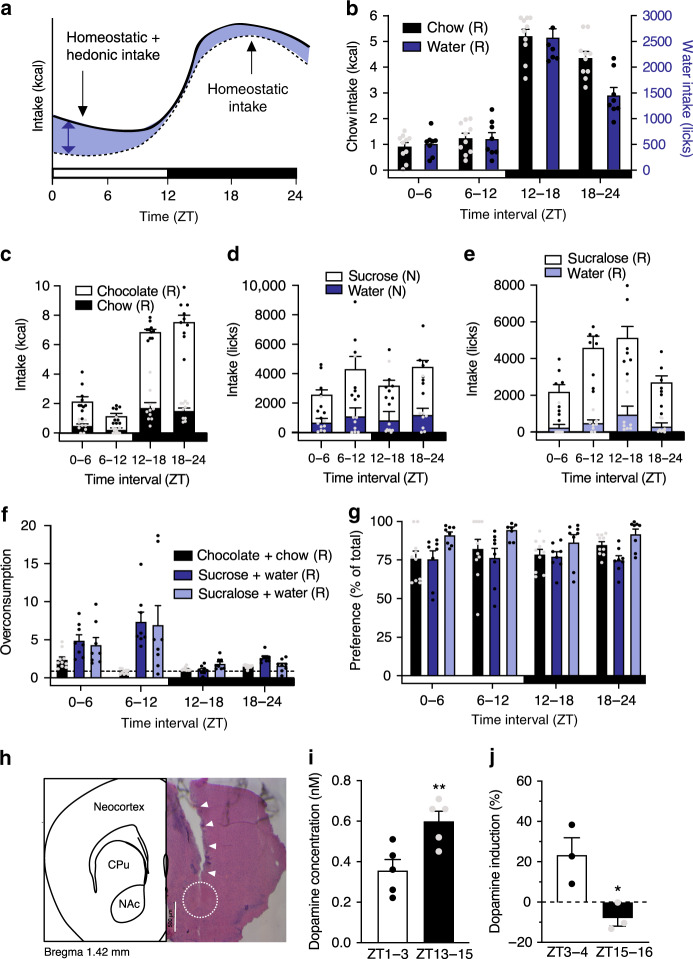
Anti-phasic oscillations of homeostatic and hedonic overconsumption rhythms under light/dark conditions. **a** Schematic representation depicting homeostatic intake (chow-only; black dotted line), hedonic plus homeostatic intake (black line) and hedonic choice-induced overconsumption (blue area). **b** Nighttime-increased homeostatic chow (black) and water intake (blue) in WT mice. **c**–**g** Diurnal intake rhythms in hedonic/homeostatic choice experiments with **c** chow and chocolate (black/white), **d** sucrose solution and water (blue), or **e** sucralose solution and water (light blue) choices. **f** Elevated overconsumption (relative to chow/water-only intake) in hedonic/homeostatic choice experiments during the normal rest phase. **d** Time-of-day-dependent preference for chocolate, sucrose, and sucralose during the choice experiments. Data are shown as mean ± SEM; *n* = 10 (chow/chocolate) or 8 (water/sucrose or sucralose). White box = light phase, black box = dark phase; dashed line depicts baseline chow/water intake. Letters in legend depict statistically significant (R) or non-significant (N) 24-h rhythmicity (CircWave; *p* < 0.05). **h** Representative micrograph of H&E stain depicting the position of microdialysis probes with an overview of brain regions. White bar indicates 500 µm; triangles indicate probe canal; circle indicates dialysis area; CPu—caudate putamen. Probe location was verified for all animals (*n* = 10); no animal was excluded. **i** Dopamine levels in NAc microdialysate at the depicted time points (*n* = 5) and **j** individual relative changes in NAc dopamine concentrations after a chocolate snack (*n* = 3). Data are shown as mean ± SEM; unpaired two-tailed *t*-test, *p < 0.05, **p < 0.01 (**i**: *p* = 0.0098; **j**: *p* = 0.0286). All replicates are biologically independent samples. Source data are provided as a Source data file.

To corroborate our findings in a spatial memory-independent paradigm we next measured food/liquid intake in circadian choice experiments. To asses hedonic appetite against the background of homeostatic energy demands, we calculated the overconsumption during chow/chocolate or water/hedonic liquid (sucrose/sucralose) choice conditions relative to bland food/liquid intake (homeostatic intake) under non-choice conditions (Fig. 2a).

Similar to the increased chow-induced place preference during the active phase (Fig. 1b), ad libitum chow and water consumption were elevated during the active phase peaking at ZT12–18 (Fig. 2b). Under choice conditions, this overall rhythm in energy intake was conserved, though chow and water intake were strongly reduced and replaced by chocolate or sucrose/sucralose solution (Fig. 2c–e). Relative to non-choice chow (i.e., homeostatic) intake hedonic overconsumption under choice conditions was 2–7-fold increased during the normal rest phase with strongest effects at ZT0–6 for chocolate and ZT6–12 for sucrose and sucralose (Fig. 2f). Likewise, cumulative 24-h caloric intake during the choice experiments was significantly increased compared with homeostatic (non-choice) conditions (Supplementary Fig. 1a). In contrast to overconsumption rates, the general preference for sweet taste (chocolate or sucrose/sucralose) over chow/water under choice conditions did not show circadian variation (Fig. 2g).

Dopamine is an important neurotransmitter of the central reward system that has previously been associated with food reward^36^. We therefore tested the diurnal effects of palatable food consumption on reward circuits by measuring dopamine release in the nucleus accumbens (NAc) as the main target of dopaminergic projections in the context of reward signaling^37^ by in vivo microdialysis. Mice were bilaterally implanted with microdialysis cannulae reaching into the NAc (Fig. 2h). Baseline dopamine levels were determined from dialysate during 2 h in the morning (ZT1–3) and the early night (ZT13–15). In line with previous publications^35^, baseline NAc dopamine levels were highly variable, but elevated during the night (Fig. 2i). When mice received chocolate, individual dopamine concentrations in the NAc were increased by about 35% in the morning, but levels were largely unresponsive during the night (Fig. 2j). Of note, during the snack time mice on average ate 0.46 g of chocolate (ca. 75% of the total amount offered) with no significant differences between time points (ZT1–3: 0.41 ± 0.08 g; ZT13–15: 0.51 ± 0.04; *p* = 0.308, unpaired *t*-test).

Together, CPP and food/drink choice experiments suggest a roughly anti-phasic circadian regulation of homeostatic and hedonic appetite. In line with an increased hedonic overconsumption during the rest phase under choice conditions chocolate induced dopamine responses in the NAc were elevated in the morning. Sucralose-induced overconsumption rhythms were largely comparable to those of sucrose, arguing that palatability—here: sweet taste—rather than mere caloric density drives this effect.

### Clock controlled anti-phasic intake patterns

**Fig. 3:**
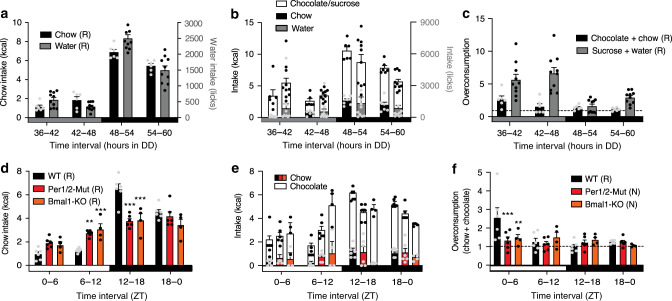
Circadian clock controlled anti-phasic oscillations of homeostatic and hedonic appetite rhythms. **a**–**c** Anti-phasic homeostatic chow or water intake (**a**) and hedonic (chow plus chocolate or sucrose plus water) overconsumption (**b**, **c**) rhythms persist in WT mice in constant darkness (DD). Gray box = subjective day/rest phase, black box = subjective night/active phase. **d**–**f** Homeostatic intake (**d**) and hedonic overconsumption (**e**, **f**) rhythms are significantly dampened in clock-deficient *Per1/2* double-mutant and *Bmal1*-KO mice. White box = light phase, black box = dark phase. Data are shown as mean ± SEM; WT *n* = 6 (chow) or 10 (water); *Per1/2* *n* = 6; *Bmal1*-KO *n* = 4; dashed lines depicting homeostatic chow/water intake. Two-way ANOVA with Sidak post hoc test against WT, *p < 0.05; **p < 0.01; ***p < 0.001 (**d**: Per1/2-Mut/ZT6–12 *p* = 0.0026; ZT12–18 *p* < 0.0001; Bmal1-KO/ZT6–12 *p* = 0.0009; ZT12–18 *p* < 0.0001; **f**: Per1/2-Mut/ZT0–6 *p* = 0.0006; Bmal1-KO/ZT0–6 *p* = 0.0062). Letters in brackets (R/N) indicate diurnal rhythmicity (*p* < 0.05; CircWave). All replicates are biologically independent samples. Source data are provided as a Source data file.

To test if these oscillations were controlled by the endogenous circadian clock system rather than external factors such as the light/dark cycle, we repeated the choice experiments under Zeitgeber-free conditions in constant darkness (DD) and with mice carrying a genetic ablation of circadian clock function. On the second day in DD, homeostatic intake remained elevated during the subjective active phase, with a peak at 48–54 h after lights off (Fig. 3a). Under these conditions, hedonic overconsumption was again higher during the subjective rest phase peaking at 36–42 h for chocolate and 42–48 h for sucrose, respectively (Fig. 3b, c). Choice-induced 24-h overconsumption was preserved in DD (Supplementary Fig. 1b). Chocolate as well as sucrose preference were, again, largely independent of circadian time (Fig. 3b).

In clock-deficient *Per1/2* double-mutant (*Per1/2*) and *Bmal1* knockout mice (*Bmal1*-KO), homeostatic appetite was elevated during the dark phase, but 24-h rhythms were dampened compared with wild-type (WT) controls (Fig. 3d, Supplementary Fig. 2a). This residual rhythm likely reflects light masking which is also seen for locomotor activity and is abolished immediately upon release into DD^38,39^ Under choice conditions, overconsumption rhythms were abolished and overall overconsumption reduced in both clock mutant mouse models (Fig. 3e, f, Supplementary Fig. 3b). Notably, only the rhythmicity of hedonic and homeostatic intake was affected; cumulative 24-h intake and sweet preference were largely comparable between WTs and clock gene mutants (Fig. 3e, Supplementary Fig. 1c). In summary, DD and clock mutant data indicate a regulation of, both, homeostatic and hedonic intake behavior by the endogenous circadian clock.

### VTA diurnal transcriptome rhythms

**Fig. 4:**
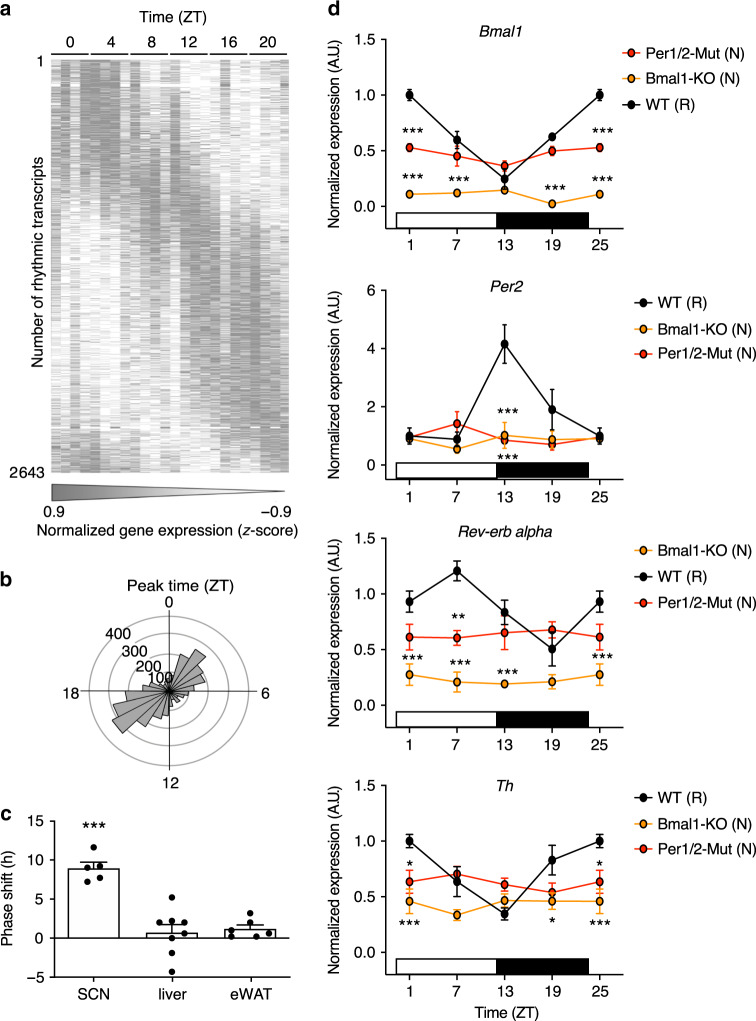
Diurnal transcriptome rhythms in the VTA. **a** Heat map of normalized and phase-sorted rhythmically expressed genes in the VTA of WT mice (*n* = 4 per time point). **b** Peak time distribution of rhythmically expressed genes in the VTA showing two clusters at the beginning of the light and the beginning of the dark phase. **c** Phase shifts of core clock gene expression in the VTA compared with SCN, liver, eWAT. Positive values indicate phase delays of the VTA clock (*n* = 5 (SCN); 6 (eWAT); 8 (liver)); one-sample two-tailed *t*-test against chance level (0), ***p < 0.00 (SCN: *p* < 0.0001). **d** VTA gene expression rhythms in WT, *Per1/2* double mutants (Per1/2-Mut), and *Bmal1* deficient (Bmal1-KO) mice for *Bmal1*, *Per2, Rev-erba*, and *Th mRNA*. Data are shown as mean ± SEM; *n*-sizes for WT: 4 (ZT13) or 3 (all other TPs); Per1/2-Mut: 3 (ZT7) or 4 (all other TPs); Bmal1-KO: 3 (ZT7 and 19) or 4 (all other TPs); two-way ANOVA with Sidak post hoc test vs. WT; *p < 0.05; **p < 0.01; ***p < 0.001 (*Bmal1* and *Per2*: all indicated *p*-values <0.0001; *Rev-erb alpha*: Per1/2-Mut ZT7 *p* = 0.0013, all other indicated *p*-values <0.0001; *Th*: Per1/2-Mut ZT1/25 *p* = 0.0119, Bmal1-KO ZT1/25 *p* < 0.0001, and ZT19 *p* = 0.0174). Letters in brackets indicate 24-h rhythmicity (*p* < 0.05; CircWave); white box = light phase, black box = dark phase. All replicates are biologically independent samples. Source data are provided as a Source data file.

While homeostatic food intake is mainly regulated via hypothalamic circuits, hedonic appetite rhythms are more likely to be controlled by the dopaminergic system originating in the VTA, due to its essential role in reward regulation and the motivation to eat^17–19^. Thus, we next characterized general diurnal regulation in this brain area. We analyzed mRNA oscillations from micro-punches over 24 h by whole-genome microarray hybridization under standard 12-h light/12-h dark (LD) conditions. Roughly 10% of all investigated transcripts (*n* = 2643) in the VTA showed a rhythmic expression pattern (Fig. 4a; Supplementary Data 1). Phase analysis of these transcript rhythms yielded two main clusters peaking around ZT3 and ZT16 (Fig. 4b). Phase comparison of canonical clock gene transcripts indicated a ~9-h delayed phase of the VTA clock compared with the SCN pacemaker (Fig. 4c), similar to what had previously been reported for other non-SCN clocks in the brain^20^. Peak time comparison between VTA and peripheral tissue clock gene expression in liver and epididymal white adipose tissue (eWAT) yielded a phase difference of ca. 1 h (Fig. 4c).

In WT mice, investigated clock genes showed a canonical phase distribution pattern with *Bmal1* expression peaking at the beginning of the rest and *Rev-erb alpha* and *Per2* at the end of the rest and the beginning of the active phase, respectively (Fig. 4d). In line with the dampened homeostatic and hedonic intake rhythms of clock-deficient *Per1/2* double-mutant and *Bmal1* knockout mice (Fig. 3), VTA clock gene expression rhythms were blunted in these animals (Fig. 4d). So far, these data were in line with our hypothesis that the VTA harbors a functional circadian clock, which might be involved in rhythmic appetite regulation.

Further support was provided by gene set enrichment analysis of rhythmic VTA transcripts, yielding high numbers of rhythmic mRNAs associated with metabolic processes (*n* = 496), response to stimuli (*n* = 376) as well as cell communication (*n* = 250) hinting to circadian clock-controlled neuronal communication routes. Moreover, VTA mRNA expression of the rate-limiting enzyme of dopamine biosynthesis, tyrosine hydroxylase (*Th*), was rhythmically regulated in WT, but not in clock-deficient mutant mice (Fig. 4d). Interestingly, maximal *Th* expression in WT mice was phase-aligned with elevated chocolate intake at the beginning of the rest phase. Moreover, reduced *Th* expression in the VTA of *Per1/2* mutants and *Bmal1* knockouts at this time was associated with blunted hedonic overconsumption (Figs. 3f, 4d).

Together, these analyses suggest the presence of a functional circadian clock in VTA neurons, coordinating rhythmic transcriptome regulation in this brain region. *Th* expression in the VTA was rhythmic in WT, but not in clock mutant mice. Similarly, hedonic overconsumption was dependent on clock function. Together, this makes the VTA clock a promising candidate for the diurnal regulation of hedonically motivated food intake.

### VTA clocks regulate hedonic overconsumption rhythms

**Fig. 5:**
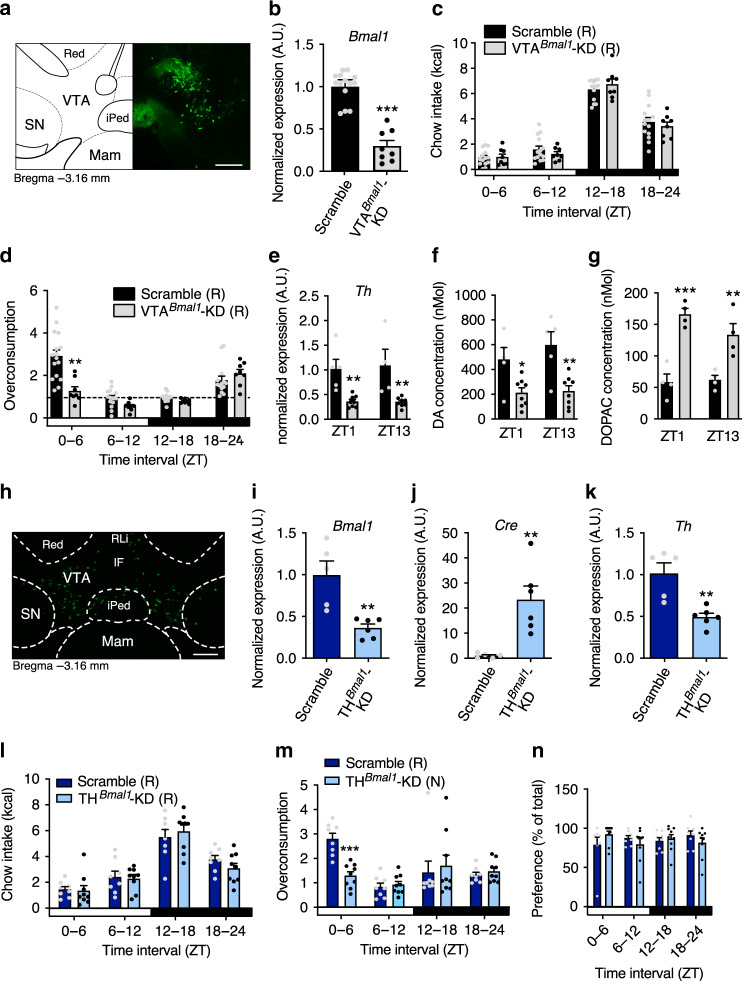
Hedonic appetite rhythms are regulated by circadian clocks in dopaminergic neurons of the VTA. **a** GFP fluorescence after stereotaxic GFP AAV injection into the VTA (white bar = 200 µm). **b** *Bmal1* gene expression at ZT1 seven weeks after AAV-mediated knockdown of *Bmal1* in the VTA (VTA^*Bmal1*^-KD; *n* = 8) and control mice (with scramble AAV injections; *n* = 14). **c**, **d** Chow intake (**c**) and hedonic overconsumption (**d**) of VTA^*Bmal1*^-KD and control mice (*n* = 14 for scramble AAV injections; *n* = 8 for VTA^*Bmal1*^-KD). **e** *Th* gene expression in the VTA of VTA^*Bmal1*^-KD and control mice. **f**, **g** Dopamine (**f**) and DOPAC (**g**) concentrations in the NAc of VTA^*Bmal1*^-KD and control mice. **h** zsGreen fluorescence after stereotaxic TH-Cre AAV injection into the VTA of zsGreen reporter mice (white bar = 200 µm). **i**–**k** mRNA expression at ZT1 of *Bmal1* (**i**), *Cre* (**j**), and *Th* (**k**) in the VTA of TH^*Bmal1*^-KD (*n* = 6) and control (scramble AAV-injected; *n* = 5) mice. **l**–**n** Chow intake (**l**), hedonic overconsumption (**m**), and chocolate preference (**n**) of TH^*Bmal1*^-KD (*n* = 9) and control mice (*n* = 8). Data are shown as mean ± SEM; unpaired two-sided *t*-test (**b**, **i**–**k**) or two-way ANOVA with Sidak post hoc test (**d**–**g**, **l**–**n**); **p < 0.01; ***p < 0.001. **b**: *p* < 0.0001; **d**: ZT0–6 *p* = 0.012; **e**: ZT1 *p* = 0.0037, ZT13 *p* = 0.0023; **f**: ZT1 *p* = 0.0199, ZT13 *p* = 0.0015; **g**: ZT1 *p* = 0.0001, ZT13 *p* = 0.0034; **i**: *p* = 0.0036; **j**: *p* = 0.0049; **k**: *p* = 0.0025; **m**: ZT0–6 *p* = 0.0006. White box = light phase, black box = dark phase; dashed lines depicting baseline chow intake. Red red nucleus, RLi rostral linear nucleus raphe, iPed interpeduncular nucleus, SN substantia nigra, Mam mammillary nucleus, IF interfascicular nucleus. All replicates are biologically independent samples. Source data are provided as a Source data file.

To test whether the VTA clock is involved in homeostatic or hedonic appetite regulation, we characterized feeding behavior in mice with genetically inhibited circadian clock function specifically in the VTA. Stereotaxic injections of GFP/CRE recombinase-expressing adeno-associated virus in *Bmal1*^*flx/flx*^ mice were used to knockdown clock function in the VTA (VTA^Bmal1^-KD) with injections of AAVs expressing a scrambled cDNA sequence as controls. Targeting of virus injections was confirmed by analyzing GFP fluorescence on brain sections (Fig. 5a). Expression analyses from laser capture microdissections showed an AAV-mediated *Bmal1* KD of ca. 70% specifically in the VTA (Fig. 5b, Supplementary Fig. 3a, b). VTA^Bmal1^-KD mice showed normal homeostatic food intake rhythms (Fig. 5c). Diurnal hedonic overconsumption rhythms, however, were dampened and overall overconsumption reduced in VTA^Bmal1^-KD mice (Fig. 5d, Supplementary Fig. 3c). *TH* mRNA levels in the VTA of VTA^Bmal1^-KD mice were overall reduced (Fig. 5e), similar to those in *Bmal1*-KO mice (Fig. 4d), and associated with reduced NAc dopamine and increased 3,4-dihydroxyphenylacetic acid (DOPAC) concentrations as measured from micro-punches (Fig. 5f, g). Together, these findings suggest a reward-related impact of clocks in the VTA on diurnal food choice regulation and time-of-day-dependent changes in DA signaling in the NAc.

To further test this association, we manipulated *Bmal1* specifically in dopaminergic neurons of the VTA by bilateral stereotaxic injections of an AAV expressing CRE recombinase under control of the *TH* promotor, thus restricting CRE-mediated recombination to dopaminergic neurons (TH^*Bmal1*^-KD). Recombination was validated by injecting TH-CRE AAV into zsGreen reporter mice and analysis of zsGreen fluorescence on sections (Fig. 5h). *Bmal1* knockdown, *Cre* expression and suppression of *Th* mRNA in the VTA were confirmed by qPCR on laser capture microdissections (Fig. 5i–k, Supplementary Fig. 4a). Only mice with more than 50% reduction in VTA *Bmal1* mRNA levels were included in the analysis. Similar to VTA^*Bmal1*^-KD mice, *Bmal1* knockdown in dopaminergic neurons did not affect homeostatic chow intake, but blunted hedonic overconsumption rhythms (Fig. 5l, m, Supplementary Fig. 4b). Rest phase overconsumption was reduced by ca. 50% and largely comparable with the homeostatic intake at this time-of-day. Chocolate preference, again, was unaffected by time-of-day and consistently high (Fig. 5n, Supplementary Fig. 4b).

In summary, our findings assign a functional role to dopaminergic VTA circadian clocks in the regulation of hedonic overconsumption, but not homeostatic energy intake regulation across the day. Increased vulnerability to palatable food during the normal rest phase coincided with increased *Th* expression in the VTA and dopamine responses in the NAc. The importance of a functional clock in dopaminergic neurons for hedonic appetite was further supported by abolished hedonic overconsumption rhythms in mice without functional clocks in dopaminergic neurons of the VTA.

## Discussion

The increasing prevalence of obesity has been recognized by the World Health Organization (WHO) as one of today’s principal challenges of the health system. Recent studies in rodents and humans suggest that not only the amount, but also the timing of caloric intake plays an important role for the obesogenic properties of food, indicating a role of the circadian clock in the regulation of appetite and energy homeostasis^7,13^. To understand the interaction of different tissue circadian clocks with homeostatic and hedonic aspects of appetite regulation, we analyzed the diurnal control of bland and palatable food intake in mice with global and specific ablation of clock function in neurons of the central reward system. Our data suggest that clocks residing in dopaminergic neurons of the VTA regulate hedonic aspects of circadian appetite regulation, thus determining the vulnerability to palatable snacks across the day.

To define the chrono-disruptive effects of energy-dense diets, it is important to discriminate between different aspects of appetite regulation by disentangling homeostatic from hedonic circadian appetite rhythms^25^. Lever pressing or CPP experiments are often used to quantify the effects of different stimuli or genotypes at one time point, but do not consider circadian variations. We therefore modified the standard CPP paradigm to allow for consideration of time-of-day effects on homeostatically and hedonically motivated intake behaviors across the day. By comparing chow- with hedonic snack-induced place preference we revealed an anti-phasic regulation with increased homeostasis-driven behavior during the active phase and elevated hedonically motivated intake behavior during the rest phase. The interpretability of circadian experiments investigating CPP effects during the course of the day may be confounded by circadian clock-mediated learning and memory effects^16^ since rodents have to learn and recall a specific task during several training/conditioning sessions before behavior can be quantified on the test day (e.g., lever pressing^26^). Therefore, we employed a less learning-dependent ab libitum approach to investigate the different aspects of appetite regulation. Typical high-fat diet (HFD) or choice studies measure the net intake of HFD or choice diets over the course of the day, the outcome of which is often interpreted as rhythm of the reward system. However, both, HFD and choice intake^27^ are a combination of homeostatic demand-controlled and hedonically motivated intake and do not reflect hedonically motivated intake alone.

We therefore used a combination of hedonic/homeostatic choice experiments in an attempt to better distinguish hedonism- from homeostasis-driven intake behavior. By normalizing the caloric intake during the choice experiment (snack plus chow) to the homeostatic (non-choice) chow intake, i.e., calculating the capacity of the palatable snack to induce hyperphagy, or overeating, we were able to dissect both aspects of food intake. Our findings confirmed the CPP results, showing that circadian regulation of homeostatic and hedonic appetite show distinct temporal profiles with roughly anti-phasic rhythmicity. Of note, overconsumption rhythms in sweet drink intake appear slightly phase delayed as compared with palatable food (compare peaks in Fig. 2f). This may reflect differences in homeostatic rhythms (as indicated in Fig. 2b) or masking of the exact peak time by the coarse sampling interval of 6 h, but it may also point at distinct circuits transmitting VTA clock-controlled outputs to food appetite and thirst centers. In both cases, though, overconsumption is clearly confined to the normal rest phase. Such hedonically motivated behavior rhythms are in line with previously reported circadian effects of drug addiction, in which comparable brain regions are involved^28^. Interestingly, not only rodents exhibit elevated drug sensitization during the rest phase^29,30^; drug overdosing in humans also occurs more frequently during that time^31^ and comparable mechanisms for palatable food/liquid intake/addictions are obvious^32^.

CPP and choice experiments, together, suggest two different circadian circuits regulating homeostatic and hedonic appetite rhythms, respectively. This is further corroborated by our finding that clock-deficient mice (here: *Per1/2* double mutants and *Bmal1* knockouts) with dampened homeostatic intake rhythms^33^ show abolished hedonic appetite rhythms even under rhythmic environmental (LD) conditions. Of note, sweet preference—which is sometimes interpreted and defined as liking—was unaffected in both lines.

Hedonic stimuli are thought to activate the dopaminergic mesolimbic system^15^. Although it is well established that dopaminergic circuits regulate addictive behaviors, the physiological relevance of the circadian clock machinery in these brain regions, especially in the VTA, is still under discussion. Firing of dopaminergic neurons and dopamine release show circadian patterns^34,35,40^. Furthermore, genes involved in reward-related behaviors like *Th* are rhythmically expressed in the VTA^40–44^ and the sensitivity of the dopaminergic reward circuit is higher during the rest phase^29^. Our own experiments support these findings showing higher responsivity of dopamine release in the NAc after a chocolate snack in the morning than in the early night. On the other hand, clock-controlled luciferase rhythms are poor in VTA explants^45,46^. Despite this, our transcriptome analysis of VTA punches of WT mice revealed a robust diurnal transcriptome oscillation in the VTA, with ca. 10% rhythmic genes, which is very similar to numbers reported from other tissues^20^. Phasing of core clock gene oscillations in the VTA was comparable to that of other tissues involved in energy metabolism^20^. In clock mutant mice with non-rhythmic hedonic appetite, VTA clock gene expression rhythms were abolished. More interestingly, *Th* mRNA expression had its maximum at the beginning of the active phase^41,42,44^ aligning with the hedonic overconsumption peak in WT mice and correlating with blunted overconsumption rhythms in clock-deficient mice. These findings point to a potential role of VTA clocks in the regulation of hedonic appetite rhythms. Indeed, ablation of VTA clock function by knockdown of the core clock gene *Bmal1* specifically in the VTA ablated hedonic, but not homeostatic, appetite rhythms. In line with the putative role of dopaminergic VTA/NAc communication in addiction and reward behavior^47–50^, mice with VTA-specific *Bmal1* knockdown showed reduced dopamine concentrations in the NAc at the beginning of the rest phase. NAc DOPAC levels were at the same time increased in these mice suggesting altered dopamine turnover at NAc targets. Consistently, mice with dopaminergic neuron-specific *Bmal1* knockdown showed normal homeostatic intake but dampened hedonic overconsumption rhythms. This highlights the importance of the dopaminergic clock in regulation of hedonic appetite rhythms independent of homeostatic MBH circuits or the SCN master pacemaker. Intriguingly, while baseline dopamine levels in the NAc of wild-type animals were higher during the active phase, dopamine responses to palatable food were stronger during the rest phase suggesting that changes in dopamine signaling rather than dopamine tone itself may determine snack intake. Further experiments investigating hedonic snack-induced alterations in NAc dopamine concentrations over the course of the day as well as studies manipulating the dopamine cascade downstream of the VTA will be crucial to fully elucidate the molecular mechanisms regulating circadian appetite rhythms, their interaction with MBH circadian circuits, and to understand the role of dopaminergic clocks in body weight regulation.

Various recent studies in mice and humans suggest that the timing of food intake may be as important for metabolic homeostasis as the amount of energy itself (rodents^3,7,9,51^; humans^10–13^). In the light of increasing chrono-disruption in modern societies—e.g. from shiftwork, artificial lighting or frequent long-distance travel—a better knowledge about the circuits controlling reward-motivated feeding behavior may provide important tools in fighting the worldwide pandemic of obesity and metabolic disorders.

Our data describe a circadian mechanism controlling hedonic appetite independent of homeostatic energy demands and focused on a different phase of the day. Clocks located in the dopaminergic neurons of the VTA define a critical time window sensitizing the organism to the hyperphagic effects of palatable food. This may offer a mechanistic explanation for the observation that access to palatable food results in extended daily feeding periods promoting hyperphagy and circadian rhythm disruption^3,52,53^. While, from an evolutionary perspective, it might be beneficial to prefer high-energy food at the end of the active/beginning of the rest phase, when it can be optimally converted into body mass and for energy storage during sleep, in an environment of food abundance this may become a liability and promote the development of obesity as observed in modern societies worldwide.

## Methods

### Animal models and housing conditions

For all experiments, adult male mice from the breeding colony of the University of Lübeck or the Max-Planck Institute for Metabolism Research in Cologne were used. The transcriptome analysis was performed with C57BL/6N mice. For all other experiments, 2–4 months old mice on a C57BL/6J background—wild-types (WT), clock gene mutants (*Per1/2*-mutants, *Bmal1* knockout (*Bmal1*-KO)), *Bmal1*^*flx/flx*^, and zsGreen reporter mice^54^—were used. Metabolic phenotypes have previously been reported for clock gene mutant mice including lower body weight, but increased adiposity in *Bmal1* deficient^55,56^ and increased body weight and alterations in lipid metabolism in *Per1/2* mutant mice^57,58^. Of note, we did not find significant differences in total food intake or food choice between genotypes in the young mice used in our study (Fig. S1c). All mice were individually housed under standard laboratory conditions with a 12-h light-dark cycle (LD), 22 ± 2 °C and a relative humidity of 60 ± 5% and with ad libitum access to food (breeding chow 2988 kcal/kg; Altromin, Lage, Germany) and water if not stated otherwise. Experiments in constant darkness (DD) where performed on the second day after lights off in mice previously entrained to LD for at least 2 weeks. Different experimental groups were weight- and age-matched. All experimental protocols were approved by the Committee on Animal Health and Care of the State Government of Schleswig-Holstein and were performed according to international guidelines on the ethical use of experimental animals.

### Conditioned place preference

One week prior and during the conditioned place preference (CPP) experiment, food intake of mice was reduced to 85% of their ad libitum food intake (ca. 3 g of chow/day), reducing body weight by about 10%^59^, in order to increase their motivation to search for food. After 1 week, mice were habituated, conditioned, and tested (Fig. 1a) using a three-chamber conditioned place preference system placed in a light-tight and noise-reducing chamber (CPP: TSE Systems, Bad Homburg, Germany). Walls of the two conditioning compartments were uniform gray with orange illumination (30 Lux; left compartment) and black/white striped with yellow illumination (30 Lux; right compartment), respectively. Habituation was performed on 2 consecutive days for 15 min each (day 1 and 2). On day 3, a pre-test, defining place preference, was performed, followed by the conditioning phase from day 4 to 15 and the final test on day 16. To ensure an unbiased CPP^60^, mice with a strong site preference in the pre-test were excluded from further experiments. During the conditioning phase mice of the chow groups were alternately conditioned for 15 min with chow (C^+^; day 4, 6, 8, 10, 12, 14) in the less-preferred compartment. On the following day (C^−^; day 5, 7, 9, 11, 13, 15) mice were conditioning for 15 min in the opposed compartment (preferred compartment during the pre-test) without chow. The cookie groups were equivalent alternately conditioned with cookie (4.95 kcal/g; Coppenrath und Wiese, Osnabrück, Germany) and an opposed empty box. The pre-test, conditioning and CPP of both rest phase groups were performed at ZT4–6 (ZT0 is defined as lights on, ZT12 as lights off), both nighttime groups were pre-tested, conditioned and tested at ZT16–18. Chow/snack intake during these conditioning phases was measured manually. On day 16, CPP was investigated (by using TSE LabMaster Place Preference V5.5.9) and preference index (PI) of the first 5 min was calculated (PI = time spent in snack-conditioned box/time spent in other compartments).

### Homeostatic and hedonic food intake

Homeostatic food intake was measured in ad libitum-fed mice in 6-h intervals either under standard LD conditions or on the second day in DD. For defining hedonic appetite mice had access to chocolate snacks (Milka Naps Milk Chocolate; 5.3 kcal/g; Mondelēz International, East Hanover, USA) ad libitum and additionally to chow. After an overnight habituation to the snack, chow and chocolate intake were measured in 6-h intervals over the course of the day and chocolate preference (chocolate intake/total intake [chow + chocolate]) as well as overconsumption (total caloric intake [chow + chocolate]/homeostatic ad libitum chow caloric intake; Fig. 2a) were calculated.

### Homeostatic and hedonic drinking behavior

Homeostatic and hedonic drinking behaviors were measured in choice lickometer cages equipped with two bottles (Campden Instruments, Loughborough, UK) under standard LD conditions and on the second and third day in DD. For homeostatic intake, licks (measured by using Scurry Activity System 1.7.0.0) of both bottles filled with tap water were determined at 6-h intervals over 2 days. Afterward, the less-preferred bottle was exchanged with a bottle containing either 10% sucrose or 1% sucralose in tap water and licking behavior from both bottles was recorded. Overconsumption and hedonic preference were calculated as described for food intake measuring licks instead of calories.

### Relative mRNA expression

Brain tissue for quantitative real-time PCR (qPCR) was harvested either from decapitated mice (WT and *Per1/2*; Fig. 4) or PFA-perfused mice (VTA-KD; Fig. 5). After decapitation, brains were quickly removed, frozen on dry ice and cryo-sectioned (12 µm; CM3050S, Leica Biosystems, Nussloch, Germany). VTA containing brain sections were placed on membrane slides (Membran Slide 1.0 PEN, Carl Zeiss, Oberkochen, Germany) and VTA was dissected using a laser dissection microscope (Palm Microbeam, Zeiss Microimaging, Oberkochen, Germany). Of each mouse, three dissected VTA sections were pooled. Total RNA was extracted using Direct-zol RNA Micro-Prep kit (Zymo Research, Irvine, USA) according to the manufacturer’s protocol. cDNA synthesis was performed using the High-Capacity cDNA Reverse Transcription Kit (Life Technologies, Carlsbad, USA) with random hexamer primers followed by qPCR reaction (CFX96 thermocycler, Bio-Rad, Munich, Germany, software: Bio-Rad CFX Manager 3.0) using GoTaq qPCR Master Mix (Promega, Madison, USA). Relative gene expression was quantified using the ΔΔ threshold cycle (Ct) method^61^. *Eef1α* was used as reference gene for all experiments. Primer sequences are provided in Supplementary Table 1). PFA-perfused brains were removed, sucrose dehydrated (30%) and frozen in dry ice cooled isopentane followed by cryo-sectioning (20 µm, for details see immunohistochemistry below). Laser-dissected VTA sections (3 per mouse) were pooled and mRNA isolated using RNeasy FFPE Kit (Qiagen, Hilden, Germany) according to the manufacturer’s protocol. cDNA synthesis and qPCRs were performed as explained above.

### Transcriptome analysis

Under standard LD conditions, C57BL/6N mice were sacrificed at 6-h intervals (4 mice over a period of 24 h, brains removed and shock-frozen in liquid nitrogen. Using a brain slicer, 0.5-mm sections including the VTA were prepared. VTA was punched and total RNA was isolated from the tissue using RNeasy Mini Kit (Qiagen, Hilden Germany) according to the manufacturer’s protocol. cDNA synthesis, labeling, and hybridization (GeneChip Mouse Gene 1.0 ST Array Affymetrix, Santa Clara, USA) were performed by the Cologne Center for Genomics/Cellular Stress Responses in Aging-Associated Diseases (CCG/CECAD) according to standard protocols. Microarray data were deposited in the GEO database (GSE125812). Raw data were normalized to the average of each gene followed by circadian rhythm analysis performed with CircWave with a *p*-value cut-off of 0.05^62^. Peak time calculation in 0.1-h intervals was performed by sine wave regression. Rhythmic genes were sorted by phase and visualized in a double-plotted heat map using the matrix2png interface. Peak time comparison of core clock genes in the VTA was performed with published gene expression data sets (SCN^63^, liver^8^, eWAT^64^) enrichment analysis was performed using WebGestalt^65^.

### Dopamine and DOPAC quantification

Tissue concentrations of dopamine and its metabolite, 3,4-dihydroxyphenylacetic acid (DOPAC), were determined by high-performance liquid chromatography (HPLC). After decapitation, brains were removed and shock-frozen on dry ice. Brains were sliced on ice in 1 mm thin sections including the NAc, which was bilaterally punched. NAc samples were homogenized in PBS/PCA (100 µl PBS + 5 µl perchloric acid, 3.3%) followed by centrifugation (4 °C, 15,000 × *g*). Remaining supernatant was investigated for the content of dopamine and its metabolite DOPAC using a C18 column (Eurosphere 100, 5 µm, column size 250 × 4 mm) and a precolumn (30 × 4 mm). The isocratic mobile phase (0.1 M citric acid monohydrate, 0.3 mM Na_2_EDTA, 0.5 mM sodium octane-sulfonate, 11.5% methanol, pH 3.0, degassed by helium) was pumped at a flow rate of 1.0 ml/min at 3 °C. The compound was detected electrochemically using a glassy carbon electrode set at a potential of 800 mV relative to an Ag/AgCl reference electrode (electrochemical detector type CLC 100, Chromsystems, Gräfelfing, Germany) with a detection limit of 100 fmol/injection.

### Central manipulation of VTA clocks

To genetically manipulate clock function specifically in the VTA or in VTA dopaminergic neurons, 0.5 µl of a CRE-expressing adeno-associated virus (VTA-KD: AAV/DJ-GFP-iCRE, titer 0.5 × 10^13^ GC/ml; dopaminergic neuron-specific-KD: AAV2-rTHp-iCRE, titer 6.8 × 10^12^ GC/ml; Vector Biolabs, Malvern, USA) or AAVs of the same serotypes carrying a scrambled Cre cDNA sequence as controls were bilaterally injected into the VTA (coordinates: ±0.5 mm lateral, 2.8 mm posterior, and 4.75 mm ventral relative to Bregma) of *Bmal1*^*flx/flx*^ or zsGreen reporter mice. The injection was performed over 2 min and the needle was held in place for another 5 min to prevent backflow of virus solution. Five to seven weeks after manipulation food intake measurements, knockdown validation and dopamine quantifications were performed.

### Microdialysis in the NAc

Two holes were drilled in the back of the right and left hemisphere, respectively, to insert two screws (00-96x 1/16, Bilaney Consultants, Düsseldorf, Germany) as an additional hold for the cannula. The microdialysis cannula (CMA7, shaft length 3.9 mm, closed with a dummy, Harvard Apparatus, Cambridge, USA) was implanted anterior of the NAc (coordinates −1.25 mm lateral to midline, 1.5 mm anterior to Bregma, −3.9 mm ventral) and fixed with dental cement. The probe (CMA7 1 mm cuprophane membrane, cut-off 6 kDa, shaft length 3.9 mm, Harvard Apparatus) was 1 mm longer, targeting the NAc directly (coordinates: −1.25 mm lateral to midline, 1.5 mm anterior to Bregma, −4.9 mm ventral). Mice were accustomed to the microdialysis setting, a transparent half-shell, three times for 30 min prior to the microdialysis. Tubings were flushed with the perfusate (125 mM sodium chloride, 5 mM potassium chloride, 2 mM calcium chloride dehydrate, 1.25 mM potassium dihydrogen phosphate, 1 mM magnesium sulfate heptahydrate, 25 mM sodium hydrogen carbonate, and 0.01 mM ascorbic acid, in Milli-Q purified water, pH adjusted to 7.4 with 85% orthophosphoric acid, sterile filtration 0.2 µm and sonicated) at a flow rate of 13 µL/min. Mice belonging to the ZT0 group were fasted at ZT8 the day before microdialysis, mice of the ZT12 group at ZT5.5 on the day of experiment. For the microdialysis, mice were placed in the half-shell provided with bedding material and 1% agarose gel as water source. Microdialysis was started with a 1-h probe equilibration time at ZT0 or ZT12, respectively, with a flow rate of 0.6 µL/min. Afterward, microdialysate was collected in BSA-coated tubes in 1.8 µL 1 mM ascorbic acid on ice from ZT1–3/13–15 in 20 min sampling intervals. At ZT2.33/14.33 a chocolate snack was provided (four RUF milk chocolate drops, ~0.58 g, RUF, Quakenbrück, Germany). To determine the effect of the chocolate snack on dopamine release, microdialysate was collected from ZT2.66–4.33/14.66–16.33 in 20-min intervals. For dopamine quantifications 5 samples (20 min each) were pooled for baseline measurements and after chocolate concentrations, respectively, and analyzed by ELISA (NOVUS Biologicals, Centennial, USA, cat. no. NBP2-67270). Snack-induced changes in dopamine levels were calculated against pre-chocolate baseline levels for each individual and expressed as percent change with negative values indicating a decrease in dopamine concentrations. For post-experimental confirmation of cannula positioning, brains were sectioned in coronal slices (20 µm) with a cryostat (Leica) and stained with hematoxylin and eosin.

### Statistical analysis

Statistical differences were investigated by two-tailed *t*-test or two-way ANOVAs with post hoc tests (as stated) using GraphPad Prism software (GraphPad, La Jolla, United States). Oscillations of intake or transcriptome data were analyzed with CircWave^62^. PIs of CPP experiments were further investigated by one-sample *t*-test against chance level using Minitab17 (Additive GmbH, Friedrichsdorf, Germany).

### Reporting summary

Further information on research design is available in the Nature Research Reporting Summary linked to this article.

## Supplementary information

Supplementary Information

Peer Review File

Reporting Summary

Supplementary Dataset 1

## Data Availability

Microarray data are accessible via GEO database (GSE125812). Source data for Figs. 1–5 and Supplementary Figs. 1–4 are provided as a Source data file. Source data are provided with this paper.

## Source data

Source Data
