# Supplementary material for: Circadian regulation of hedonic appetite in mice by clocks in dopaminergic neurons of the VTA
Source: Nat Commun. 2020 Jun 17;11:3071. doi: 10.1038/s41467-020-16882-6 (PMC7299974; doi:10.1038/s41467-020-16882-6)
Supplement: Supplementary file 1 — Supplementary Information [file 41467_2020_16882_MOESM1_ESM.pdf]

Koch, Begemann et al.

# Circadian regulation of hedonic appetite in mice by clocks in dopaminergic neurons of the VTA

## Supplementary Material

*Contents:*

Supplementary Figures 1-4
Supplementary Table 1

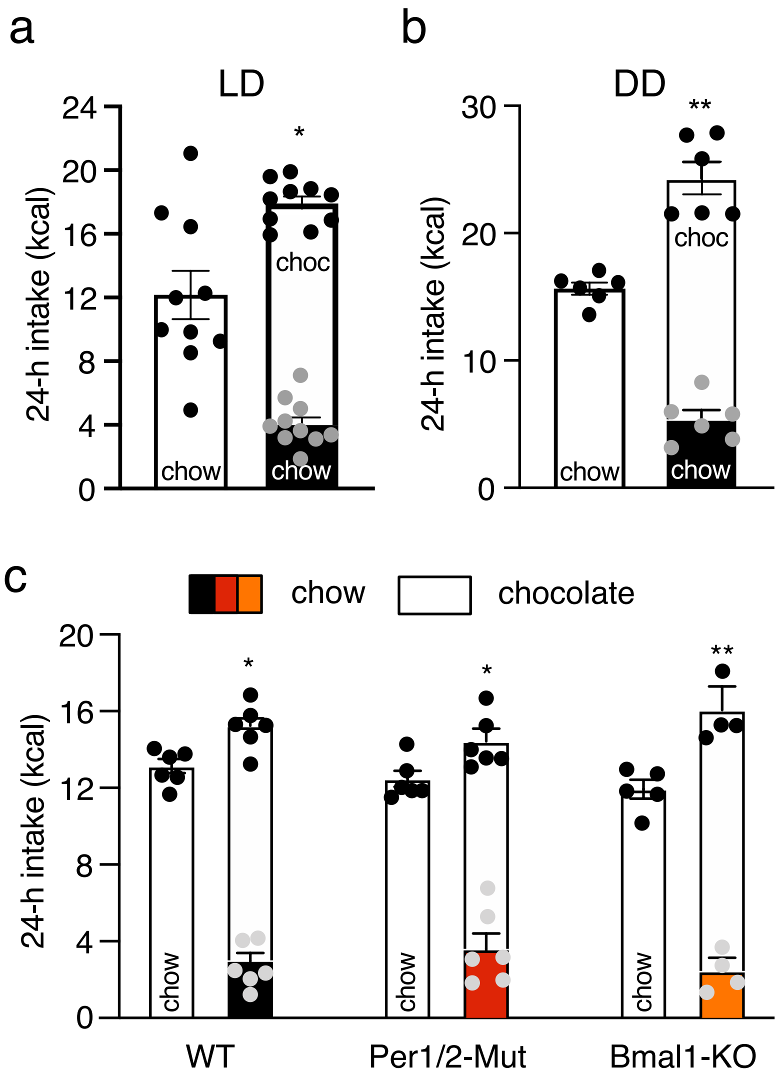


**Supplementary Fig. 1: Caloric intake of WT and clock mutant mice.**

a, b) Cumulative caloric intake of WT mice with *ab libitum* access to chow only (left bar) or *ad libitum* choice access to chow & chocolate (right bar) under standard light/dark conditions (LD) (a; n = 10) and in DD (b; n = 6). c) Cumulative caloric intake of WT (n = 5), *Per1/2* double mutants (n = 6) and *Bmal1* KO mice (n = 4) with either *ad libitum* access to chow only (left) or *ad libitum* choice access to chow & chocolate (right) under standard LD conditions. Data are shown as mean ± SEM; t-test (a & b) or two-way ANOVA (c) with *post-hoc* test, * p< 0.05; ** p < 0.01 (a: p = 0.0201; b: p = 0.0032; c: WT p = 0.0451, Per1/2-Mut p = 0.0413, Bmal1-KO p = 0.0277). All replicates are biologically independent samples. Source data are provided as a Source Data file.

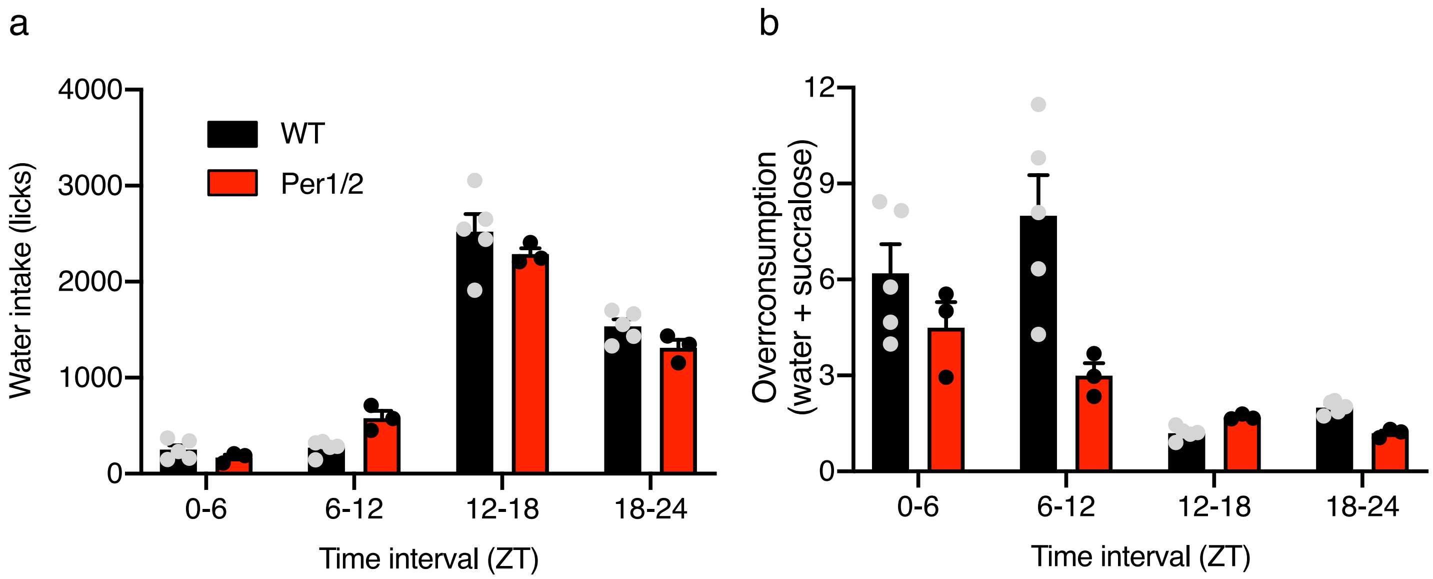


**Supplementary Fig.2: Hedonic overconsumption of WT and *Per1/2* double mutants.**

Homeostatic water intake (licks) (a) and sucralose & water (b) overconsumption of WT (black, n = 5) and *Per1/2* double-mutant mice (red, n = 3). Data are shown as mean ± SEM; two-way ANOVA with *post-hoc* test; WT *vs. Per1/2*; white box = light phase, black box = dark phase; dashed line depicting homeostatic water intake. All replicates are biologically independent samples. Source data are provided as a Source Data file.

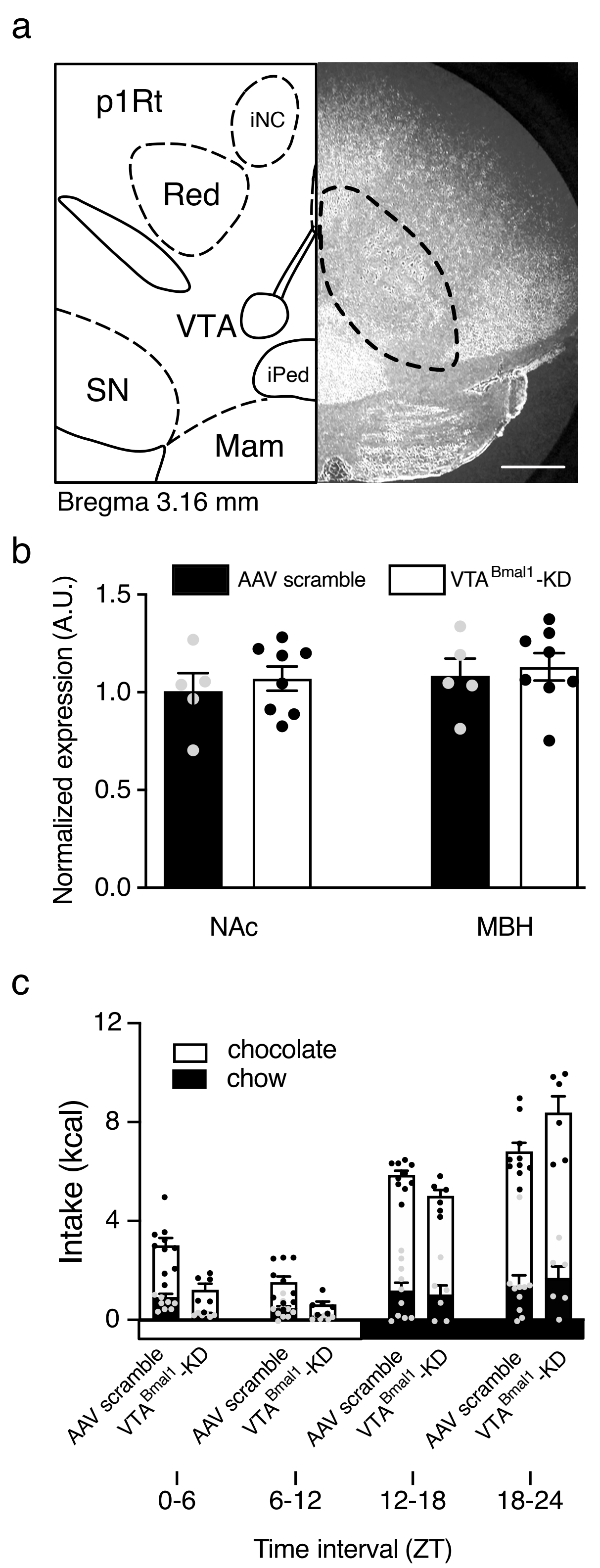


**Supplementary Fig.3: Validation of knockdown and choice intake in mice with specific knockdown of *Bmal1* in VTA neurons.**

a) Diagram depicting excision area (dashed oval on the right) for VTA-specific quantification of gene expression (white bar indicates 200 µm). Micrographs were taken prior to dissection for all animals (n = 13). b) *Bmal1* mRNA expression in the NAc and the MBH of VTA$^{Bmal1}$-KD mice (n = 8) and controls (n = 5). c) Chocolate/chow choice intake rhythms of VTA$^{Bmal1}$-KD mice (n = 8) and controls (*n* = 5). Data are shown as mean ± SEM. White box = light phase, black box = dark phase; p1Rt – p1 reticular formation; iNC – interstitial nucleus of Cajal; Red – red nucleus; iPed – interpeduncular nucleus; SN – substantia nigra; Mam – mammillary nucleus. All replicates are biologically independent samples. Source data are provided as a Source Data file.

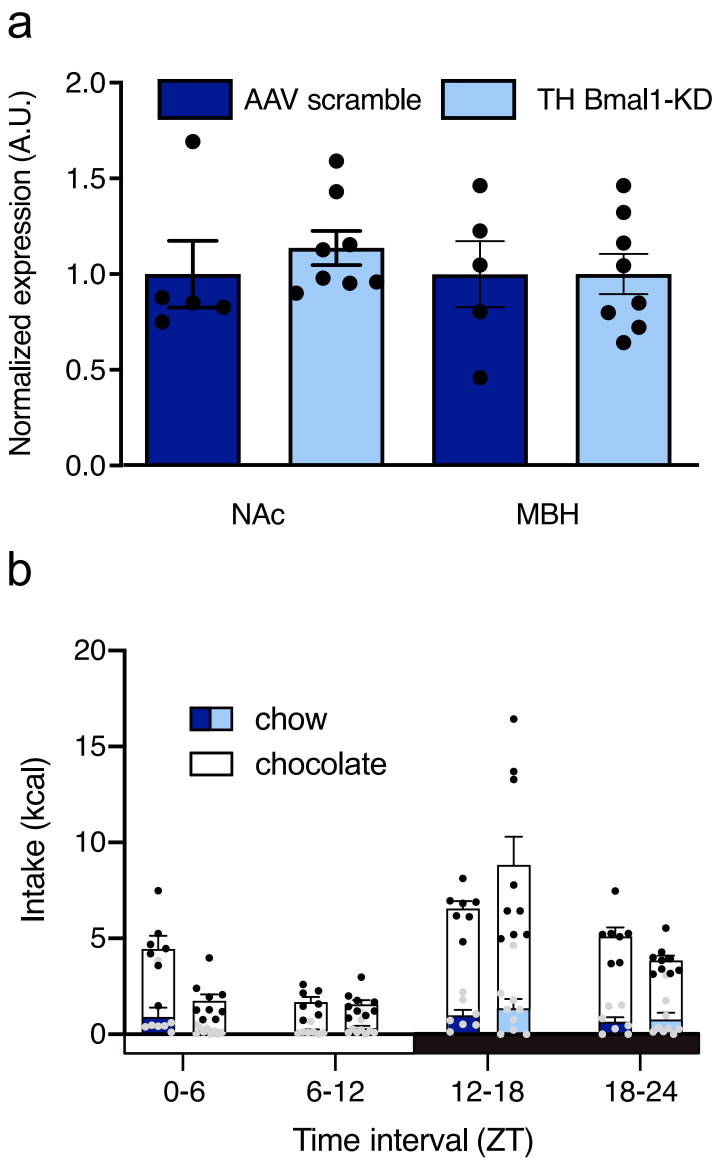


**Supplementary Fig.4: Validation of knockdown and choice intake in mice with specific knockdown of *Bmal1* in TH neurons.**

a) *Bmal1* mRNA expression in the NAc and the MBH of $TH^{Bmal1}$-KD mice (n = 9) and controls (n = 8). A) Chocolate/chow choice intake rhythms of $TH^{Bmal1}$-KD mice (n = 9) and controls (n = 8). Data are shown as mean ± SEM. White box = light phase, black box = dark phase. All replicates are biologically independent samples. Source data are provided as a Source Data file.

**Supplementary Table 1:** Primer sequences

| Transcript | Forward primer (5'->3') | Reverse primer (5'->3') |
|---|---|---|
| *Bmal1* | *CCTAATTCTCAGGGCAGCAGAT* | *TCCAGTCTTGGCATCAATGAGT* |
| *Cre* | *CCCTGTTTCACTATCCAGGT* | *GGGTAACTAAACTGGTCGAG* |
| *Eef1a* | *TGCCCCAGGACACAGAGACTTCA* | *AATTCACCAACACCAGCAGCAA* |
| *Per2* | *GCCAAGTTTGTGGAGATTCCTG* | *CTTGCACCTTGACCAGGTAGG* |
| *Rev-erb alpha* | *AGCTCAACTCCCTGGCACTTAC* | *CTTCTCGGAATGCATGTTGTTC* |
| *Th* | *TCTCCTTGAGGGGTACAAAACC* | *ACCTCGAAGCGCACAAAGT* |
