# Supplementary material for: Circadian regulation of hedonic appetite in mice by clocks in dopaminergic neurons of the VTA
Source: Nat Commun. 2020 Jun 17;11:3071. doi: 10.1038/s41467-020-16882-6 (PMC7299974; doi:10.1038/s41467-020-16882-6)
Supplement: Supplementary file 3 — Reporting Summary [file 41467_2020_16882_MOESM3_ESM.pdf]

nature research

Corresponding author(s): Henrik Oster

Last updated by author(s): May 22, 2020

Reporting Summary

Nature Research wishes to improve the reproducibility of the work that we publish. This form provides structure for consistency and transparency in reporting. For further information on Nature Research policies, see Authors & Referees and the Editorial Policy Checklist.

Statistics

For all statistical analyses, confirm that the following items are present in the figure legend, table legend, main text, or Methods section.

n/a	Confirmed	
☐	☒	The exact sample size (*n*) for each experimental group/condition, given as a discrete number and unit of measurement
☐	☒	A statement on whether measurements were taken from distinct samples or whether the same sample was measured repeatedly
☐	☒	The statistical test(s) used AND whether they are one- or two-sided<br>*Only common tests should be described solely by name; describe more complex techniques in the Methods section.*
☐	☒	A description of all covariates tested
☐	☒	A description of any assumptions or corrections, such as tests of normality and adjustment for multiple comparisons
☐	☒	A full description of the statistical parameters including central tendency (e.g. means) or other basic estimates (e.g. regression coefficient) AND variation (e.g. standard deviation) or associated estimates of uncertainty (e.g. confidence intervals)
☐	☒	For null hypothesis testing, the test statistic (e.g. *F*, *t*, *r*) with confidence intervals, effect sizes, degrees of freedom and *P* value noted<br>*Give P values as exact values whenever suitable.*
☒	☐	For Bayesian analysis, information on the choice of priors and Markov chain Monte Carlo settings
☒	☐	For hierarchical and complex designs, identification of the appropriate level for tests and full reporting of outcomes
☒	☐	Estimates of effect sizes (e.g. Cohen's *d*, Pearson's *r*), indicating how they were calculated

Our web collection on statistics for biologists contains articles on many of the points above.

Software and code

Policy information about availability of computer code

Data collection	Water intake: Scurry Activity System 1.7.0.0; CPP: TSE LabMaster Place Preference V5.5.9; qPCR: Bio-Rad CFX Manager 3.0
Data analysis	GraphPad Prism 5,7 & 8; CircWave V3.3; Minitab 17

For manuscripts utilizing custom algorithms or software that are central to the research but not yet described in published literature, software must be made available to editors/reviewers. We strongly encourage code deposition in a community repository (e.g. GitHub). See the Nature Research guidelines for submitting code & software for further information.

Data

Policy information about availability of data

All manuscripts must include a data availability statement. This statement should provide the following information, where applicable:

- Accession codes, unique identifiers, or web links for publicly available datasets
- A list of figures that have associated raw data
- A description of any restrictions on data availability

Transcriptome data: GEO GSE125812

Field-specific reporting

Please select the one below that is the best fit for your research. If you are not sure, read the appropriate sections before making your selection.

☒ Life sciences ☐ Behavioural & social sciences ☐ Ecological, evolutionary & environmental sciences

For a reference copy of the document with all sections, see nature.com/documents/nr-reporting-summary-flat.pdf

Life sciences study design

All studies must disclose on these points even when the disclosure is negative.

Sample size	Sample sizes were selected in accordance with previous experiments and publications in the circadian/behavioral field. 24-h transcriptome profiles are typically determined with n=2-3 (e.g. Panda et al. Cell. 2002 May 3;109(3):307-20; Husse et al. Sleep. 2017 Jun 1;40(6).) per time point and selected results were validated by qPCR on different samples (n = 3-5; e.g. Cho et al. Nature. 2012 Mar 29;485(7396):123-7). Due to the individual variability in behavioral studies more replicates were necessary for CPP (n = 10; e.g. Wan et al. Neuroscience. 2017 Mar 6;344:67-73; Zhou et al. Nat Neurosci. 2019 Dec;22(12):1986-1999) and intake experiments (n = 8-14; e.g. Minaya et al. Physiol Behav. 2016 Oct 1;164(Pt A):395-9; Liu et al. Nat Protoc. 2018 Jul;13(7):1686-1698. Following the 3R ethical principle less animals were used when effect sizes were larger than expected and results became significant. Since the intake studies were independently repeated with different mutant lines and respective controls with comparable results, we assume that cohort sizes were sufficient here.
Data exclusions	No data were excluded from the analyses.
Replication	In all cases a minimum of 3 (up to 14) biological replicates were used as indicated. All replicates were included in the analysis. Behavioral assays (Figs. 1-3, 5) were done in at least two independent experiments (with comparable outcomes) and results pooled.
Randomization	To exclude weight and age effects, all mice were age- and weight-matched and then randomly designed to one group.
Blinding	Investigators were blinded during all data collection and analysis whenever possible. Blinded data collection during CPP was not possible since light conditions and snack treat treatment were different between groups. CPP data analyses were blinded.

Reporting for specific materials, systems and methods

We require information from authors about some types of materials, experimental systems and methods used in many studies. Here, indicate whether each material, system or method listed is relevant to your study. If you are not sure if a list item applies to your research, read the appropriate section before selecting a response.

Materials & experimental systems

n/a	Involved in the study	
☒	☐	Antibodies
☒	☐	Eukaryotic cell lines
☒	☐	Palaeontology
☐	☒	Animals and other organisms
☒	☐	Human research participants
☒	☐	Clinical data

Methods

n/a	Involved in the study	
☒	☐	ChIP-seq
☒	☐	Flow cytometry
☒	☐	MRI-based neuroimaging

Animals and other organisms

Policy information about studies involving animals; ARRIVE guidelines recommended for reporting animal research

Laboratory animals	For all behavioral experiments mixed-gender mice of 2-4 months of age on a C57/BL6J background were used. Genotype details are provided in the methods section.
Wild animals	No wild animals were used in the study
Field-collected samples	No field-collected samples were used in the study
Ethics oversight	All experimental protocols were approved by the Committee on Animal Health and Care of the State Government of Schleswig-Holstein and were performed according to international guidelines on the ethical use of experimental animals.

Note that full information on the approval of the study protocol must also be provided in the manuscript.
