# Supplementary material for: Circadian regulation of hedonic appetite in mice by clocks in dopaminergic neurons of the VTA
Source: Nat Commun. 2020 Jun 17;11:3071. doi: 10.1038/s41467-020-16882-6 (PMC7299974; doi:10.1038/s41467-020-16882-6)
Supplement: Supplementary file 4 — Supplementary Dataset 1 [file 41467_2020_16882_MOESM4_ESM.pdf]

Koch, Begemann et al.

Circadian regulation of hedonic appetite in mice by clocks in dopaminergic neurons of the VTA

Supplementary Data Set 1

List of rhythmic genes in the VTA

Probe Set ID	CircWave p-value	rel. Amplitude	Peak phase	Gene ID
10339901	0.0434	1.051	1	---
10339418	0.0034	0.958	1.6	---
10342835	0.0300	0.691	18.9	---
10342570	0.0092	0.638	1	---
10338807	0.0309	0.631	2	---
10344488	0.0064	0.609	18.3	---
10343814	0.0135	0.601	16.7	---
10338461	0.0111	0.529	4.1	---
10343847	0.0050	0.524	1	---
10338103	0.0183	0.512	1.1	---
10338149	0.0013	0.496	14.3	---
10362442	0.0045	0.483	17	*Trdn*
10343863	0.0045	0.483	13.4	---
10341305	0.0207	0.474	17	---
10344515	0.0219	0.459	2.4	---
10342841	0.0202	0.454	24	---
10344101	0.0308	0.448	6.5	---
10343187	0.0218	0.445	16.5	---
10344215	0.0251	0.441	1.9	---
10343757	0.0349	0.435	10.2	---
10450731	0.0171	0.417	2.3	*Gm7030*
10341547	0.0198	0.414	1.3	---
10342063	0.0262	0.414	1.2	---
10343813	0.0066	0.409	4.3	---
10342112	0.0450	0.408	1	---
10341357	0.0016	0.408	1.5	---
10340377	0.0356	0.407	17.7	---
10342401	0.0168	0.406	1.4	---
10338855	0.0198	0.405	1.2	---
10341742	0.0417	0.404	19.5	---
10339994	0.0308	0.399	1.8	---
10343395	0.0159	0.398	1	---
10341168	0.0130	0.395	15.4	---
10342291	0.0383	0.394	21.8	---
10341586	0.0247	0.392	16.6	---
10340000	0.0014	0.390	18.7	---
10338194	0.0353	0.382	16.4	---
10338341	0.0210	0.382	2.1	---
10339147	0.0132	0.381	1.9	---
10342873	0.0274	0.381	15.4	---
10598079	0.0029	0.375	3.6	*mt-Tc*
10582888	0.0189	0.373	2.1	*Gm10721*
10339259	0.0204	0.373	10.9	---
10342380	0.0100	0.373	15.6	---
10338863	0.0008	0.369	16	---
10343740	0.0024	0.366	14.8	---
10340369	0.0189	0.366	1.3	---

10339651	0.0255	0.365	1.6	---
10339935	0.0164	0.365	2.8	---
10343667	0.0085	0.364	14.4	---
10343206	0.0157	0.363	15.2	---
10505917	0.0305	0.360	3.6	*Gm12643*
10344608	0.0392	0.360	17.8	---
10340942	0.0223	0.359	2.2	---
10345436	0.0234	0.358	1.7	---
10346222	0.0234	0.358	1.7	---
10356762	0.0234	0.358	1.7	---
10357298	0.0234	0.358	1.7	---
10359642	0.0234	0.358	1.7	---
10363005	0.0234	0.358	1.7	---
10363561	0.0234	0.358	1.7	---
10374183	0.0234	0.358	1.7	---
10374352	0.0234	0.358	1.7	---
10375324	0.0234	0.358	1.7	---
10395275	0.0234	0.358	1.7	---
10410927	0.0234	0.358	1.7	---
10416696	0.0234	0.358	1.7	---
10416698	0.0234	0.358	1.7	---
10422247	0.0234	0.358	1.7	---
10424377	0.0234	0.358	1.7	---
10427389	0.0234	0.358	1.7	---
10448230	0.0234	0.358	1.7	---
10457667	0.0234	0.358	1.7	---
10468487	0.0234	0.358	1.7	---
10469127	0.0234	0.358	1.7	---
10476399	0.0234	0.358	1.7	---
10478746	0.0234	0.358	1.7	---
10483161	0.0234	0.358	1.7	---
10484355	0.0234	0.358	1.7	---
10485355	0.0234	0.358	1.7	---
10489721	0.0234	0.358	1.7	---
10496167	0.0234	0.358	1.7	---
10496336	0.0234	0.358	1.7	---
10497327	0.0234	0.358	1.7	---
10500527	0.0234	0.358	1.7	---
10512463	0.0234	0.358	1.7	---
10522742	0.0234	0.358	1.7	---
10527425	0.0234	0.358	1.7	---
10528165	0.0234	0.358	1.7	---
10528205	0.0234	0.358	1.7	---
10540531	0.0234	0.358	1.7	---
10541129	0.0234	0.358	1.7	---
10543029	0.0234	0.358	1.7	---
10565598	0.0234	0.358	1.7	---
10568534	0.0234	0.358	1.7	---

10574432	0.0234	0.358	1.7	---
10574434	0.0234	0.358	1.7	---
10581009	0.0234	0.358	1.7	---
10582983	0.0234	0.358	1.7	---
10586863	0.0234	0.358	1.7	---
10589974	0.0234	0.358	1.7	---
10603549	0.0234	0.358	1.7	---
10603803	0.0234	0.358	1.7	---
10607429	0.0234	0.358	1.7	---
10338934	0.0118	0.358	2.2	---
10363224	0.0000	0.357	4.2	*Fabp7*
10340099	0.0181	0.355	14.4	---
10344562	0.0062	0.353	1.1	---
10339604	0.0263	0.353	1.9	---
10343229	0.0265	0.353	6.6	---
10341563	0.0370	0.352	22.9	---
10342971	0.0011	0.351	15.2	---
10598071	0.0100	0.349	2.4	*mt-Tt*
10339223	0.0035	0.349	1.8	---
10340761	0.0096	0.347	1.3	---
10341118	0.0348	0.347	18.5	---
10338365	0.0099	0.347	4.6	---
10338099	0.0264	0.341	3.1	---
10342711	0.0002	0.340	15.3	---
10341243	0.0374	0.338	12.8	---
10342439	0.0110	0.336	2.2	---
10344218	0.0221	0.334	17.6	---
10343807	0.0219	0.334	21.9	---
10343689	0.0113	0.334	2.4	---
10343193	0.0175	0.334	16.3	---
10342625	0.0106	0.334	1.5	---
10342679	0.0146	0.332	15.1	---
10342854	0.0095	0.332	12.9	---
10344548	0.0311	0.331	16.9	---
10342241	0.0367	0.331	13.4	---
10340228	0.0067	0.331	15.1	---
10341661	0.0432	0.330	20.2	---
10338635	0.0428	0.330	15	---
10339336	0.0308	0.329	17.3	---
10598053	0.0029	0.328	2.2	*mt-Tg*
10339362	0.0388	0.328	19.1	---
10340310	0.0150	0.325	17.1	---
10341573	0.0267	0.324	20.3	---
10339513	0.0113	0.323	14.9	---
10339010	0.0063	0.321	16	---
10417068	0.0132	0.321	16.3	---
10582916	0.0424	0.320	3.9	*Gm17535*
10342317	0.0454	0.319	6.9	---

10339347	0.0195	0.318	15.4	---
10344496	0.0018	0.318	23.1	---
10338873	0.0006	0.317	14.4	---
10343104	0.0054	0.317	1	---
10340977	0.0005	0.315	15.8	---
10339631	0.0494	0.313	18	---
10338250	0.0056	0.313	15.4	---
10339503	0.0153	0.312	21.1	---
10338623	0.0115	0.312	15.8	---
10343260	0.0216	0.312	20.8	---
10341219	0.0072	0.310	2.1	---
10344064	0.0470	0.310	1.6	---
10343650	0.0312	0.310	24	---
10343090	0.0065	0.308	16.2	---
10341743	0.0238	0.308	17.2	---
10339476	0.0304	0.308	16.2	---
10338109	0.0121	0.305	11.1	---
10341509	0.0472	0.304	18.5	---
10338370	0.0213	0.304	2	---
10339154	0.0042	0.303	16.4	---
10343179	0.0454	0.302	17.8	---
10594798	0.0385	0.302	5.1	*Gm23730*
10342148	0.0395	0.301	12.4	---
10344574	0.0161	0.301	19.1	---
10338181	0.0352	0.300	11	---
10338771	0.0137	0.298	14.6	---
10417458	0.0283	0.297	1	
10339602	0.0394	0.297	8.4	---
10338964	0.0416	0.297	12	---
10362073	0.0002	0.297	14.4	*Sgk1*
10342440	0.0281	0.297	15.3	---
10341285	0.0222	0.296	17.5	---
10342574	0.0355	0.296	3.3	---
10608579	0.0031	0.296	2.7	*LOC102639094*
10338395	0.0143	0.295	6.4	---
10343066	0.0160	0.295	16.2	---
10340327	0.0462	0.294	15.9	---
10343684	0.0072	0.294	15.9	---
10341387	0.0462	0.293	14.7	---
10342664	0.0325	0.293	1.6	---
10340091	0.0468	0.293	20.9	---
10340583	0.0174	0.292	12.5	---
10338345	0.0332	0.290	13.3	---
10338546	0.0177	0.290	17.8	---
10341810	0.0146	0.289	15.8	---
10339705	0.0032	0.289	15.6	---
10343957	0.0160	0.289	19.1	---
10339169	0.0008	0.287	2.6	---

10341425	0.0470	0.287	20.4	---
10342547	0.0219	0.287	16.5	---
10342950	0.0207	0.287	2.2	---
10343247	0.0378	0.286	1.5	---
10340043	0.0320	0.285	1.9	---
10340939	0.0494	0.285	1.4	---
10342457	0.0124	0.284	8.1	---
10342200	0.0077	0.282	17.7	---
10341287	0.0245	0.282	9.1	---
10338979	0.0080	0.282	3	---
10339945	0.0284	0.282	15.1	---
10339373	0.0311	0.282	14.4	---
10340426	0.0351	0.281	15	---
10342296	0.0312	0.281	16.5	---
10339197	0.0201	0.281	7	---
10344340	0.0360	0.280	22.1	---
10598077	0.0028	0.279	2.4	*mt-Tn*
10340138	0.0438	0.279	14.1	---
10342600	0.0189	0.278	15.4	---
10545239	0.0100	0.278	11.2	*LOC637260*
10338726	0.0237	0.277	15.9	---
10338913	0.0039	0.277	16.5	---
10340313	0.0201	0.277	14.7	---
10344569	0.0050	0.277	23.4	---
10427459	0.0096	0.276	3.8	*Gm10250*
10341437	0.0407	0.275	1.3	---
10339164	0.0132	0.275	11	---
10342691	0.0129	0.274	13.2	---
10340096	0.0213	0.272	1	---
10417408	0.0376	0.271	1	
10339816	0.0308	0.271	3	---
10339914	0.0342	0.270	7.1	---
10339887	0.0353	0.270	13.8	---
10484514	0.0227	0.270	17.6	*Olfr993*
10341625	0.0256	0.269	12.2	---
10339626	0.0403	0.268	20.4	---
10343706	0.0105	0.268	1	---
10341119	0.0370	0.268	16.7	---
10341918	0.0412	0.267	3.7	---
10340756	0.0382	0.265	15.3	---
10417501	0.0324	0.265	1	
10343786	0.0448	0.264	23.3	---
10354416	0.0045	0.263	15	---
10341176	0.0347	0.263	5.9	---
10343552	0.0013	0.262	2	---
10339015	0.0245	0.262	15	---
10344057	0.0268	0.261	16.3	---
10338843	0.0381	0.261	10.9	---

10386418	0.0276	0.261	2.4	*Gm12261*
10342592	0.0252	0.259	18.2	---
10339738	0.0010	0.259	1.2	---
10344499	0.0438	0.258	20.7	---
10342085	0.0156	0.257	22.1	---
10454731	0.0136	0.257	3.4	*n-R5s25*
10340469	0.0256	0.257	11.6	---
10338612	0.0427	0.256	18.6	---
10405969	0.0410	0.256	2.1	*Gm4812*
10343128	0.0183	0.255	2.5	---
10338878	0.0191	0.255	15.2	---
10342419	0.0037	0.254	16.7	---
10338277	0.0315	0.254	3.3	---
10342531	0.0043	0.254	14.9	---
10585697	0.0093	0.253	1.9	*Gm5121*
10343967	0.0446	0.252	14.8	---
10344154	0.0258	0.251	15.4	---
10340558	0.0399	0.251	1.5	---
10340525	0.0173	0.250	19.4	---
10340493	0.0275	0.250	21.7	---
10341027	0.0333	0.249	3.9	---
10338487	0.0383	0.249	14.9	---
10338482	0.0085	0.249	1	---
10340945	0.0292	0.249	14.9	---
10342082	0.0112	0.248	16.8	---
10342665	0.0013	0.247	17	---
10417411	0.0430	0.247	1	*Gm3002*
10353667	0.0046	0.246	15.3	*Gm22260*
10343603	0.0263	0.246	24	---
10340174	0.0457	0.246	12.7	---
10344087	0.0347	0.245	18	---
10598057	0.0108	0.245	2.3	*mt-Tr*
10339905	0.0496	0.245	2.5	---
10343644	0.0375	0.244	1	---
10343127	0.0350	0.243	16.7	---
10342470	0.0121	0.243	12.5	---
10344159	0.0409	0.243	14.9	---
10342462	0.0069	0.243	15.5	---
10344023	0.0213	0.242	14.2	---
10339083	0.0077	0.242	23.9	---
10339765	0.0415	0.242	23.9	---
10341591	0.0153	0.242	14.1	---
10341239	0.0482	0.242	1.8	---
10338545	0.0447	0.242	15.1	---
10339915	0.0091	0.241	22.9	---
10339536	0.0011	0.240	3.7	---
10338684	0.0016	0.239	4	---
10607421	0.0104	0.239	17.9	*4930524N10Rik*

10344207	0.0163	0.239	13.6	---
10343286	0.0102	0.238	10.9	---
10341560	0.0331	0.238	18.4	---
10598081	0.0130	0.237	2.9	*mt-Ty*
10344546	0.0273	0.236	12.4	---
10340501	0.0137	0.236	1	---
10341727	0.0058	0.236	16.1	---
10340445	0.0060	0.236	15.9	---
10341616	0.0131	0.236	2.1	---
10342757	0.0160	0.236	3.1	---
10340747	0.0330	0.236	13.6	---
10339569	0.0264	0.236	16.6	---
10411506	0.0486	0.235	17.9	---
10341984	0.0059	0.235	15.2	---
10341031	0.0062	0.235	1	---
10340735	0.0366	0.235	17.6	---
10342929	0.0318	0.234	15.8	---
10338478	0.0099	0.234	2.7	---
10547386	0.0008	0.234	15	*Adipor2*
10340333	0.0272	0.233	7.4	---
10340544	0.0430	0.233	13.7	---
10339725	0.0202	0.232	14.3	---
10340632	0.0348	0.232	6.2	---
10342121	0.0362	0.232	15.9	---
10339460	0.0207	0.231	3.4	---
10341478	0.0049	0.231	12.4	---
10342692	0.0012	0.231	15	---
10341757	0.0466	0.231	14.7	---
10343654	0.0493	0.231	15.8	---
10343846	0.0469	0.231	23.3	---
10339782	0.0361	0.231	5.8	---
10342466	0.0285	0.231	6	---
10338765	0.0472	0.230	14.6	---
10342483	0.0316	0.230	3.8	---
10532628	0.0149	0.230	2.1	*Myo18b*
10340053	0.0059	0.230	1.1	---
10341658	0.0200	0.229	1.6	---
10338550	0.0159	0.229	15.7	---
10550025	0.0104	0.228	17.8	*Gm6946*
10339825	0.0164	0.227	10.5	---
10341632	0.0142	0.226	18.5	---
10417253	0.0283	0.225	1	
10417281	0.0283	0.225	1	
10343130	0.0355	0.225	17.9	---
10343084	0.0195	0.225	11.5	---
10338547	0.0009	0.224	2.5	---
10338101	0.0488	0.224	16.5	---
10340202	0.0026	0.223	16.5	---

10340467	0.0099	0.223	11.2	---
10343915	0.0326	0.222	16.4	---
10340635	0.0150	0.222	2.7	---
10342760	0.0310	0.222	1.8	---
10608382	0.0381	0.222	1.2	*Srsy*
10343810	0.0273	0.222	13.1	---
10344579	0.0132	0.222	14.5	---
10339799	0.0454	0.221	15	---
10417239	0.0226	0.221	1.1	*Gm3696*
10414661	0.0030	0.221	3	*Hk1*
10340979	0.0016	0.221	1.6	---
10338600	0.0145	0.221	12	---
10338659	0.0409	0.221	2.2	---
10341337	0.0058	0.220	18	---
10343742	0.0230	0.220	16.7	---
10341642	0.0324	0.220	9.3	---
10343485	0.0470	0.220	13.7	---
10341831	0.0057	0.220	1.7	---
10344167	0.0223	0.219	16.3	---
10338072	0.0033	0.219	12.4	---
10608693	0.0043	0.218	3.7	---
10341241	0.0218	0.218	2.2	---
10339894	0.0349	0.218	16.6	---
10340013	0.0295	0.217	4	---
10342321	0.0245	0.217	17.6	---
10339853	0.0116	0.217	2.3	---
10342758	0.0309	0.216	18.3	---
10342764	0.0204	0.216	14.2	---
10343749	0.0496	0.216	2.7	---
10343410	0.0328	0.216	2.1	---
10417504	0.0321	0.215	1.1	*Gm3696*
10608407	0.0214	0.215	1.6	*Gm21732*
10338622	0.0379	0.215	14	---
10399691	0.0002	0.215	3	*Id2*
10338610	0.0472	0.215	9.3	---
10608308	0.0393	0.215	1.5	*Gm21732*
10343674	0.0340	0.213	1.4	---
10342548	0.0063	0.213	3	---
10343052	0.0303	0.213	3.4	---
10338647	0.0320	0.213	14.1	---
10338754	0.0217	0.212	14.1	---
10578401	0.0236	0.212	14.8	*Zfp42*
10339258	0.0064	0.212	15.6	---
10342236	0.0381	0.212	19.8	---
10339780	0.0166	0.212	17.2	---
10344068	0.0111	0.212	14.9	---
10343186	0.0006	0.212	13.9	---
10343436	0.0026	0.212	16.5	---

10338775	0.0474	0.212	3.5	---
10564165	0.0342	0.212	1.4	*LOC102640399*
10535623	0.0342	0.212	2.9	*Gm4871*
10346069	0.0231	0.212	3.9	---
10341788	0.0312	0.212	12.8	---
10339756	0.0109	0.211	17.9	---
10343965	0.0479	0.211	2.6	---
10338156	0.0447	0.211	13.2	---
10339016	0.0107	0.211	18.5	---
10417415	0.0330	0.211	1.1	*Gm3696*
10417773	0.0391	0.211	1.1	*Gm3696*
10452535	0.0002	0.211	22.2	---
10342114	0.0385	0.210	13.7	---
10338789	0.0431	0.210	7	---
10343881	0.0480	0.210	23.6	---
10582882	0.0151	0.210	3.9	*Gm10722*
10340961	0.0145	0.210	13.1	---
10340986	0.0079	0.209	15.3	---
10564159	0.0428	0.209	1.5	*LOC102640399*
10417446	0.0306	0.209	1.3	*Gm3696*
10417452	0.0306	0.209	1.3	*Gm3696*
10519514	0.0245	0.209	2.7	*Gm15730*
10485800	0.0061	0.209	19.4	*Olfr1308*
10414943	0.0002	0.208	14.4	*Trav7n-5*
10338447	0.0139	0.208	14.6	---
10344004	0.0136	0.208	14.9	---
10339311	0.0485	0.207	19.7	---
10342987	0.0210	0.207	14.2	---
10341800	0.0402	0.207	13.8	---
10339420	0.0092	0.207	15.6	---
10598041	0.0109	0.207	2.6	*mt-Tk*
10338708	0.0256	0.207	12	---
10340884	0.0251	0.207	15.6	---
10340956	0.0281	0.206	2.6	---
10343800	0.0148	0.206	15.1	---
10338991	0.0030	0.206	15.9	---
10344295	0.0484	0.205	14.8	---
10341408	0.0193	0.205	24	---
10339898	0.0218	0.205	13.7	---
10550820	0.0117	0.204	17.1	*Vmn1r178*
10342310	0.0322	0.204	15.6	---
10467372	0.0080	0.204	14.2	*Cyp2c38*
10564169	0.0368	0.203	1.5	*LOC102640399*
10339199	0.0073	0.203	15.8	---
10412513	0.0330	0.203	1.1	*Gm5797*
10338511	0.0421	0.203	1	---
10338759	0.0478	0.203	14.9	---
10341966	0.0034	0.202	24	---

10338804	0.0106	0.202	3.1	---
10344551	0.0180	0.202	9.4	---
10338270	0.0245	0.201	22.6	---
10441339	0.0175	0.201	3.9	*A630089N07Rik*
10338630	0.0493	0.201	18.1	---
10340828	0.0071	0.201	16	---
10412549	0.0254	0.201	1	*Gm3558*
10412543	0.0392	0.201	1	*Gm3696*
10338903	0.0034	0.201	14.8	---
10341964	0.0451	0.201	22.8	---
10344133	0.0244	0.200	14.8	---
10342389	0.0132	0.200	13.9	---
10344444	0.0408	0.200	11	---
10340621	0.0402	0.200	11.5	---
10338562	0.0412	0.200	18.8	---
10362939	0.0057	0.200	17.1	*Gm4795*
10339549	0.0274	0.199	20	---
10343942	0.0338	0.199	11.9	---
10340648	0.0370	0.199	18.2	---
10343868	0.0278	0.199	17	---
10338627	0.0072	0.198	2.6	---
10339061	0.0345	0.198	16.1	---
10339694	0.0083	0.198	13.8	---
10340858	0.0222	0.198	1	---
10344109	0.0190	0.197	16.8	---
10496077	0.0007	0.197	16	*Etnppl*
10398408	0.0222	0.197	19.1	*Mir376b*
10582388	0.0484	0.197	13.5	*Gm26497*
10341332	0.0166	0.196	15.6	---
10608260	0.0321	0.196	1.4	*Gm21732*
10340954	0.0159	0.196	16.3	---
10342284	0.0189	0.196	13.3	---
10343549	0.0433	0.196	3.4	---
10338425	0.0111	0.195	16.8	---
10344590	0.0255	0.195	15.3	---
10485357	0.0095	0.195	3.9	*Gm10800*
10343765	0.0107	0.195	16.2	---
10417258	0.0488	0.195	1	*Gm3002*
10402390	0.0321	0.195	18.7	*Serpina1b*
10342023	0.0395	0.195	14.2	---
10417226	0.0199	0.195	1.3	
10340160	0.0481	0.194	7.2	---
10412520	0.0303	0.194	1.3	*Gm3696*
10446769	0.0202	0.194	14.6	*Gm23649*
10338882	0.0492	0.194	4.3	---
10459669	0.0059	0.194	3.4	*Rpl5*
10343321	0.0330	0.193	15.3	---
10343502	0.0193	0.193	15.3	---

10417319	0.0336	0.193	1	*Gm3558*
10608293	0.0469	0.193	1.6	*Gm21732*
10339284	0.0162	0.193	14.6	---
10590860	0.0057	0.193	15.9	*Arhgap42*
10338920	0.0309	0.193	2	---
10340992	0.0124	0.193	2	---
10339947	0.0023	0.193	3.8	---
10344401	0.0211	0.192	13.3	---
10435075	0.0058	0.192	9.5	*Tfrc*
10424555	0.0411	0.192	15.2	*Gm25987*
10607300	0.0023	0.192	15.1	*Gm10437*
10340169	0.0215	0.192	16	---
10445153	0.0067	0.191	14.9	*Olfr121*
10338929	0.0169	0.191	15.5	---
10341758	0.0092	0.191	15.9	---
10338415	0.0202	0.191	19.2	---
10545235	0.0479	0.191	16.6	*Igkv8-28*
10338949	0.0498	0.190	19.4	---
10340760	0.0029	0.190	3.7	---
10339796	0.0470	0.190	16.9	---
10341025	0.0354	0.190	15.9	---
10344097	0.0195	0.190	3.5	---
10339930	0.0347	0.189	14.3	---
10338741	0.0275	0.189	23.7	---
10343665	0.0030	0.189	13	---
10339241	0.0341	0.188	6.1	---
10343371	0.0361	0.188	14	---
10588545	0.0247	0.187	3.7	---
10339458	0.0274	0.187	12.7	---
10338941	0.0010	0.187	14.9	---
10448182	0.0088	0.187	2.2	*Mir703*
10338595	0.0219	0.186	3.7	---
10342143	0.0419	0.186	18.6	---
10386756	0.0121	0.186	1.5	---
10545079	0.0020	0.186	17.5	*A530053G22Rik*
10343783	0.0488	0.185	24	---
10344210	0.0344	0.185	1.9	---
10532281	0.0160	0.185	23.2	*Vmn2r14*
10608551	0.0357	0.185	1.7	*Gm21732*
10343201	0.0403	0.185	15.3	---
10354504	0.0365	0.185	24	*Gm5976*
10341212	0.0346	0.184	15.5	---
10343905	0.0290	0.184	1.2	---
10342074	0.0363	0.184	20.2	---
10339540	0.0053	0.184	1	---
10341093	0.0489	0.184	4	---
10418169	0.0280	0.183	1.3	*1700054O19Rik*
10340715	0.0207	0.183	14.6	---

10343745	0.0151	0.183	16.1	---
10429968	0.0077	0.183	1	---
10343048	0.0336	0.183	2.5	---
10456577	0.0467	0.183	1.4	---
10338583	0.0351	0.182	13.8	---
10467529	0.0006	0.182	7.5	*Opalin*
10368159	0.0069	0.182	24	*Slc35d3*
10342958	0.0210	0.182	6.8	---
10344492	0.0192	0.182	14.3	---
10390691	0.0000	0.182	9.2	*Nr1d1*
10338634	0.0091	0.182	17.2	---
10440617	0.0145	0.182	23.2	*Rpl31-ps4*
10341823	0.0062	0.182	16.1	---
10340224	0.0052	0.181	1.6	---
10373751	0.0005	0.181	15.3	*8430429K09Rik*
10338453	0.0147	0.181	2.8	---
10342203	0.0212	0.181	13	---
10353626	0.0145	0.181	1.8	*ATP6*
10339018	0.0045	0.180	14.8	---
10338284	0.0021	0.180	17.9	---
10343033	0.0168	0.180	14.9	---
10338294	0.0155	0.180	11.5	---
10357103	0.0131	0.179	15.3	*Cdh19*
10341079	0.0436	0.179	17.1	---
10417286	0.0299	0.178	1.2	*Gm3696*
10338831	0.0292	0.178	12.2	---
10605355	0.0457	0.178	6.3	*Gm22351*
10564137	0.0493	0.177	1.9	*Snord115*
10338210	0.0090	0.177	15.8	---
10343994	0.0377	0.177	3.4	---
10440019	0.0009	0.177	24	*Tmem45a*
10598018	0.0072	0.177	2.8	*mt-Tf*
10521811	0.0302	0.177	3.6	*Gm10025*
10339655	0.0099	0.177	7.3	---
10417371	0.0070	0.177	1	*Gm3696*
10342174	0.0264	0.177	18.2	---
10582899	0.0334	0.177	3.3	*Gm10717*
10417264	0.0448	0.177	1.3	
10403990	0.0205	0.176	16.7	*Vmn1r197*
10510194	0.0315	0.176	1.8	*Hmgb1*
10510212	0.0315	0.176	1.8	*Hmgb1*
10598075	0.0144	0.176	3.2	*mt-Ta*
10410345	0.0027	0.176	10.9	*Zfp748*
10351616	0.0282	0.176	10.8	*Usf1*
10442125	0.0112	0.176	18.4	*Vmn2r99*
10523357	0.0247	0.176	2.8	*Gm25937*
10358454	0.0004	0.176	11	*Rbm3*
10341986	0.0395	0.175	4.7	---

10412503	0.0451	0.175	1.1	*4930555G01Rik*
10413229	0.0083	0.175	24	*Anxa11*
10338735	0.0202	0.175	15.4	---
10344484	0.0439	0.175	1.7	---
10359582	0.0016	0.175	14.3	*Fmo2*
10340263	0.0401	0.174	14.5	---
10338566	0.0500	0.174	1.6	---
10598049	0.0038	0.174	2.2	*ND3*
10542021	0.0221	0.174	17.8	---
10338605	0.0214	0.174	15.9	---
10338391	0.0248	0.173	16.7	---
10484844	0.0297	0.173	14.4	*Olfr1251*
10344248	0.0226	0.173	21.1	---
10341988	0.0173	0.173	2.9	---
10595165	0.0044	0.173	18	*Gm8087*
10341613	0.0027	0.173	18.4	---
10343826	0.0414	0.172	1.5	---
10542894	0.0343	0.172	4.5	---
10556611	0.0251	0.172	1.7	*Gm25683*
10419160	0.0094	0.172	12.5	*Gm8194*
10341122	0.0342	0.172	14.6	---
10344317	0.0206	0.172	12.5	---
10412537	0.0489	0.172	1	
10583312	0.0015	0.172	13.1	*Taf1d*
10344349	0.0012	0.171	14.9	---
10398396	0.0322	0.171	2.7	*Mir679*
10338662	0.0216	0.171	11.6	---
10608438	0.0432	0.171	1.3	*Gm21732*
10417245	0.0435	0.171	1.1	*Gm3558*
10450904	0.0232	0.171	2.9	*Scoc*
10339027	0.0059	0.171	15.2	---
10397536	0.0401	0.171	17.2	---
10340124	0.0378	0.171	4	---
10340487	0.0317	0.170	23.6	---
10499950	0.0184	0.170	15	*Gm22577*
10342534	0.0076	0.170	15.4	---
10340164	0.0179	0.170	1	---
10344454	0.0192	0.170	13.1	---
10562576	0.0055	0.170	15.5	*Plekhf1*
10342898	0.0290	0.170	16.6	---
10339063	0.0477	0.169	13.8	---
10339760	0.0060	0.169	14.8	---
10390084	0.0007	0.169	16.6	*A430060F13Rik*
10341481	0.0246	0.169	18	---
10510505	0.0338	0.168	2.7	*n-R5s193*
10338330	0.0438	0.168	22.7	---
10414706	0.0427	0.168	5.1	*Trav3-1*
10462091	0.0000	0.168	12.5	*Klf9*

10344189	0.0113	0.167	1.2	---
10598064	0.0137	0.167	3.1	*mt-Tl2*
10340203	0.0004	0.167	4	---
10343596	0.0350	0.167	9.6	---
10408485	0.0047	0.167	19.8	*Gm23206*
10340681	0.0028	0.167	3	---
10349147	0.0312	0.167	16.8	*Serpinb7*
10338740	0.0451	0.167	4.4	---
10339843	0.0459	0.167	14.8	---
10454129	0.0379	0.167	14.5	*Dsg1b*
10608615	0.0200	0.166	1.6	*Sly*
10556113	0.0003	0.166	11.6	*Rbm3*
10496862	0.0045	0.166	15.1	*LOC102632402*
10343897	0.0070	0.166	2	---
10372171	0.0016	0.166	16.6	---
10339833	0.0002	0.166	14.6	---
10373620	0.0042	0.166	18.9	*Olfr772*
10344495	0.0105	0.165	15.8	---
10563915	0.0381	0.165	1.5	*LOC102639749*
10563929	0.0381	0.165	1.5	*LOC102639749*
10339121	0.0243	0.165	15.2	---
10475262	0.0334	0.165	17.2	*Gm22865*
10342467	0.0273	0.165	11.3	---
10342054	0.0492	0.164	9.5	---
10341693	0.0302	0.164	14.6	---
10435501	0.0107	0.164	1.5	---
10358551	0.0073	0.164	15.3	*Hmcn1*
10339497	0.0121	0.164	9.2	---
10506296	0.0492	0.164	16.8	---
10439887	0.0206	0.164	2	---
10343419	0.0498	0.164	20.4	---
10564287	0.0252	0.164	15.7	---
10570604	0.0226	0.163	3.7	---
10457020	0.0154	0.163	3.3	*Gm17383*
10338463	0.0190	0.163	24	---
10563913	0.0320	0.163	1.7	*LOC102639749*
10563917	0.0320	0.163	1.7	*LOC102639749*
10563923	0.0320	0.163	1.7	*LOC102639749*
10342832	0.0394	0.163	15.7	---
10343844	0.0282	0.163	21.6	---
10339893	0.0162	0.163	15.7	---
10376408	0.0304	0.163	3.9	*Olfr316*
10395869	0.0060	0.163	11.8	*4921506M07Rik*
10341786	0.0154	0.162	1.7	---
10342777	0.0214	0.162	16	---
10343750	0.0063	0.162	14.6	---
10358583	0.0033	0.162	5.4	*Hmcn1*
10516906	0.0145	0.162	10.1	*Snora73b*

10445022	0.0021	0.161	12.1	*H2-M10.5*
10405626	0.0056	0.161	2.7	*Rpl17*
10439660	0.0008	0.161	15.9	*Gm609*
10511444	0.0057	0.160	1.5	*Gm11810*
10338231	0.0029	0.160	12.2	---
10340668	0.0192	0.160	18.4	---
10340652	0.0222	0.160	14.5	---
10341976	0.0047	0.160	18.6	---
10344352	0.0173	0.160	12.1	---
10341229	0.0442	0.160	15.9	---
10338956	0.0275	0.160	15.7	---
10522784	0.0418	0.160	13.1	*Hmgn2*
10417373	0.0374	0.160	1.2	
10598062	0.0114	0.159	2.9	*mt-Th*
10338130	0.0445	0.159	15.2	---
10539979	0.0020	0.159	16.5	*Vmn1r-ps34*
10338923	0.0457	0.159	13.9	---
10554808	0.0005	0.159	14.3	*Fzd4*
10375935	0.0194	0.159	2.9	*Skp1a*
10343544	0.0198	0.159	2.7	---
10564005	0.0389	0.159	1.6	*LOC102639749*
10343418	0.0157	0.159	6	---
10585543	0.0079	0.159	20.7	*Gm7444*
10341266	0.0096	0.158	18.4	---
10339356	0.0488	0.158	8.5	---
10500181	0.0011	0.158	2.9	*Gm5070*
10343357	0.0202	0.158	3.5	---
10437940	0.0024	0.158	1.8	*Gm7731*
10551304	0.0327	0.157	14.6	*Cyp2t4*
10604240	0.0045	0.157	2.7	*Rpl17*
10342413	0.0238	0.157	15.8	---
10342974	0.0243	0.157	15.9	---
10437846	0.0056	0.157	1	*Ifitm7*
10494369	0.0131	0.157	12.4	*Sf3b4*
10351658	0.0267	0.157	15.8	*Cd48*
10347726	0.0156	0.157	1.6	*Gm5257*
10339270	0.0165	0.156	16.9	---
10453759	0.0013	0.156	18.6	*Gm10554*
10571655	0.0301	0.156	13.1	*Gm24669*
10342474	0.0358	0.156	2.1	---
10343051	0.0112	0.156	19.4	---
10523277	0.0298	0.156	3	*9330159N05Rik*
10388728	0.0438	0.156	1.5	*Gm11190*
10340492	0.0189	0.156	11.4	---
10338908	0.0007	0.156	4.2	---
10604557	0.0032	0.156	16.9	*1700080O16Rik*
10341022	0.0322	0.156	21.8	---
10338039	0.0046	0.155	13.9	---

10503963	0.0005	0.155	2.2	*Gm12384*
10338317	0.0173	0.155	3.1	---
10408932	0.0115	0.155	1.4	*Gm8513*
10395409	0.0007	0.155	14.9	*Meox2*
10473608	0.0457	0.155	2.5	*Olfr1193*
10341517	0.0447	0.154	17.7	---
10398124	0.0487	0.154	5.5	*Gskip*
10553092	0.0000	0.154	12.2	*Dbp*
10341129	0.0289	0.153	18.1	---
10397645	0.0118	0.153	1.4	*Gpr65*
10532267	0.0274	0.153	4.8	*Vmn2r9*
10342210	0.0106	0.152	3.7	---
10588201	0.0104	0.152	1.3	*n-R5s88*
10338486	0.0055	0.152	3.9	---
10339932	0.0238	0.152	14.7	---
10428398	0.0252	0.152	7.7	*Eif3e*
10343614	0.0296	0.152	22.7	---
10342577	0.0498	0.152	5.8	---
10340280	0.0323	0.152	3.3	---
10344053	0.0056	0.152	13.2	---
10342834	0.0030	0.152	13.6	---
10339220	0.0031	0.151	16.6	---
10340904	0.0436	0.151	9.4	---
10493896	0.0005	0.151	13.7	*Lce3b*
10402435	0.0198	0.151	12.6	*Serpina3c*
10499914	0.0356	0.150	14.8	*Lce1b*
10339967	0.0169	0.150	15.4	---
10343775	0.0500	0.150	10.9	---
10566188	0.0392	0.150	13.5	*Olfr573-ps1*
10343466	0.0151	0.150	19.6	---
10471586	0.0003	0.150	22.6	*Hspa5*
10563718	0.0477	0.150	19.9	---
10600712	0.0251	0.150	15.5	*Gm25006*
10607398	0.0121	0.150	1.4	*Gm7150*
10343851	0.0370	0.150	20.7	---
10340496	0.0215	0.150	16.4	---
10455752	0.0109	0.150	12.3	*Snx24*
10368700	0.0006	0.149	16.4	*G630090E17Rik*
10354581	0.0290	0.149	12.9	*Dnah7a*
10340534	0.0152	0.149	3.2	---
10576899	0.0023	0.149	23.8	*n-R5s93*
10557488	0.0004	0.148	1	*Tbx6*
10603469	0.0006	0.148	10.9	*Rbm3*
10342902	0.0318	0.148	3.2	---
10365492	0.0467	0.148	20	*Gm5174*
10523754	0.0022	0.148	13.5	*Gm24097*
10339568	0.0360	0.148	4.6	---
10528170	0.0340	0.147	2.7	*Cycs*

10362418	0.0003	0.147	15.4	*Trdn*
10573908	0.0006	0.147	13.5	*Gm24254*
10466932	0.0143	0.147	1.2	*Insl6*
10340370	0.0218	0.147	2.9	---
10584069	0.0192	0.147	16.7	---
10340279	0.0022	0.147	21.2	---
10343693	0.0194	0.147	23.4	---
10339225	0.0130	0.147	2	---
10398440	0.0013	0.146	15.1	*Mir369*
10339913	0.0084	0.146	15.9	---
10583388	0.0039	0.146	15.2	*Olfr836*
10339112	0.0055	0.146	19.1	---
10339327	0.0117	0.146	14.7	---
10511366	0.0072	0.145	5.2	*Gm11780*
10591188	0.0063	0.145	11.8	*Olfr843*
10574023	0.0006	0.145	13.5	*Mt2*
10363696	0.0136	0.145	14.6	*D630028G08Rik*
10563931	0.0275	0.145	2	*LOC102639749*
10460146	0.0154	0.145	23.3	---
10506498	0.0164	0.145	1.6	*Gm12727*
10503216	0.0043	0.144	15.2	*Chd7*
10338758	0.0325	0.144	14.4	---
10460149	0.0235	0.144	3.9	*LOC101056141*
10458130	0.0299	0.144	17.9	*4933408B17Rik*
10450197	0.0215	0.144	22.9	*Btnl5-ps*
10338548	0.0393	0.143	16.9	---
10400302	0.0219	0.143	19.9	*n-R5s58*
10602677	0.0477	0.143	18.7	*Gpr143*
10342276	0.0423	0.143	6.9	---
10339019	0.0231	0.143	4.3	---
10344517	0.0114	0.143	14.7	---
10340455	0.0197	0.143	13.3	---
10340384	0.0133	0.143	3.5	---
10351588	0.0282	0.143	1	*Pvrl4*
10476989	0.0179	0.143	16.7	*Gins1*
10427268	0.0433	0.143	24	*Hoxc13*
10591123	0.0237	0.142	13.4	*Fat3*
10429843	0.0095	0.142	15.4	*Parp10*
10553471	0.0084	0.142	1.8	*4933405O20Rik*
10499917	0.0119	0.142	16.5	*Lce1a2*
10343477	0.0295	0.142	2.4	---
10427679	0.0023	0.142	15.7	*Spef2*
10339751	0.0372	0.142	17.7	---
10403255	0.0164	0.142	13.8	*Gm16505*
10547752	0.0281	0.142	17.6	*C1s2*
10557058	0.0007	0.142	14.3	*Polr3e*
10456490	0.0303	0.142	11.3	*Cep192*
10414218	0.0264	0.142	1.5	*Gm5622*

10344024	0.0310	0.142	5.5	---
10569198	0.0378	0.142	24	*Gm10575*
10505249	0.0407	0.141	1.5	*Gm21286*
10342263	0.0284	0.141	14.1	---
10338847	0.0405	0.141	14.1	---
10462879	0.0056	0.141	2.1	*Gm23300*
10582275	0.0000	0.141	13.1	*Slc7a5*
10339787	0.0321	0.141	1.6	---
10339715	0.0425	0.141	1.1	---
10340221	0.0301	0.141	14.8	---
10454653	0.0014	0.141	2.9	---
10443108	0.0166	0.141	11.5	*Syngap1*
10346365	0.0132	0.140	20	*Sgol2*
10458938	0.0230	0.140	2	---
10584354	0.0071	0.140	13	*Olfr875*
10484793	0.0301	0.140	16.6	*Olfr1223*
10599084	0.0335	0.140	1.7	*Gm21637*
10384229	0.0027	0.140	1.4	---
10535747	0.0022	0.140	1.3	*Gm10858*
10501793	0.0065	0.140	15.4	*Gm23733*
10383883	0.0002	0.140	18.3	*Gm11961*
10598083	0.0359	0.139	3.2	*mt-Ts1*
10603833	0.0395	0.139	4	*Usmg5*
10408220	0.0019	0.139	14.3	---
10428308	0.0127	0.139	13.7	*G930009F23Rik*
10341227	0.0414	0.139	1.6	---
10484818	0.0329	0.139	5.1	*Olfr1238*
10608539	0.0234	0.138	1.3	*Sly*
10407420	0.0011	0.138	15	*Net1*
10339572	0.0148	0.138	3.2	---
10479743	0.0402	0.138	18	*Gm14496*
10343589	0.0212	0.138	1.4	---
10531980	0.0001	0.138	4.8	*Gbp9*
10416705	0.0325	0.138	22.9	---
10597511	0.0122	0.138	3.7	*Gm5921*
10424435	0.0078	0.138	15	*Gm25628*
10341895	0.0112	0.138	24	---
10340073	0.0471	0.138	16.3	---
10340022	0.0335	0.138	9.3	---
10338305	0.0458	0.138	10.8	---
10537296	0.0241	0.138	16.5	*Mir490*
10417421	0.0421	0.138	1.5	
10344076	0.0047	0.138	4.5	---
10564023	0.0498	0.138	1.7	*LOC102639749*
10483074	0.0430	0.138	1	*Gcg*
10573615	0.0108	0.137	11.6	*Orc6*
10364712	0.0000	0.137	9.5	*Cirbp*
10343299	0.0444	0.137	11.5	---

10573198	0.0470	0.137	22	*Dnajb1*
10594986	0.0030	0.137	3.1	*Rpl17*
10402604	0.0224	0.137	1.7	---
10379530	0.0142	0.137	14.3	*Ccl12*
10453692	0.0088	0.137	17.3	---
10462136	0.0337	0.137	2.9	*Cycs*
10606016	0.0308	0.137	13.9	*Il2rg*
10480468	0.0037	0.137	17	*Tbpl2*
10535340	0.0060	0.137	13.3	*B630006N21Rik*
10461705	0.0077	0.136	15.1	*Olfr1431*
10338814	0.0072	0.136	4.3	---
10342198	0.0283	0.136	11.1	---
10357001	0.0264	0.136	3.8	---
10593225	0.0002	0.136	14.9	*Zbtb16*
10342090	0.0231	0.136	23.7	---
10371400	0.0007	0.136	15.7	*Cry1*
10537880	0.0417	0.136	2.6	---
10583163	0.0370	0.136	15.7	*Trpc6*
10338730	0.0421	0.135	7.6	---
10403031	0.0070	0.135	14.9	*Ighv1-55*
10378214	0.0006	0.135	14.1	*Gm26121*
10571747	0.0347	0.135	18.5	*n-R5s97*
10591620	0.0049	0.135	13.6	*Dock6*
10439114	0.0129	0.135	16.5	*Iqcg*
10467083	0.0012	0.135	3.8	---
10363486	0.0149	0.135	5.8	*Lrrc20*
10338162	0.0021	0.135	1.9	---
10339184	0.0300	0.135	1.4	---
10580219	0.0000	0.135	24	*Calr*
10601753	0.0086	0.135	4.4	*Gm16410*
10473874	0.0296	0.135	13.3	*Arfgap2*
10531061	0.0118	0.135	16.7	*Ugt2b37*
10416835	0.0055	0.135	3.2	*Rpl17*
10570735	0.0358	0.135	14.5	*Defa24*
10578249	0.0429	0.135	1	*Gm10683*
10473505	0.0011	0.134	17.8	*Olfr1076*
10421930	0.0244	0.134	12.5	*Gm23509*
10406364	0.0243	0.134	12.8	*2210408I21Rik*
10342024	0.0019	0.134	15.2	---
10346328	0.0219	0.134	3.2	*Gm8292*
10598227	0.0353	0.134	1.7	*Gm21637*
10607332	0.0037	0.134	15.4	*Gm24460*
10491484	0.0004	0.133	14.6	*Gm25696*
10398410	0.0363	0.133	18.6	*Mir376a*
10407537	0.0229	0.133	16.1	*Idi2*
10607431	0.0150	0.133	18.4	*Magea6*
10552118	0.0462	0.133	2.7	*Gm12755*
10425046	0.0477	0.133	1	---

10537793	0.0437	0.133	1.1	*Olfr38*
10488439	0.0444	0.133	2.1	*Gm14140*
10389229	0.0007	0.133	2.2	*Rpl9*
10353475	0.0034	0.133	6.6	*Eif3m*
10568763	0.0058	0.133	15.2	*Gm25798*
10338631	0.0041	0.133	7.1	---
10338592	0.0306	0.133	13.5	---
10586865	0.0116	0.132	10.8	*Aldh1a2*
10440279	0.0177	0.132	3.8	*Csnka2ip*
10529741	0.0191	0.132	2.5	*Rab28*
10600326	0.0202	0.132	18.4	*Tktl1*
10439285	0.0390	0.132	2.5	*Gm6815*
10536743	0.0176	0.132	16.8	*6530409C15Rik*
10544629	0.0467	0.132	21.4	*Tra2a*
10603242	0.0429	0.132	1.8	*Gm21637*
10608263	0.0494	0.132	1.5	*Sly*
10500565	0.0069	0.132	15.5	*Hsd3b1*
10604232	0.0433	0.132	13.8	*Gm26437*
10456486	0.0019	0.132	15.8	---
10355199	0.0223	0.131	14.2	*Crygb*
10343975	0.0477	0.131	13.9	---
10513583	0.0275	0.131	10.6	*Cdc26*
10415608	0.0004	0.131	12.4	*Ift88*
10339869	0.0309	0.131	19.6	---
10589798	0.0407	0.131	1.5	---
10339443	0.0098	0.131	16.2	---
10484773	0.0125	0.131	14.7	*Olfr1214*
10347222	0.0478	0.131	3.1	*Gm24497*
10343489	0.0127	0.131	13.2	---
10400405	0.0062	0.131	12.6	*Nfkbia*
10341312	0.0362	0.131	15.5	---
10352267	0.0141	0.131	15.1	*Lin9*
10395978	0.0149	0.130	14	*Gm527*
10346191	0.0167	0.130	4	*Stat1*
10413136	0.0069	0.130	14	*Gm23502*
10338163	0.0333	0.130	15.7	---
10368679	0.0027	0.130	13.6	*Gm22911*
10463345	0.0243	0.130	1.3	*1700084K02Rik*
10416510	0.0081	0.130	8.8	*Nufip1*
10340473	0.0481	0.130	11.9	---
10455682	0.0020	0.130	13.4	*Fam170a*
10453443	0.0075	0.130	13.3	*1700011E24Rik*
10474377	0.0325	0.130	16.2	*Gm13915*
10445143	0.0041	0.130	15.4	*Olfr122*
10343928	0.0107	0.129	3.4	---
10360858	0.0268	0.129	1	*C130074G19Rik*
10417599	0.0031	0.129	21.7	---
10421524	0.0031	0.129	21.7	---

10436826	0.0031	0.129	21.7	---
10476658	0.0031	0.129	21.7	---
10579823	0.0031	0.129	21.7	---
10582845	0.0031	0.129	21.7	---
10404059	0.0020	0.129	7.3	*Hist1h1c*
10435048	0.0485	0.129	4.3	*Tctex1d2*
10571599	0.0039	0.129	15.7	---
10485674	0.0170	0.129	10.1	*Arl14ep*
10366546	0.0189	0.129	14.4	*Cpm*
10608654	0.0258	0.129	5	---
10395974	0.0173	0.129	16.7	*Gm24921*
10343824	0.0102	0.129	3.3	---
10458884	0.0031	0.129	24	*Hdhd1a*
10468399	0.0072	0.129	15.4	*Wdr96*
10494411	0.0083	0.128	15.2	*Rnu1b1*
10494417	0.0083	0.128	15.2	*Rnu1b1*
10500356	0.0083	0.128	15.2	*Rnu1b1*
10531645	0.0008	0.128	8.5	*Hnrnpdl*
10354919	0.0113	0.128	15.7	*LOC102636554*
10417492	0.0268	0.128	1	*Gm5796*
10399421	0.0014	0.128	23.8	*Mycn*
10488456	0.0454	0.128	19.4	*Gm14122*
10539702	0.0488	0.128	2.8	*Fam136a*
10421875	0.0106	0.128	1	*Gm23926*
10608226	0.0280	0.128	1.6	*Sly*
10604877	0.0269	0.128	16.6	*Mir465*
10476617	0.0296	0.127	15.1	*Gm14081*
10599826	0.0187	0.127	16.2	*F9*
10542034	0.0337	0.127	5.2	*Gm4968*
10450101	0.0430	0.127	12.8	*Mir219a-1*
10339208	0.0085	0.127	16.9	---
10545036	0.0008	0.127	17.3	*Vmn1r27*
10564089	0.0378	0.127	1.4	*Snord115*
10340368	0.0188	0.127	3.2	---
10564290	0.0017	0.127	16.5	*Klf13*
10357084	0.0261	0.127	18.7	*Serpinb3b*
10344214	0.0393	0.126	1.8	---
10344293	0.0000	0.126	15	---
10562761	0.0482	0.126	1	*Clec11a*
10344505	0.0042	0.126	10.4	---
10373452	0.0038	0.126	9.8	*Ciart*
10344107	0.0350	0.126	18.8	---
10357529	0.0002	0.126	13.3	*C4bp-ps1*
10578387	0.0119	0.126	14.2	*Zfp353-ps*
10443946	0.0357	0.126	21.9	*Actl9*
10541184	0.0436	0.126	3.4	---
10494413	0.0091	0.126	15	*Rnu1b1*
10494421	0.0091	0.126	15	*Rnu1b1*

10500343	0.0091	0.126	15	*Rnu1b1*
10500358	0.0091	0.126	15	*Rnu1b1*
10512937	0.0091	0.126	15	*Rnu1b1*
10497199	0.0190	0.126	3.4	*Tmem189*
10552242	0.0069	0.126	3.3	*Rpl17*
10434229	0.0015	0.126	23.5	*Cldn5*
10541632	0.0302	0.126	16.1	*Vmn2r23*
10344160	0.0463	0.125	11.8	---
10338070	0.0284	0.125	22.2	---
10505914	0.0238	0.125	15.2	*Zfp352*
10346109	0.0389	0.125	9.8	*Dnah7b*
10366705	0.0010	0.125	2.8	*Gm9081*
10338213	0.0032	0.125	13.5	---
10379511	0.0224	0.125	1.8	*Ccl2*
10338916	0.0465	0.125	15.6	---
10581605	0.0410	0.125	12.8	*Hp*
10509277	0.0074	0.125	1	*1700013G24Rik*
10374840	0.0460	0.125	3	---
10531972	0.0285	0.125	14.5	*Gbp8*
10560292	0.0076	0.125	13.4	*Gm24708*
10381111	0.0031	0.124	13.7	*Gm11560*
10510445	0.0096	0.124	14.8	---
10514201	0.0462	0.124	11.7	*Haus6*
10605081	0.0038	0.124	5.7	*Bcap31*
10545121	0.0211	0.124	13.9	*Vmn1r36*
10559177	0.0244	0.124	18.4	*B130016D09Rik*
10526452	0.0169	0.124	15.4	*Upk3bl*
10600825	0.0100	0.124	13.7	*Zc3h12b*
10339233	0.0114	0.124	16.7	---
10405563	0.0023	0.124	4	*Gm10782*
10356999	0.0055	0.124	3.7	*Prdx2*
10550730	0.0393	0.124	1.2	*Zfp296*
10504630	0.0214	0.124	17.7	*E230008N13Rik*
10442030	0.0171	0.124	15.3	---
10345875	0.0345	0.124	4.2	---
10456488	0.0424	0.124	13.8	*Cep192*
10396161	0.0066	0.124	10.8	*Tmx1*
10564027	0.0482	0.124	1.5	*Snord115*
10565353	0.0061	0.124	19.3	*Vmn2r68*
10445158	0.0074	0.124	14.9	*Olfr124*
10339382	0.0293	0.123	3.8	---
10559509	0.0296	0.123	20.9	*Cdc42ep5*
10579913	0.0497	0.123	2.2	*4930505O20Rik*
10343437	0.0086	0.123	18.3	---
10539981	0.0165	0.123	19.5	*Vmn1r54*
10342890	0.0182	0.123	4.3	---
10577343	0.0009	0.123	15.9	*Defb38*
10604587	0.0036	0.123	17.1	*Mir363*

10338409	0.0076	0.123	18.2	---
10591169	0.0343	0.123	16.5	*Muc16*
10484846	0.0357	0.123	19.5	*Olfr1252*
10483803	0.0113	0.123	14.7	*Haglr*
10539963	0.0230	0.122	22.3	*Vmn1r46*
10574143	0.0440	0.122	14.3	*Nlrc5*
10429772	0.0431	0.122	1.5	*Eppk1*
10468889	0.0201	0.122	14.6	*Gm22365*
10542104	0.0110	0.122	2.3	*Gm10069*
10565401	0.0328	0.122	15.9	*Folh1*
10403462	0.0374	0.122	1.4	*Dip2c*
10364518	0.0443	0.122	1.6	*Ptbp1*
10457243	0.0030	0.122	13.6	*Gm10125*
10480421	0.0458	0.122	16.8	*A130006I12Rik*
10551347	0.0013	0.122	15.4	*Blvrb*
10572635	0.0384	0.122	15.7	*Sfn*
10531259	0.0036	0.122	15.2	*Gm10426*
10427653	0.0050	0.122	18	*Spef2*
10560354	0.0364	0.122	15.3	---
10539802	0.0182	0.122	19.5	*Bmp10*
10433177	0.0011	0.122	21.5	---
10439080	0.0288	0.122	14.3	---
10388994	0.0018	0.121	13.5	*Gm10386*
10599346	0.0093	0.121	1.8	*LOC100862456*
10359297	0.0461	0.121	3.1	*Atp5h*
10406937	0.0179	0.121	18.3	*n-R5s56*
10338918	0.0439	0.121	1.2	---
10390798	0.0293	0.121	24	*Krt25*
10579341	0.0001	0.121	11.2	*Mpv17l2*
10351873	0.0174	0.121	18.1	*Pyhin1*
10601541	0.0130	0.121	14.1	*Cpxcr1*
10549633	0.0494	0.121	1.2	*Cdc42ep5*
10473481	0.0491	0.121	15.8	*Olfr1028*
10351667	0.0003	0.121	19.5	*Slamf1*
10344001	0.0301	0.121	15.6	---
10338020	0.0032	0.121	13.5	---
10372595	0.0021	0.121	20.1	*Gm10271*
10507961	0.0121	0.121	15.2	*Mtf1*
10519048	0.0299	0.121	19.9	*Actrt2*
10601537	0.0242	0.121	1.8	*Gm5943*
10598757	0.0201	0.121	17.6	*Gpr82*
10400163	0.0044	0.120	17.2	---
10603894	0.0409	0.120	16	*Gm14562*
10443980	0.0229	0.120	24	*Myo1f*
10549532	0.0325	0.120	16.1	*Gm23462*
10437132	0.0075	0.120	17.7	*Gm23692*
10466139	0.0016	0.120	14.8	*1700017D01Rik*
10462501	0.0108	0.120	15.9	*2700046G09Rik*

10483561	0.0095	0.120	16.2	*Gm26558*
10540472	0.0044	0.120	9.1	*Bhlhe40*
10530421	0.0220	0.120	14.4	*Gabra4*
10420889	0.0039	0.120	22.8	*1110020C17Rik*
10419158	0.0325	0.120	1.7	*Ang6*
10548779	0.0443	0.120	3	*Pbp2*
10459655	0.0259	0.120	13.8	*Poli*
10581031	0.0358	0.120	1.7	*LOC101056200*
10585379	0.0329	0.120	3.3	---
10557528	0.0011	0.120	13.6	*Kctd13*
10339139	0.0176	0.120	9.4	---
10339151	0.0057	0.120	6	---
10484837	0.0045	0.119	14.6	*Olfr1248*
10382852	0.0045	0.119	12.1	*Mfsd11*
10403248	0.0461	0.119	16.3	*Speer6-ps1*
10340523	0.0094	0.119	14.6	---
10340259	0.0228	0.119	16.1	---
10478289	0.0078	0.119	16.5	*Gm11452*
10456001	0.0094	0.119	8.6	*Rps14*
10548430	0.0152	0.119	12	*Gm156*
10586456	0.0176	0.119	14.9	*Gm23248*
10398751	0.0023	0.119	15.6	*Zfyve21*
10599411	0.0027	0.119	15.4	*Sh2d1a*
10355105	0.0079	0.119	4.4	---
10338029	0.0050	0.119	1	---
10400975	0.0402	0.119	23.9	*Trmt5*
10526101	0.0023	0.119	1.8	*Nupr1l*
10463252	0.0105	0.119	17.2	*Gm26339*
10381298	0.0006	0.118	13.4	*Ramp2*
10490838	0.0075	0.118	6.2	*Fabp5*
10468229	0.0482	0.118	2.2	*Gm9798*
10449284	0.0296	0.118	17.1	*Dusp1*
10573626	0.0009	0.118	13.9	*Gpt2*
10442115	0.0452	0.118	14	*Vmn2r96*
10517371	0.0481	0.118	14.1	*Gm12990*
10349100	0.0371	0.118	1.8	---
10603262	0.0061	0.118	2	*AU022751*
10433185	0.0208	0.118	15.7	*Spt1*
10433003	0.0076	0.118	2.4	*Sp7*
10338009	0.0213	0.118	13.1	---
10484612	0.0091	0.118	17.4	*Olfr1079*
10341083	0.0219	0.118	12.2	---
10437775	0.0451	0.118	2.1	*2310015D24Rik*
10339384	0.0477	0.118	12.4	---
10417998	0.0038	0.118	1	---
10605055	0.0330	0.118	13.2	*Haus7*
10604503	0.0373	0.118	4.9	*Olfr1324*
10348866	0.0085	0.117	1.7	*Atg4b*

10540296	0.0049	0.117	16.7	---
10595664	0.0234	0.117	9.7	*Tmed3*
10358188	0.0313	0.117	1.2	*Gpr25*
10461214	0.0153	0.117	6.2	*Tmem223*
10457407	0.0179	0.117	2.3	*LOC102639220*
10403312	0.0498	0.117	13	*Akr1c19*
10587077	0.0373	0.117	13.4	*Gm16551*
10600884	0.0051	0.117	3.3	*Rpl17*
10604867	0.0192	0.117	15.8	*Mir471*
10507095	0.0175	0.117	17.5	*Skint11*
10519905	0.0300	0.117	19.1	*Gnat3*
10387723	0.0205	0.117	17.2	*2810408A11Rik*
10362149	0.0178	0.117	17.1	*Taar2*
10448390	0.0289	0.117	23.1	*Prss41*
10445941	0.0065	0.116	1.3	---
10415052	0.0028	0.116	1.7	*Mmp14*
10567428	0.0105	0.116	16.2	*Dnah3*
10484762	0.0071	0.116	1.6	*Olfr1200*
10515939	0.0174	0.116	17.2	*Foxo6*
10553965	0.0207	0.116	14.7	---
10344414	0.0483	0.116	7.1	---
10338436	0.0296	0.116	16.4	---
10582997	0.0365	0.116	13.4	*Casp4*
10602176	0.0208	0.116	2.8	*Gm7123*
10457382	0.0370	0.116	10.4	*Gjd4*
10380506	0.0273	0.116	16	*Tac4*
10367517	0.0212	0.116	15.2	*Olfr804*
10460582	0.0070	0.116	10.6	*AI837181*
10385477	0.0021	0.116	16.2	*Gm23039*
10341657	0.0184	0.116	23.5	---
10371288	0.0004	0.116	16.4	*Gm1553*
10600821	0.0230	0.116	3	*Rpl13-ps5*
10340299	0.0045	0.116	18.7	---
10419469	0.0012	0.116	12	*Rpph1*
10493879	0.0165	0.116	14.2	*Sprr2i*
10419368	0.0454	0.115	1.3	---
10524417	0.0196	0.115	6.2	*Iscu*
10338356	0.0452	0.115	24	---
10343169	0.0240	0.115	3.9	---
10472050	0.0282	0.115	12.4	*Tnfaip6*
10343578	0.0209	0.115	15.7	---
10607393	0.0155	0.115	16.4	*Gm26035*
10530269	0.0040	0.115	15.6	*Rbm47*
10605784	0.0209	0.115	1.2	*LOC102640468*
10478383	0.0083	0.115	1	*R3hdml*
10606880	0.0328	0.115	16.1	*Glra4*
10426240	0.0085	0.115	1.7	*Klhdc7b*
10581308	0.0139	0.115	6	*Myl6*

10494889	0.0304	0.115	21.5	*Dennd2c*
10367515	0.0049	0.115	1.2	---
10375559	0.0010	0.115	20.5	---
10397786	0.0388	0.115	20.9	*Kif4-ps*
10585699	0.0125	0.114	7.1	*Fabp5*
10343953	0.0068	0.114	2.5	---
10402283	0.0002	0.114	14.5	*Itpk1*
10465965	0.0184	0.114	24	*Ppp1r32*
10430297	0.0352	0.114	6.4	*Pvalb*
10469782	0.0267	0.114	18.8	*Gm13410*
10419604	0.0209	0.114	14.1	*Olfr221*
10498716	0.0046	0.114	15.7	---
10526410	0.0030	0.114	20	*Hspb1*
10524264	0.0049	0.114	14.5	*Gm22583*
10408477	0.0380	0.114	14.9	*E2f3*
10560356	0.0379	0.114	14.9	---
10338041	0.0138	0.114	24	---
10558400	0.0093	0.114	13.6	*Nps*
10544108	0.0231	0.114	14.1	*Klrg2*
10516024	0.0176	0.113	1	*Tmco2*
10435930	0.0116	0.113	16.6	*Cd200r2*
10599956	0.0375	0.113	14.7	*Gm16407*
10376412	0.0359	0.113	1.4	*Olfr314*
10483401	0.0059	0.113	13.3	*Spc25*
10410709	0.0291	0.113	10.7	*Rfesd*
10472514	0.0082	0.113	14.5	*Nostrin*
10527487	0.0471	0.113	16.6	*Cyp3a57*
10550487	0.0213	0.113	18.5	*Mill1*
10472916	0.0215	0.113	13.9	*Cdca7*
10464391	0.0427	0.113	17.1	*Emx2*
10403743	0.0009	0.113	17.9	*Inhba*
10342635	0.0478	0.113	7.3	---
10439018	0.0078	0.113	6.5	*0610012G03Rik*
10454099	0.0135	0.113	3	*Morf4l1*
10356353	0.0061	0.113	16.2	*Alppl2*
10562096	0.0053	0.113	7.4	*Tmem147*
10584433	0.0208	0.113	19.4	*Olfr914*
10402302	0.0001	0.113	13.9	*Btbd7*
10416107	0.0475	0.112	23.1	*Gm10032*
10367475	0.0194	0.112	4.1	---
10417766	0.0342	0.112	17.2	---
10403871	0.0069	0.112	18.2	*Aoah*
10350502	0.0085	0.112	8	*Gm5262*
10339731	0.0259	0.112	14.7	---
10481949	0.0405	0.112	17.6	*Traf1*
10350840	0.0033	0.112	14.9	*Angptl1*
10569278	0.0393	0.112	1.3	*Dusp8*
10363333	0.0255	0.112	1.2	*Sh3rf3*

10344835	0.0489	0.112	3.3	---
10411945	0.0203	0.112	23.2	*Fam159b*
10424743	0.0323	0.112	5.4	*Tigd5*
10427673	0.0485	0.112	16.5	*Spef2*
10568622	0.0017	0.112	3.2	---
10401956	0.0424	0.112	23	---
10366517	0.0348	0.112	3.5	*Kcnmb4os2*
10440467	0.0400	0.112	2	*LOC102640240*
10338706	0.0174	0.112	11	---
10546180	0.0053	0.112	22.4	*Gm839*
10347216	0.0346	0.112	2	*Gm12618*
10389261	0.0213	0.112	17	*Gm11437*
10584572	0.0018	0.111	1.3	*Hspa8*
10582190	0.0139	0.111	16.2	*Gins2*
10414940	0.0021	0.111	1	*Tcra-V22.1*
10388389	0.0203	0.111	1.5	*Hic1*
10338641	0.0390	0.111	19.1	---
10419162	0.0063	0.111	1.4	*4930503E14Rik*
10429389	0.0063	0.111	3.6	*Rpl17*
10447356	0.0263	0.111	11.3	*Socs5*
10527182	0.0323	0.111	11.1	*E130309D02Rik*
10391752	0.0229	0.111	14	*Psmb5-ps*
10408928	0.0057	0.111	20.3	*Hspb1*
10439874	0.0118	0.111	17.4	*Ccdc54*
10543407	0.0130	0.111	17.3	*Rnf148*
10462343	0.0001	0.111	14.1	*Gm9895*
10584271	0.0492	0.111	8.6	*Tmem218*
10556487	0.0262	0.111	2.8	---
10441794	0.0142	0.111	15.2	*Mrgprh*
10492584	0.0056	0.111	16.7	*Mir16-2*
10490244	0.0179	0.111	5	*Gm14410*
10490271	0.0179	0.111	5	*Gm14410*
10601980	0.0307	0.111	15.4	*Mum1l1*
10457400	0.0319	0.110	3.4	---
10403413	0.0228	0.110	3.4	*Idi1*
10525069	0.0219	0.110	11.5	*Lhx5*
10338986	0.0262	0.110	19.2	---
10519219	0.0229	0.110	13	*Gltpd1*
10434922	0.0016	0.110	17.5	---
10477894	0.0094	0.110	1.1	*Gm14168*
10480432	0.0043	0.110	14	*Mastl*
10562117	0.0343	0.110	1.6	*Ffar2*
10525173	0.0468	0.110	13.4	*Oas1h*
10341276	0.0307	0.110	8.5	---
10580765	0.0000	0.110	9.1	*Pllp*
10556553	0.0052	0.110	2	*Insc*
10480921	0.0140	0.110	16.1	*Qsox2*
10513737	0.0034	0.110	3.3	*Rpl17*

10342526	0.0319	0.110	5.8	---
10439409	0.0091	0.110	15.9	*BC031361*
10520043	0.0016	0.110	12.3	*Lhfpl3*
10414250	0.0211	0.110	2	*Gm2832*
10414987	0.0108	0.110	17	*Trdv1*
10426425	0.0454	0.110	20.3	*Pdzrn4*
10391061	0.0225	0.110	22.9	*Krt16*
10414271	0.0441	0.110	13.7	*Ptger2*
10545255	0.0195	0.110	12.7	*Rpia*
10447885	0.0361	0.110	15.2	*Acat3*
10383208	0.0294	0.110	17.2	*Rnf213*
10406939	0.0280	0.110	2.4	*Gm12618*
10357886	0.0438	0.110	15.6	*Gm24517*
10390886	0.0445	0.110	21.6	*Krtap3-3*
10351689	0.0416	0.110	1	*Gm10521*
10608697	0.0494	0.110	6.8	---
10450767	0.0159	0.110	16	*H2-M10.1*
10385500	0.0495	0.110	20.3	*Irgm1*
10566157	0.0096	0.110	15.5	*Olfr551*
10474339	0.0171	0.110	13.8	*BC048594*
10343741	0.0215	0.110	9.7	---
10385511	0.0239	0.110	12.8	*Psme2*
10576216	0.0195	0.109	16.8	*Snord68*
10392930	0.0459	0.109	3.9	*Atp5h*
10427689	0.0059	0.109	15.1	*Spef2*
10367495	0.0054	0.109	16.8	*Olfr786*
10404018	0.0188	0.109	16.8	*Vmn1r219*
10520796	0.0168	0.109	14.7	*4930548H24Rik*
10455210	0.0053	0.109	3.5	*Rpl17*
10526687	0.0207	0.109	11.8	*Ppp1r35*
10421970	0.0499	0.109	4.1	*Gm25831*
10420357	0.0189	0.109	17.3	*Gm22218*
10481682	0.0031	0.109	2.5	*Ttc16*
10567211	0.0244	0.109	3.7	---
10352344	0.0317	0.109	15.8	*9130409I23Rik*
10604606	0.0183	0.109	18.1	*Mir450-2*
10363054	0.0225	0.109	14.5	---
10367726	0.0317	0.109	4.7	*LOC102632778*
10539015	0.0470	0.109	7.3	---
10536141	0.0084	0.109	16.8	*Ctdnep1*
10414168	0.0132	0.109	23.5	*Lrit2*
10341185	0.0500	0.109	20.2	---
10576029	0.0105	0.109	5	*Cox4i1*
10542376	0.0428	0.109	4.9	*Rpl36al*
10402061	0.0040	0.108	15.9	*Eml5*
10342725	0.0035	0.108	15.9	---
10461728	0.0087	0.108	15.5	*Gm4952*
10440246	0.0401	0.108	12.6	*Arl13b*

10583436	0.0324	0.108	15.6	*Olfr869*
10466680	0.0293	0.108	11.5	---
10353048	0.0250	0.108	17.2	*Gm24674*
10535575	0.0325	0.108	1.1	*Gm6272*
10574598	0.0081	0.108	1	*Ces3a*
10601701	0.0265	0.108	7.5	*Tmem35*
10563745	0.0308	0.108	18.6	*Mrgprb5*
10344330	0.0496	0.108	2.5	---
10594480	0.0459	0.108	3.9	*Rab11a*
10404783	0.0368	0.108	20.7	*Edn1*
10406452	0.0088	0.108	15.2	*Gm10759*
10376094	0.0188	0.108	3.9	*Gm12222*
10339566	0.0451	0.108	14.5	---
10342530	0.0199	0.108	3.3	---
10455826	0.0031	0.108	1.4	*Megf10*
10340494	0.0164	0.108	17.1	---
10523717	0.0114	0.108	2.6	*Spp1*
10428299	0.0033	0.108	15.4	---
10576816	0.0431	0.108	1	*Cd209b*
10592850	0.0010	0.108	5.2	*Trappc4*
10468213	0.0186	0.108	14.8	---
10382339	0.0038	0.108	2.4	*Gm16487*
10593469	0.0357	0.107	15.8	*Mir34c*
10416533	0.0224	0.107	16.7	*Ccdc122*
10519940	0.0008	0.107	13.9	*Gm23570*
10440143	0.0132	0.107	16	*Olfr178*
10519475	0.0010	0.107	18.3	---
10396059	0.0042	0.107	3.6	*Rpl17*
10507594	0.0024	0.107	13.4	*Slc2a1*
10400570	0.0138	0.107	1.1	---
10342307	0.0085	0.107	4.1	---
10388996	0.0344	0.107	14.9	*Crlf3*
10555811	0.0485	0.107	12.1	*Olfr628*
10478590	0.0054	0.107	4.2	*Zswim1*
10354534	0.0117	0.107	15.6	*1700019A02Rik*
10338027	0.0161	0.107	13.3	---
10505000	0.0060	0.107	9.2	*Nipsnap3b*
10358543	0.0183	0.107	15.2	*Hmcn1*
10405938	0.0142	0.107	16.1	*Zfp273*
10590304	0.0241	0.107	22.7	---
10542520	0.0089	0.107	3.6	*Atp5h*
10384373	0.0228	0.107	15.4	*Fignl1*
10605811	0.0340	0.107	4	*Amer1*
10448262	0.0293	0.107	2.1	*Zfp213*
10403420	0.0080	0.107	16.6	*Idi2*
10451761	0.0081	0.107	14.1	*Tbc1d5*
10449547	0.0218	0.107	3.9	---
10454514	0.0110	0.107	2.7	*Lims2*

10536607	0.0098	0.106	15.5	*Gm22828*
10379627	0.0038	0.106	17.7	*Gm11424*
10519124	0.0017	0.106	1	*Gabrd*
10572861	0.0472	0.106	5	*F2rl3*
10342813	0.0466	0.106	10.5	---
10338049	0.0120	0.106	12.9	---
10574641	0.0317	0.106	2.3	*D230025D16Rik*
10412036	0.0498	0.106	5.4	*Apoo*
10399725	0.0428	0.106	13.8	*Sox11*
10454252	0.0197	0.106	12.7	---
10457181	0.0122	0.106	15.8	*Gm23859*
10473477	0.0270	0.106	23.3	*Olfr1025-ps1*
10340041	0.0371	0.105	4.3	---
10338036	0.0254	0.105	23.3	---
10585986	0.0147	0.105	12	*Myo9a*
10397984	0.0203	0.105	13.6	*Ppp4r4*
10344212	0.0120	0.105	9.9	---
10374929	0.0418	0.105	24	*Fem1a*
10511892	0.0013	0.105	15.7	*Gm25705*
10463799	0.0301	0.105	1	---
10460385	0.0293	0.105	1	*Clcf1*
10583207	0.0003	0.105	16.7	*Maml2*
10385747	0.0055	0.105	14.5	*Jade2*
10438592	0.0018	0.105	21.4	*Liph*
10578761	0.0000	0.105	16.5	---
10564563	0.0047	0.105	3.9	*Rpl17*
10448131	0.0136	0.105	16.6	*Vmn2r94*
10544315	0.0299	0.105	1	*Gm4744*
10498568	0.0055	0.105	15.7	*Shox2*
10567506	0.0299	0.105	16.6	*Dnah3*
10595622	0.0479	0.105	22.8	*LOC102632389*
10595636	0.0479	0.105	22.8	*LOC102632389*
10411711	0.0421	0.105	5.7	*Cdk7*
10536324	0.0402	0.104	1	*Asb4*
10383813	0.0420	0.104	24	*Mtfp1*
10591131	0.0148	0.104	17.7	*Fat3*
10520109	0.0473	0.104	15.6	---
10358595	0.0023	0.104	24	*Hmcn1*
10524353	0.0188	0.104	14.3	*Tfip11*
10566585	0.0136	0.104	20.1	*Gm1966*
10564448	0.0293	0.104	15	*Asb7*
10606204	0.0326	0.104	12.6	*Gm23378*
10527012	0.0407	0.104	12.4	*Lfng*
10416112	0.0432	0.104	4.3	*Pnma2*
10482172	0.0288	0.104	21.8	*Zbtb26*
10566601	0.0097	0.104	4.8	*Olfr700*
10371578	0.0331	0.104	13.6	*Ascl1*
10566603	0.0211	0.104	14.2	*Olfr702*

10543302	0.0090	0.104	14.3	---
10529344	0.0310	0.104	11.6	*Haus3*
10338435	0.0022	0.104	15.6	---
10358523	0.0435	0.104	24	*Hmcn1*
10562280	0.0096	0.104	22.7	*Scgb1b2*
10601459	0.0449	0.104	17.4	*Pou3f4*
10416120	0.0112	0.103	1.4	*4930578I07Rik*
10426891	0.0064	0.103	17.3	*Mettl7a1*
10450611	0.0239	0.103	9.1	*Ppp1r18os*
10490199	0.0235	0.103	3.3	*Mir296*
10411622	0.0088	0.103	17.4	*Naip7*
10504948	0.0369	0.103	16.7	*Cylc2*
10374777	0.0194	0.103	11.9	*Efemp1*
10381115	0.0190	0.103	11.3	*Eif1*
10381416	0.0071	0.103	5.9	*Rnd2*
10565216	0.0027	0.103	19.7	*Gm10610*
10422225	0.0091	0.103	12.6	*Gm24207*
10493816	0.0156	0.103	24	*S100a5*
10344046	0.0116	0.103	4.7	---
10389865	0.0116	0.103	5.4	*Nme1*
10406276	0.0216	0.103	1	*Gm24163*
10560408	0.0207	0.103	16.6	*Psg17*
10519052	0.0253	0.103	8.3	*Fam213b*
10495929	0.0286	0.103	15.7	*Mir302d*
10607712	0.0241	0.103	17.2	*Grpr*
10551401	0.0040	0.103	4	*Ttc9b*
10607467	0.0281	0.102	2.4	*Sat1*
10344811	0.0011	0.102	14	*Cspp1*
10606989	0.0066	0.102	13.6	*Tsc22d3*
10515974	0.0373	0.102	18.9	*Nfyc*
10422234	0.0465	0.102	23.3	*Gm4775*
10392803	0.0316	0.102	22.1	*Cd300c*
10604893	0.0424	0.102	18.7	*Gm4910*
10416379	0.0362	0.102	6.8	*Sucla2*
10555254	0.0169	0.102	1.5	*Mir326*
10484667	0.0074	0.102	4.4	*Gm13730*
10508465	0.0121	0.102	5.6	*Marcksl1*
10569291	0.0391	0.102	2.6	*Krtap5-2*
10604643	0.0011	0.102	13.3	*1700013H16Rik*
10345030	0.0011	0.102	6.2	*Mir133b*
10489038	0.0108	0.102	12.5	*Scand1*
10545030	0.0154	0.102	16.1	*Vmn1r25*
10484754	0.0450	0.102	2.2	*Olfr1197*
10605603	0.0252	0.102	19.5	*Mageb4*
10356510	0.0405	0.102	18.4	*Iqca*
10353727	0.0002	0.102	14.8	*Gm25814*
10597999	0.0317	0.102	14.2	---
10528478	0.0056	0.102	3.7	*Rpl17*

10536746	0.0422	0.102	1.7	*Arf5*
10340592	0.0196	0.102	1.5	---
10484695	0.0069	0.102	14.1	*Olfr1144-ps1*
10426533	0.0224	0.102	15.9	*Gm23248*
10554413	0.0334	0.102	12	*Ngrn*
10392410	0.0033	0.102	1.8	*Gm10838*
10600718	0.0427	0.102	1	*Gm4184*
10535381	0.0003	0.101	10.7	*Actb*
10605651	0.0374	0.101	1.9	*Gm6977*
10605855	0.0472	0.101	14.8	*Hsf3*
10570483	0.0027	0.101	16.8	*Arhgef10*
10375499	0.0283	0.101	13	*Snord96a*
10393404	0.0097	0.101	21.6	*LOC100505199*
10465804	0.0046	0.101	8.6	*Polr2g*
10570418	0.0059	0.101	14.3	*Upf3a*
10453006	0.0199	0.101	11.5	*Cebpz*
10575775	0.0008	0.101	11.4	*Cmip*
10603182	0.0047	0.101	17.3	*Arhgap6*
10358656	0.0363	0.101	5.2	*Hmcn1*
10579066	0.0033	0.101	1	*LOC102642711*
10507461	0.0002	0.101	18.7	*Best4-ps*
10421737	0.0387	0.101	1.8	*Tnfsf11*
10545024	0.0177	0.101	17.2	*Vmn1r22*
10499930	0.0341	0.101	16.7	*Lce1g*
10484512	0.0334	0.101	2	*Olfr992*
10572989	0.0304	0.101	14.2	*Slc10a7*
10553741	0.0431	0.101	16.7	---
10410150	0.0396	0.101	16.7	*Cntnap3*
10578916	0.0132	0.100	4.9	*Msmo1*
10520763	0.0383	0.100	18.5	*Gckr*
10338062	0.0156	0.100	13.4	---
10380670	0.0052	0.100	3.6	*Rpl17*
10504670	0.0207	0.100	19.8	*E230008N13Rik*
10393294	0.0494	0.100	21.4	*Qrich2*
10342262	0.0356	0.100	13	---
10484539	0.0316	0.100	3.7	*Olfr1006*
10581926	0.0030	0.100	16.8	*Adat1*
10374775	0.0070	0.100	12.9	*Mir217*
10341971	0.0232	0.100	5.4	---
10356018	0.0274	0.100	1.8	*Gm6189*
10422179	0.0022	0.100	13.2	*Pou4f1*
10339606	0.0281	0.100	15.1	---
10536376	0.0307	0.100	8.2	*Mios*
10369825	0.0166	0.100	15.9	---
10442771	0.0024	0.100	23.1	*Prss28*
10579422	0.0184	0.100	3.2	*Kcnn1*
10562667	0.0287	0.100	1.4	---
10571772	0.0125	0.099	13.6	*Gm22841*

10364109	0.0047	0.099	1.8	*Vpreb3*
10595209	0.0443	0.099	16.3	*Gm10635*
10445376	0.0199	0.099	2.5	*Gm12618*
10591139	0.0037	0.099	15.9	*Naalad2*
10476689	0.0128	0.099	21	*Zfp133-ps*
10340210	0.0380	0.099	11	---
10436777	0.0041	0.099	2.5	---
10369688	0.0499	0.099	13.4	*Tet1*
10593198	0.0061	0.099	15.1	*Nxpe2*
10447354	0.0284	0.099	2.9	*Tmx2*
10414741	0.0033	0.099	1	*Tcra-V22.1*
10500960	0.0308	0.099	12.2	*Ddx20*
10598085	0.0420	0.099	2.3	---
10587690	0.0149	0.099	23.9	*Bcl2a1b*
10462096	0.0122	0.099	1	*Gm21542*
10544525	0.0234	0.099	22.1	*Pdia4*
10340464	0.0421	0.098	7	---
10600604	0.0209	0.098	13.3	*Dmd*
10522819	0.0007	0.098	17.1	*Ugt2b35*
10548194	0.0428	0.098	23.9	*Fkbp4*
10509002	0.0144	0.098	21.4	*Rhd*
10344616	0.0301	0.098	16.9	*Gm26206*
10576881	0.0111	0.098	16	*Gm7461*
10364194	0.0269	0.098	14.4	*Lss*
10507572	0.0157	0.098	3.8	*Olfr1340*
10523279	0.0403	0.098	15.8	*Gm22915*
10589911	0.0160	0.098	24	---
10452879	0.0171	0.098	14.9	*Nlrc4*
10382228	0.0294	0.098	19.2	*Axin2*
10338483	0.0204	0.098	2.1	---
10565499	0.0089	0.098	13.9	*Gm5341*
10395845	0.0422	0.098	16.7	---
10436251	0.0116	0.098	20.6	*Gm24047*
10521090	0.0066	0.098	11.3	*Tacc3*
10343519	0.0244	0.098	14.3	---
10424211	0.0499	0.098	19	*Gm10370*
10597268	0.0464	0.098	15.7	*Tdgf1*
10605172	0.0141	0.098	1.3	*Naa10*
10593878	0.0374	0.098	2	*Snx33*
10599554	0.0188	0.098	11.2	*Rbmx2*
10465366	0.0212	0.098	11.5	*Zfpl1*
10341951	0.0150	0.098	9.2	---
10475544	0.0060	0.098	3.8	*Sema6d*
10340622	0.0430	0.098	13.7	---
10497485	0.0441	0.098	12.1	*Naaladl2*
10503257	0.0086	0.097	15.3	*Gm11829*
10583806	0.0128	0.097	2.8	---
10424097	0.0430	0.097	12.7	*Med30*

10411223	0.0030	0.097	12.8	*S100z*
10416269	0.0301	0.097	11.7	*Gm9174*
10338016	0.0043	0.097	13.6	---
10522788	0.0181	0.097	13.8	*Stap1*
10550807	0.0023	0.097	17.7	*Vmn1r170*
10467149	0.0064	0.097	17.2	*2010002M12Rik*
10358654	0.0036	0.097	4.1	*Hmcn1*
10554863	0.0162	0.097	7	*Sytl2*
10459455	0.0161	0.097	16.9	*Alpk2*
10555818	0.0096	0.097	1	*Gm15115*
10480490	0.0009	0.097	12.9	*Gm13416*
10359303	0.0455	0.097	18.4	*4930562F07Rik*
10499536	0.0035	0.097	2.6	*Efna1*
10422219	0.0460	0.097	2.4	---
10493640	0.0323	0.097	22.5	*Nup210l*
10471749	0.0093	0.097	17.5	*Olfr345*
10453732	0.0482	0.097	20.8	---
10559513	0.0130	0.097	23.7	*Cab39*
10461143	0.0320	0.097	1	*Chrm1*
10355259	0.0014	0.097	15.6	*Myl1*
10566516	0.0065	0.097	13.6	*Rrp8*
10567299	0.0126	0.097	4.6	*Itpripl2*
10389070	0.0239	0.097	13.3	*Gm24612*
10553197	0.0303	0.097	21.5	*Gm9860*
10603841	0.0152	0.097	15.5	*Gm26065*
10599612	0.0111	0.096	15.5	*Phf6*
10500218	0.0064	0.096	3.5	*Tars2*
10445983	0.0217	0.096	16.3	*Ccdc94*
10346098	0.0212	0.096	23.7	---
10346544	0.0208	0.096	4.8	*Ndufb3*
10408024	0.0037	0.096	22.5	*Pgbd1*
10604499	0.0342	0.096	21.8	*Olfr1323*
10342226	0.0017	0.096	15.8	---
10599822	0.0460	0.096	23.4	*4930550L24Rik*
10362454	0.0047	0.096	15	*Trdn*
10587880	0.0289	0.096	14.3	*Pcolce2*
10568962	0.0391	0.096	1.8	*Olfr532*
10475987	0.0086	0.096	3.1	*Setd3*
10344587	0.0386	0.096	2.5	---
10577446	0.0429	0.096	6.6	*Defb15*
10416256	0.0464	0.096	16.8	*Bin3*
10492682	0.0285	0.096	6.3	*Fam198b*
10416215	0.0398	0.096	22.2	*Loxl2*
10344952	0.0095	0.096	12.9	*Rdh10*
10413997	0.0234	0.096	22.2	*Gm626*
10512499	0.0257	0.096	10.3	*Tpm2*
10598872	0.0067	0.096	16.8	*Rbm10*
10538689	0.0395	0.096	18.6	---

10463803	0.0039	0.096	14.6	*Slk*
10436828	0.0186	0.096	5.9	*Olig1*
10433618	0.0446	0.095	12.8	*Shisa9*
10424543	0.0270	0.095	2.6	*Wisp1*
10338024	0.0025	0.095	14	---
10468485	0.0003	0.095	14.8	*Gm25644*
10604873	0.0163	0.095	17.3	*Mir465*
10604875	0.0163	0.095	17.3	*Mir465*
10453426	0.0250	0.095	3.2	*Atp6v1e2*
10591194	0.0046	0.095	18.9	*Olfr850*
10490923	0.0016	0.095	3.5	*Car2*
10478145	0.0000	0.095	12.4	*Ppp1r16b*
10588755	0.0074	0.095	14.4	*Camkv*
10396472	0.0429	0.095	11.7	*Dbpht2*
10365971	0.0093	0.095	8.8	*Btg1*
10398356	0.0067	0.095	18.1	*Gm24899*
10505788	0.0343	0.095	1.1	*Acer2*
10490535	0.0089	0.095	3.8	*Bhlhe23*
10416931	0.0089	0.095	12.3	*Slitrk5*
10428839	0.0364	0.095	17.6	*Ube2d4*
10444046	0.0125	0.095	3.3	*Cd320*
10384223	0.0304	0.095	17	*Igfbp3*
10547906	0.0086	0.095	13.6	*Lag3*
10603896	0.0340	0.095	9.3	*Klhl13*
10420986	0.0336	0.095	2.6	*Gm12618*
10507655	0.0044	0.095	16.8	*Gm24955*
10602896	0.0221	0.095	15.9	*Gpr64*
10454782	0.0246	0.095	14.3	*Egr1*
10351482	0.0151	0.095	20.2	*1700015E13Rik*
10423556	0.0069	0.095	7.8	*Cpq*
10591090	0.0007	0.095	21.1	*Fat3*
10403951	0.0297	0.095	14.4	*Hist1h2bp*
10520948	0.0245	0.095	3	*Plb1*
10509941	0.0413	0.095	2.7	*Rsg1*
10392297	0.0370	0.095	15.3	*Gm11708*
10587688	0.0414	0.095	24	*LOC102632389*
10595620	0.0414	0.095	24	*LOC102632389*
10369290	0.0025	0.095	14.7	*Ddit4*
10515431	0.0430	0.094	22.6	*Kif2c*
10420241	0.0395	0.094	17.9	*Cma2*
10375326	0.0002	0.094	14.6	*Pwwp2a*
10576437	0.0386	0.094	17.6	*Gm24459*
10458804	0.0423	0.094	15.1	---
10400639	0.0274	0.094	5.6	*Rpl36al*
10545135	0.0163	0.094	21.4	*Il12rb2*
10518453	0.0047	0.094	7.2	*Chchd2*
10508907	0.0278	0.094	14.9	---
10552433	0.0022	0.094	15.5	*Zfp658*

10394934	0.0090	0.094	13.6	*LOC102632483*
10354765	0.0178	0.094	16.8	*LOC101055993*
10357064	0.0038	0.094	11.7	*Vps4b*
10357363	0.0242	0.094	1.8	*Nckap5*
10555834	0.0370	0.094	23.3	*Olfr633*
10352393	0.0213	0.094	8.9	*Srp9*
10399463	0.0142	0.094	1.8	---
10569958	0.0356	0.094	15.5	*Ccl25*
10414731	0.0363	0.094	16.1	*Trav6d-4*
10463153	0.0230	0.094	3.7	*Morf4l1*
10424695	0.0253	0.094	3.2	*Gpihbp1*
10346808	0.0100	0.094	3.1	*Rpl17*
10567626	0.0255	0.094	16.4	*Gga2*
10515213	0.0404	0.094	2.5	*Dmbx1*
10595695	0.0363	0.094	12.9	*Zic1*
10366178	0.0053	0.094	16.3	*Gm25143*
10485280	0.0182	0.094	22.4	*Gm13889*
10493820	0.0447	0.094	12.6	*S100a6*
10445877	0.0476	0.094	15	*Gm16489*
10559724	0.0159	0.094	16.7	*Vmn1r56*
10576965	0.0436	0.094	4.4	*Rps16-ps3*
10450242	0.0458	0.094	24	*C4b*
10373768	0.0154	0.094	11.6	*Selm*
10494972	0.0011	0.093	18	*Bcl2l15*
10591196	0.0206	0.093	14.5	*Olfr853*
10347503	0.0061	0.093	11.3	*Cdk5r2*
10511789	0.0499	0.093	9.5	*Nkain3*
10409063	0.0353	0.093	2.7	*Mirlet7a-1*
10422007	0.0287	0.093	19.5	*Gm25130*
10562181	0.0061	0.093	21.8	*Lsr*
10355836	0.0154	0.093	4.2	*Resp18*
10354883	0.0211	0.093	24	*Als2cr12*
10582080	0.0302	0.093	15.1	*Gm24035*
10513332	0.0361	0.093	14	*AI481877*
10587776	0.0182	0.093	14.7	*Gm22717*
10582862	0.0050	0.093	1.7	*LOC102633858*
10346410	0.0378	0.093	16.5	*Aox3*
10604564	0.0210	0.093	13.1	*Gpc4*
10456709	0.0068	0.093	3.7	*Rpl17*
10417568	0.0064	0.093	17.5	*Oit1*
10551393	0.0240	0.093	3.4	*Akt2*
10604019	0.0198	0.093	8.7	*Gm2036*
10385918	0.0125	0.093	18.5	*Il3*
10490621	0.0159	0.093	24	*Srms*
10388749	0.0040	0.093	17.3	*Traf4*
10371220	0.0118	0.093	13.2	*Gna15*
10338018	0.0028	0.093	13.5	---
10486712	0.0227	0.093	19.5	*Zscan29*

10400896	0.0140	0.093	12.8	*L3hypdh*
10553833	0.0095	0.093	4	*Ndn*
10340866	0.0240	0.093	1.6	---
10344821	0.0439	0.093	15.3	*Cspp1*
10420254	0.0258	0.093	2.4	*Mcpt8*
10586907	0.0093	0.093	19.8	*Mns1*
10368484	0.0196	0.092	10.3	*Gm9996*
10530417	0.0218	0.092	14.8	*Cox7b2*
10447383	0.0181	0.092	12.5	*Epcam*
10455824	0.0491	0.092	20.3	*Gm10536*
10341141	0.0307	0.092	13	---
10406905	0.0275	0.092	17.3	*Ccdc125*
10385248	0.0143	0.092	16.4	*Hmmr*
10520362	0.0283	0.092	8.5	*Insig1*
10605522	0.0349	0.092	12.9	*Gm7173*
10363676	0.0482	0.092	16.3	*Ctnna3*
10461176	0.0082	0.092	16	*Stx5a*
10490946	0.0303	0.092	2.4	*Hsp90aa1*
10501963	0.0200	0.092	2.9	*Ugt8a*
10395129	0.0291	0.092	12.3	*Tmem18*
10522467	0.0094	0.092	13.8	*Rasl11b*
10513266	0.0035	0.092	6.8	*Olfr267*
10367292	0.0231	0.092	3.7	*Cs*
10436823	0.0042	0.092	2.8	*Olig2*
10531183	0.0441	0.092	11.8	*Adamts3*
10501218	0.0123	0.092	23.6	*Gstm3*
10523903	0.0373	0.092	21.8	*Gm9850*
10496813	0.0239	0.092	12.5	*Ctbs*
10424073	0.0053	0.092	14.9	*Gm23848*
10587892	0.0463	0.092	12.5	*Atr*
10401002	0.0459	0.092	2	*Gphb5*
10548450	0.0388	0.092	18.3	*Klra5*
10561351	0.0130	0.092	14.4	*Zfp850*
10482045	0.0028	0.092	16.3	*4930568D16Rik*
10497033	0.0306	0.091	1.3	*Lrriq3*
10353252	0.0008	0.091	13.6	*Sbspon*
10406736	0.0025	0.091	22.8	*F2rl2*
10493631	0.0000	0.091	17.1	*Mir190b*
10348889	0.0075	0.091	23.4	*D2hgdh*
10602729	0.0009	0.091	15.9	*Gm22359*
10564978	0.0442	0.091	14.7	*Blm*
10598571	0.0241	0.091	3.7	*4930402K13Rik*
10592289	0.0072	0.091	17.2	*Ccdc15*
10472047	0.0377	0.091	22.3	*Tas2r134*
10491678	0.0358	0.091	16.5	*Adad1*
10398240	0.0014	0.091	3.8	*Eml1*
10439878	0.0059	0.091	3.4	*Psmc1*
10543333	0.0095	0.091	14.4	*Aass*

10456579	0.0071	0.091	14.4	*Mex3c*
10474028	0.0158	0.091	12.4	*Pex16*
10340661	0.0346	0.091	24	---
10385426	0.0312	0.091	1.9	*Hmgb1*
10442719	0.0178	0.091	1	*Ccdc154*
10482496	0.0495	0.091	15.8	*Gm13487*
10567103	0.0257	0.091	4.1	*Rpl5*
10389627	0.0102	0.091	22.7	*Rad51c*
10504417	0.0456	0.091	3	*Hrct1*
10545921	0.0371	0.091	15.3	*Mxd1*
10520268	0.0244	0.091	3.7	*1500035N22Rik*
10342883	0.0040	0.091	15.6	---
10595171	0.0142	0.091	16.6	*Mb21d1*
10374704	0.0108	0.091	17.6	*1700030C12Rik*
10385583	0.0212	0.091	16.6	*Ltc4s*
10401511	0.0163	0.090	3	*Syndig1l*
10601539	0.0234	0.090	14.4	*Gm24624*
10587501	0.0359	0.090	4	*Rps27a*
10386005	0.0486	0.090	3.9	*Atp5f1*
10576335	0.0089	0.090	4.6	*Def8*
10496023	0.0217	0.090	22.4	*Casp6*
10385151	0.0313	0.090	16.8	---
10403508	0.0194	0.090	2.8	*Gm12618*
10530832	0.0409	0.090	9.5	*Noa1*
10548504	0.0118	0.090	16.7	*Klra8*
10348451	0.0125	0.090	2	*Ackr3*
10447725	0.0222	0.090	10.5	*LOC101055707*
10405343	0.0279	0.090	10.8	*Tspan17*
10364744	0.0047	0.090	12.2	*Ndufs7*
10356403	0.0270	0.090	15.4	*Kcnj13*
10585085	0.0316	0.090	17.5	---
10439695	0.0403	0.090	6.1	*Tagln3*
10484920	0.0391	0.090	9	*Ptpmt1*
10409190	0.0045	0.090	16.3	*Cenpp*
10543895	0.0061	0.090	12.8	*2010107G12Rik*
10368888	0.0068	0.090	14.7	*Foxo3*
10498599	0.0240	0.090	16.1	*Ift80*
10598827	0.0228	0.090	17.4	---
10417734	0.0009	0.090	12.5	*Nr1d2*
10484431	0.0437	0.090	3.5	*Tmx2*
10342782	0.0277	0.090	1	---
10532680	0.0240	0.090	1.1	*Sgsm1*
10393936	0.0369	0.090	14	*Cbr2*
10528021	0.0051	0.090	1	*Hspa8*
10403727	0.0010	0.090	14.8	*Gli3*
10344244	0.0257	0.090	9.2	---
10468869	0.0417	0.090	4.9	*Prdx3*
10339221	0.0259	0.090	4.2	---

10607865	0.0148	0.090	5	*Tmsb4x*
10402473	0.0003	0.090	14.3	*Clmn*
10535310	0.0240	0.090	2.5	---
10477581	0.0097	0.089	4	*Rpl5*
10370013	0.0427	0.089	13.6	*Gstt2*
10363170	0.0106	0.089	2.1	*Msl3l2*
10377308	0.0315	0.089	4.5	*Mfsd6l*
10378855	0.0315	0.089	17.7	*Ssh2*
10440916	0.0125	0.089	16	---
10604889	0.0167	0.089	17.6	*Mir201*
10454873	0.0446	0.089	21.9	*4930471G03Rik*
10501555	0.0484	0.089	12.4	*Amy1*
10428441	0.0300	0.089	7.9	*Gm5471*
10572745	0.0352	0.089	24	*Olfr374*
10433691	0.0262	0.089	5.5	*Ntan1*
10516658	0.0379	0.089	15.4	*Ccdc28b*
10420011	0.0131	0.089	6	*Myl6*
10601416	0.0433	0.089	15.1	*P2ry10*
10546604	0.0316	0.089	17.8	*Gm26175*
10410721	0.0122	0.089	16	*Arsk*
10540248	0.0486	0.089	14.9	*Mitf*
10462922	0.0227	0.089	16.9	*Plce1*
10519117	0.0114	0.089	13.2	*Prkcz*
10487675	0.0024	0.089	15.2	*4930473A02Rik*
10591423	0.0307	0.089	6.6	*Raver1-fdx1l*
10362162	0.0017	0.089	18.5	*Taar7d*
10479996	0.0265	0.089	4.4	*Atp5c1*
10383423	0.0393	0.089	9.5	*Anapc11*
10581865	0.0025	0.089	22.6	*Ldhd*
10353624	0.0496	0.089	2.9	*ND4L*
10562632	0.0004	0.089	17.5	*Vmn2r-ps57*
10566403	0.0161	0.089	23.9	*Olfr668*
10567576	0.0360	0.089	14.8	*Gm9905*
10550250	0.0358	0.089	4.1	*Kptn*
10590844	0.0106	0.089	14.3	*Arhgap42*
10542050	0.0197	0.089	3.8	*Efcab4b*
10365482	0.0083	0.089	12.6	*Timp3*
10338055	0.0066	0.089	13.9	---
10458714	0.0147	0.088	18.4	*Gm10267*
10503399	0.0330	0.088	6	*Myl6*
10451083	0.0152	0.088	19.7	*Cdc5l*
10598994	0.0110	0.088	3.7	*Atp1b3*
10371877	0.0147	0.088	4.2	*Slc25a3*
10516778	0.0174	0.088	5.1	*Zcchc17*
10338042	0.0310	0.088	1.9	---
10470788	0.0186	0.088	13.3	*Odf2*
10427496	0.0010	0.088	17.1	*Egflam*
10506638	0.0370	0.088	5.8	*Cdcp2*

10542892	0.0341	0.088	12.1	*Nacc1*
10566358	0.0163	0.088	15.1	*Trim30a*
10543369	0.0186	0.088	11.1	*Cadps2*
10552140	0.0307	0.088	23.2	*Cebpa*
10455866	0.0146	0.088	14.1	*1700011I03Rik*
10530854	0.0209	0.088	14.7	*Tecrl*
10403333	0.0456	0.088	15.5	*Akr1c21*
10572800	0.0243	0.088	13.1	*Klf2*
10495094	0.0158	0.088	3.6	*Fam212b*
10428405	0.0326	0.088	15.7	*Gm10373*
10495945	0.0426	0.088	15.1	*Zgrf1*
10598769	0.0280	0.088	15.2	*Csnk2a1-ps*
10494460	0.0009	0.088	11.1	*Pex11b*
10578557	0.0282	0.088	11.5	*Primpol*
10550967	0.0368	0.088	19.1	*Phldb3*
10572038	0.0212	0.088	11.3	*Tmem192*
10551287	0.0153	0.088	13.4	*Cyp2a12*
10584710	0.0014	0.088	8.7	*H2afx*
10522494	0.0054	0.088	17	*Gm6116*
10532534	0.0491	0.088	3	*Gm6583*
10439296	0.0293	0.088	15.3	*Stfa2*
10453634	0.0148	0.088	15.4	*Gm10556*
10396862	0.0487	0.088	23.1	*Strm*
10435784	0.0465	0.088	5.3	*Ndufs5*
10570321	0.0014	0.088	11.4	*Cul4a*
10502442	0.0369	0.088	17.2	*Gm6214*
10386070	0.0153	0.088	5.1	*Atox1*
10353038	0.0344	0.088	16.3	*Ppp1r42*
10455135	0.0457	0.088	13.3	*Pcdhb21*
10520126	0.0423	0.088	5	*Nos3*
10594762	0.0131	0.088	11.5	*Fam81a*
10423078	0.0242	0.088	17.7	*Gm10389*
10489349	0.0057	0.088	12.6	*Gtsf1l*
10551382	0.0092	0.088	13.8	*2310022A10Rik*
10473578	0.0232	0.088	18.1	*Olfr1153*
10581650	0.0155	0.088	21.3	*Chst4*
10482788	0.0463	0.087	10.6	*A930012O16Rik*
10393917	0.0013	0.087	15.6	*Stra13*
10375461	0.0245	0.087	5.4	*Rpl7*
10569877	0.0434	0.087	23.3	*Mcemp1*
10446166	0.0311	0.087	7.4	*Ndufa11*
10434709	0.0133	0.087	13.5	*Hrg*
10491081	0.0234	0.087	17.4	---
10591177	0.0476	0.087	16	*Olfr24*
10598038	0.0435	0.087	2.5	*COX2*
10504008	0.0229	0.087	3.8	*Chmp5*
10493516	0.0418	0.087	13.7	*Gm15417*
10548057	0.0219	0.087	5.4	*Ndufa9*

10607475	0.0356	0.087	8.4	*Prdx4*
10377847	0.0395	0.087	16.2	*Gltpd2*
10563753	0.0470	0.087	16.1	*Mrgprx2*
10355628	0.0445	0.087	2.5	*Rnf25*
10529895	0.0145	0.087	5	*Qdpr*
10601565	0.0320	0.087	13.6	*Gm16420*
10419038	0.0077	0.087	5	*Ghitm*
10422026	0.0228	0.087	20.6	*Prr30*
10515399	0.0004	0.087	4.2	*Plk3*
10538567	0.0194	0.087	22.7	*Vmn1r8*
10543409	0.0273	0.087	6	*Tas2r118*
10551319	0.0260	0.087	16	*Adck4*
10511588	0.0030	0.087	14.5	*Tmem67*
10486327	0.0361	0.087	1.4	*Sptbn5*
10522248	0.0322	0.087	4.2	*Gm6517*
10479247	0.0174	0.087	2	*Fam217b*
10403834	0.0246	0.087	4.8	*Sfrp4*
10410931	0.0067	0.087	15.3	*Vcan*
10474112	0.0278	0.087	20.6	*Traf6*
10495197	0.0113	0.087	2.1	*Kcna2*
10399360	0.0147	0.087	5.7	*Rhob*
10523518	0.0335	0.087	3	*Enoph1*
10591186	0.0387	0.087	13.7	---
10439402	0.0293	0.087	4.2	*Rpl9*
10345928	0.0193	0.087	22.4	---
10542164	0.0086	0.086	18.4	*Clec12a*
10485402	0.0047	0.086	10.9	*Fjx1*
10572743	0.0444	0.086	21.1	*Olfr373*
10437668	0.0076	0.086	14.2	*Socs1*
10389331	0.0426	0.086	8.1	*Znhit3*
10338033	0.0131	0.086	14.2	---
10495340	0.0164	0.086	10.9	*Taf13*
10365495	0.0145	0.086	13.7	*BC030307*
10398966	0.0301	0.086	13.6	*Tex22*
10543551	0.0057	0.086	1.1	*Rbm28*
10557213	0.0103	0.086	16.4	*Rbbp6*
10484811	0.0425	0.086	15.3	*Olfr1232*
10393879	0.0397	0.086	1.9	*Mafg*
10529547	0.0245	0.086	16.2	*Tada2b*
10545897	0.0210	0.086	20	*Dusp11*
10368025	0.0039	0.086	1.5	*Hspa8*
10511577	0.0302	0.086	20.4	*Gm11833*
10557535	0.0449	0.086	1.2	*Sez6l2*
10498972	0.0140	0.086	1.1	*Rbm46*
10574718	0.0499	0.086	6	*Tmem208*
10587085	0.0027	0.086	12.1	*Fam214a*
10407709	0.0432	0.086	16.3	*Mtr*
10607602	0.0145	0.086	18.2	*Ppef1*

10365812	0.0203	0.086	17	*4933408J17Rik*
10591446	0.0016	0.086	10.2	*Tyk2*
10436128	0.0380	0.086	23.9	*Myh15*
10548754	0.0142	0.086	2.2	*Gprc5d*
10438328	0.0288	0.086	3.4	*Tango2*
10504458	0.0150	0.086	5.6	*Clta*
10546217	0.0071	0.086	5.4	*Chchd6*
10489151	0.0018	0.086	23.6	*Mroh8*
10551989	0.0124	0.086	16.9	*Igflr1*
10400649	0.0486	0.086	18.9	*Pole2*
10396141	0.0473	0.086	16.1	*Gm22058*
10599030	0.0129	0.086	16	*Gm26131*
10598091	0.0235	0.086	6.1	*mt-Tp*
10361956	0.0014	0.086	16.3	*Map7*
10501593	0.0356	0.086	14.9	*Slc30a7*
10395142	0.0435	0.086	14.2	*Sh3yl1*
10548139	0.0129	0.085	1.7	*Gm26338*
10338003	0.0029	0.085	1.7	---
10397621	0.0250	0.085	14.3	---
10360012	0.0005	0.085	19.9	*Fcrlb*
10450845	0.0198	0.085	8.3	*Mog*
10386455	0.0138	0.085	17.7	*Rasd1*
10605499	0.0384	0.085	15.1	*3426406K10Rik*
10411390	0.0147	0.085	13.9	*5330416C01Rik*
10350197	0.0346	0.085	3.1	*Ascl5*
10502714	0.0370	0.085	14.5	*Rpf1*
10462697	0.0260	0.085	17.4	---
10499363	0.0251	0.085	16.6	*Bglap*
10364769	0.0308	0.085	11	*Apc2*
10458583	0.0423	0.085	9.2	*Yipf5*
10435793	0.0142	0.085	17.2	*Drd3*
10345840	0.0100	0.085	21.6	*Slc9a4*
10550494	0.0035	0.085	15.1	*Gm26062*
10484693	0.0114	0.085	15.9	*Olfr1141*
10447341	0.0052	0.085	3.8	*Rhoq*
10450075	0.0047	0.085	9.4	*H2-K1*
10584231	0.0327	0.085	14.1	*Pus3*
10343384	0.0138	0.085	8.7	---
10484777	0.0435	0.085	16.2	*Olfr1216*
10393898	0.0485	0.085	3.4	*Myadml2*
10597279	0.0179	0.085	24	*Ccrl2*
10345807	0.0353	0.085	16.4	*Il18r1*
10517664	0.0345	0.085	13.4	*Gm25280*
10376019	0.0277	0.085	19.1	*Rpl9-ps3*
10378013	0.0238	0.085	14.3	*Rpain*
10493798	0.0240	0.085	5	*S100a16*
10469140	0.0168	0.085	17	*Gm13262*
10346298	0.0350	0.085	10.2	*Coq10b*

10461750	0.0007	0.085	18.7	*Olfr1443*
10358419	0.0181	0.085	15.4	*Gm23672*
10600710	0.0309	0.085	15.5	*Gm8844*
10357077	0.0468	0.085	17.8	*Serpinb3d*
10394770	0.0063	0.085	4.7	*Odc1*
10462309	0.0348	0.085	20.9	*C030016D13Rik*
10399314	0.0201	0.085	1.5	*Mfsd2b*
10543304	0.0114	0.085	14.7	*Gm23960*
10445165	0.0376	0.085	2	*Olfr127*
10354247	0.0240	0.085	17	*Fhl2*
10365968	0.0184	0.085	21.1	*Gm8601*
10455591	0.0219	0.085	6.2	---
10560174	0.0059	0.085	8.4	*Sepw1*
10556082	0.0147	0.085	2.4	*Ppfibp2*
10420628	0.0143	0.085	4.3	---
10339419	0.0101	0.085	24	---
10402988	0.0388	0.085	2.8	---
10571246	0.0028	0.085	1.5	*5930422O12Rik*
10490192	0.0339	0.084	1	*Gm14371*
10598626	0.0280	0.084	5.2	*Tspan7*
10388284	0.0076	0.084	17.3	*Olfr389*
10579939	0.0321	0.084	5.6	*Usp38*
10350749	0.0446	0.084	12.8	*Gm5531*
10448163	0.0095	0.084	15	*Vmn2r110*
10458597	0.0376	0.084	1.2	*Grxcr2*
10430725	0.0270	0.084	1.7	*St13*
10341338	0.0298	0.084	15.9	---
10527475	0.0302	0.084	13.1	*Zfp655*
10415804	0.0285	0.084	16.8	*Gm24625*
10521709	0.0127	0.084	4	*Lap3*
10405236	0.0027	0.084	10.4	*Sfxn1*
10416952	0.0211	0.084	15.7	*Mir19a*
10520388	0.0440	0.084	5.9	*Rbm33*
10515293	0.0156	0.084	1.7	*Llph*
10534537	0.0032	0.084	24	*Srcrb4d*
10465103	0.0290	0.084	22.8	*1700020D05Rik*
10530868	0.0461	0.084	14.2	*Gm25806*
10524004	0.0385	0.084	17.6	*Pcgf3*
10527530	0.0241	0.084	2.6	*Rpl21*
10470614	0.0254	0.084	15.1	*Ak8*
10534096	0.0006	0.084	7.2	*Chchd2*
10417095	0.0382	0.084	18.8	*Farp1*
10578136	0.0006	0.084	14.9	---
10503915	0.0021	0.084	7.4	*Chchd2*
10531588	0.0111	0.084	19	*Prkg2*
10427847	0.0199	0.083	17.7	*Gm17756*
10487476	0.0497	0.083	5.3	*1500011K16Rik*
10419167	0.0143	0.083	17.9	*Ptgdr*

10373396	0.0118	0.083	5.9	*Myl6*
10579657	0.0296	0.083	3.5	*Rpl23a*
10508759	0.0157	0.083	15.2	*Rpa2*
10368092	0.0105	0.083	9.6	*Hebp2*
10392735	0.0296	0.083	1.3	*Cdc42ep4*
10495993	0.0346	0.083	1	*Elovl6*
10574027	0.0385	0.083	12.5	*Mt1*
10553475	0.0460	0.083	4.4	*Rps27a*
10363967	0.0150	0.083	15.6	---
10442182	0.0177	0.083	18.2	*Vmn1r234*
10401238	0.0236	0.083	24	*Zfp36l1*
10503497	0.0392	0.083	15.3	*Slc7a13*
10381063	0.0167	0.083	15.3	*Wipf2*
10598043	0.0176	0.083	3.1	*ATP6*
10590445	0.0191	0.083	15.7	*Snrk*
10402378	0.0099	0.083	18.8	*Gm46*
10518855	0.0488	0.083	22.9	---
10605368	0.0095	0.083	17.3	*Gm25629*
10411332	0.0456	0.083	8.5	*Hmgcr*
10510624	0.0243	0.083	16.6	*Klhl21*
10344931	0.0407	0.083	4.1	*Rpl5*
10589872	0.0359	0.083	19.3	*LOC102631503*
10411776	0.0493	0.083	3.4	*Gm12271*
10453916	0.0267	0.083	16.9	*Gm25289*
10371464	0.0224	0.083	14.1	*A230060F14Rik*
10524422	0.0340	0.083	1.5	*Dao*
10606640	0.0338	0.083	14.7	*Nox1*
10451943	0.0452	0.083	22.6	*Plin5*
10490813	0.0390	0.083	18.3	---
10473809	0.0399	0.083	21.1	*Spi1*
10376239	0.0031	0.083	23.4	*Gm12238*
10568480	0.0335	0.083	9	*Nsmce4a*
10599422	0.0471	0.083	16.7	*Prr32*
10368711	0.0407	0.083	15.8	---
10482968	0.0071	0.083	4.6	*Pla2r1*
10508645	0.0383	0.083	19.3	*Snord85*
10601601	0.0467	0.083	3.4	*Rps12*
10543317	0.0358	0.083	2.4	*Gm12618*
10519691	0.0140	0.083	2.1	---
10432767	0.0146	0.083	19.1	*Gm5478*
10408121	0.0329	0.083	9.5	*Gm11285*
10591844	0.0402	0.083	14.7	*Dpy19l2*
10519310	0.0336	0.082	21.4	---
10363699	0.0411	0.082	3	*Rps6*
10504576	0.0137	0.082	20.5	*Gm829*
10350800	0.0312	0.082	11.1	*Tor1aip2*
10409804	0.0179	0.082	12.3	*Zcchc6*
10401035	0.0021	0.082	1	*Esr2*

10546031	0.0321	0.082	12.9	*Aplf*
10354372	0.0255	0.082	6.8	*Myl6*
10515003	0.0255	0.082	22.9	*Fam159a*
10572679	0.0215	0.082	12.6	*Glt25d1*
10529239	0.0364	0.082	7.3	*Pisd*
10549530	0.0259	0.082	19.3	*Gm22578*
10434845	0.0044	0.082	14.2	*Il1rap*
10417048	0.0058	0.082	15.3	*Hs6st3*
10365134	0.0189	0.082	3.1	*Gm16106*
10607454	0.0110	0.082	16.7	*Magea2*
10431948	0.0333	0.082	14.4	*Rpap3*
10420631	0.0487	0.082	9.4	*Ebpl*
10438564	0.0228	0.082	5.2	*Rps10*
10427908	0.0242	0.082	1	*Gm9948*
10599810	0.0151	0.082	15.3	*Gm25023*
10480025	0.0372	0.082	15.8	*Gm26478*
10340960	0.0415	0.082	15.5	---
10448089	0.0265	0.082	4.6	*Oaz1*
10455389	0.0393	0.082	20.3	*Scgb3a2*
10386992	0.0251	0.082	17.6	*1700086D15Rik*
10516303	0.0299	0.082	11.6	*Gm25323*
10338053	0.0156	0.082	13.6	---
10367173	0.0238	0.082	13.9	*Mip*
10349102	0.0204	0.082	15.7	*D630008O14Rik*
10373695	0.0291	0.082	11.4	*Iltifb*
10604869	0.0411	0.082	18.1	*Mir463*
10502071	0.0332	0.082	14.6	*5730508B09Rik*
10560964	0.0231	0.082	17.1	*Pou2f2*
10568024	0.0261	0.082	2.5	*Coro1a*
10386442	0.0209	0.082	10.2	*Cops3*
10559756	0.0306	0.082	4	*Vmn1r-ps45*
10566767	0.0183	0.082	16.7	*St5*
10406499	0.0496	0.082	2	*Gm12618*
10553788	0.0010	0.082	15.6	*Atp10a*
10523683	0.0048	0.082	8.2	*Dspp*
10417561	0.0106	0.082	15.2	*Fam107a*
10584393	0.0403	0.082	17.1	---
10473006	0.0253	0.082	2.8	*Gm12618*
10600980	0.0199	0.082	14.5	*Dgat2l6*
10520867	0.0408	0.082	19.4	*Gm26335*
10596070	0.0067	0.082	16.6	---
10473543	0.0140	0.082	16.8	*Pramel6*
10344837	0.0375	0.082	14.9	*Prex2*
10585834	0.0410	0.082	13.9	*6030419C18Rik*
10484606	0.0217	0.082	19.1	*Olfr1066*
10492330	0.0205	0.082	13.4	*P2ry1*
10570201	0.0144	0.082	14.7	*Atp11a*
10378024	0.0003	0.082	14	*Mis12*

10503464	0.0225	0.082	22.8	*Cngb3*
10544815	0.0172	0.081	2.8	*Hibadh*
10507520	0.0259	0.081	1.7	*Hyi*
10451110	0.0290	0.081	3.1	*Hsp90ab1*
10578771	0.0010	0.081	15.2	*Galnt7*
10596652	0.0123	0.081	4.3	*Hemk1*
10338061	0.0129	0.081	13	---
10396421	0.0251	0.081	9.6	*Hif1a*
10594965	0.0312	0.081	22.4	*Unc13c*
10571768	0.0378	0.081	15.7	*Gm23986*
10467088	0.0075	0.081	4.1	*Rpl9*
10507557	0.0306	0.081	11.8	*Ebna1bp2*
10378627	0.0208	0.081	21.7	*Rilp*
10529425	0.0236	0.081	9.3	*Nop14*
10369792	0.0044	0.081	16.3	*Arid5b*
10340546	0.0027	0.081	12.7	---
10425632	0.0484	0.081	4.4	*Xrcc6*
10476093	0.0288	0.081	8.6	*Nop56*
10462995	0.0297	0.081	20.8	---
10470959	0.0007	0.081	2.9	*Phyhd1*
10456018	0.0495	0.081	2.5	*Arsi*
10524965	0.0148	0.081	2.5	*Gm9754*
10598111	0.0172	0.081	6	*Dhrsx*
10534168	0.0049	0.081	12.5	*Auts2*
10366572	0.0255	0.081	11.6	*Iltifb*
10429500	0.0490	0.081	23.3	*Slurp1*
10362389	0.0250	0.081	23.9	---
10355916	0.0286	0.081	15.6	*Pax3*
10590242	0.0360	0.081	22	*Ccr8*
10353311	0.0128	0.081	11.1	*Jph1*
10338058	0.0046	0.081	14.6	---
10498002	0.0490	0.081	15.6	*1700018B24Rik*
10571182	0.0082	0.081	1.1	*Poteg*
10483110	0.0168	0.081	13	*Ifih1*
10339830	0.0018	0.081	13	---
10515700	0.0164	0.081	17.5	*Szt2*
10523893	0.0098	0.081	4.7	*Rpl5*
10450777	0.0201	0.081	15.5	*Gm4830*
10484486	0.0017	0.081	4.1	*P2rx3*
10358587	0.0033	0.081	16.3	*Hmcn1*
10561153	0.0231	0.081	2.1	*Cyp2b23*
10446307	0.0480	0.081	19.7	*Gm25102*
10352192	0.0189	0.081	14.6	*Gm10518*
10491753	0.0495	0.080	1	*Intu*
10530194	0.0067	0.080	4.3	*Rpl9*
10503210	0.0380	0.080	14	*Chd7*
10544538	0.0470	0.080	1.4	*Pdia4*
10539220	0.0077	0.080	11.3	*Gcfc2*

10475324	0.0225	0.080	4	*Ckmt1*
10489694	0.0147	0.080	13.9	*Zfp334*
10517521	0.0163	0.080	11.7	*Epha8*
10420457	0.0292	0.080	9.5	*Micu2*
10601616	0.0118	0.080	13.7	*Diap2*
10437151	0.0060	0.080	21.1	*Kcnj15*
10357476	0.0058	0.080	18.5	*Gm23056*
10489195	0.0001	0.080	16.4	*Tti1*
10566723	0.0131	0.080	14.5	*Lmo1*
10418171	0.0008	0.080	16.9	*Zcchc24*
10504642	0.0202	0.080	15.3	*E230008N13Rik*
10606364	0.0159	0.080	12.7	*Gm24491*
10430289	0.0050	0.080	11.6	*Ift27*
10563116	0.0132	0.080	15.7	*Flt3l*
10344071	0.0290	0.080	9.1	---
10533285	0.0331	0.080	13.7	*Ptpn11*
10592847	0.0430	0.080	6	*Myl6*
10545658	0.0039	0.080	12.9	*Wdr54*
10604633	0.0410	0.080	4.3	*Cxx1a*
10532857	0.0186	0.080	9.3	*Gltp*
10562323	0.0357	0.080	24	*Wtip*
10412919	0.0263	0.080	18.5	---
10559750	0.0265	0.080	16.1	*Vmn1r63*
10362003	0.0322	0.080	3.1	*F730021E23Rik*
10371256	0.0364	0.080	18.9	*Sirt6*
10548300	0.0121	0.080	1.9	*Klrb1*
10385776	0.0324	0.080	13.5	*Tcf7*
10498657	0.0079	0.080	15.4	---
10585494	0.0274	0.080	9	*Ube2q2*
10522925	0.0166	0.079	16.8	*Prol1*
10431124	0.0060	0.079	18.8	*Pnpla5*
10379866	0.0385	0.079	4.4	*Car4*
10585068	0.0230	0.079	7.2	*Nxpe4*
10504986	0.0389	0.079	18.9	*Gm12482*
10409118	0.0365	0.079	13.9	*Wnk2*
10406401	0.0058	0.079	14.9	---
10541741	0.0276	0.079	14	*Mlf2*
10487937	0.0215	0.079	19.6	*Prokr2*
10556463	0.0301	0.079	1.6	*Arntl*
10488926	0.0145	0.079	12.7	*BC029722*
10375341	0.0189	0.079	17.4	---
10368227	0.0404	0.079	8.7	*Ube2q2*
10543466	0.0429	0.079	6	*Gpr37*
10533071	0.0464	0.079	8.1	*Suds3*
10584259	0.0021	0.079	3.7	*Fez1*
10485070	0.0329	0.079	9	*Mdk*
10562784	0.0215	0.079	7.8	*Mybpc2*
10569014	0.0155	0.079	2.1	*Ifitm2*

10401695	0.0444	0.079	2.6	*Gm12618*
10523359	0.0464	0.079	23.2	*Cxcl13*
10584582	0.0430	0.079	23.4	---
10352777	0.0159	0.079	6.7	*Slc30a1*
10390001	0.0327	0.079	20	*Mycbpap*
10365640	0.0492	0.079	12.1	*Slc5a8*
10373363	0.0146	0.079	16.1	*Gm23241*
10390651	0.0499	0.079	16	*Gm25106*
10389775	0.0484	0.079	4.6	*Pctp*
10424825	0.0054	0.079	8.5	*Cyc1*
10546657	0.0108	0.079	17.7	*Gm25852*
10528583	0.0102	0.079	7.4	*Cdk5*
10515943	0.0101	0.079	9.7	*Ctps*
10453254	0.0130	0.079	6.9	*Cox7a2l*
10515352	0.0176	0.079	6.7	*Akr1a1*
10362729	0.0444	0.079	18.1	*Ak9*
10357332	0.0251	0.079	7.2	*Actr3*
10573916	0.0091	0.079	15.9	*Irx6*
10365297	0.0266	0.079	11.4	*D10Wsu102e*
10500982	0.0427	0.079	22.2	*I830077J02Rik*
10569222	0.0292	0.078	3	*Gm16982*
10524450	0.0047	0.078	7.8	*Ung*
10571005	0.0426	0.078	16.4	*D830025C05Rik*
10455761	0.0319	0.078	8.9	*Prdm6*
10458757	0.0080	0.078	4.6	*Morf4l1*
10592416	0.0016	0.078	2.9	*Olfr959*
10453715	0.0460	0.078	5.2	---
10600730	0.0430	0.078	16.2	*Gm5071*
10363639	0.0118	0.078	13.2	*Atoh7*
10573519	0.0187	0.078	13.5	*Tnpo2*
10443814	0.0383	0.078	20.3	*4833413E03Rik*
10445816	0.0248	0.078	24	*Lrfn2*
10590933	0.0375	0.078	2.5	*Piwil4*
10428396	0.0271	0.078	22.9	*Gm9658*
10584468	0.0359	0.078	19	*Olfr924*
10585129	0.0136	0.078	16.4	*Zw10*
10534660	0.0436	0.078	8.5	*Ap1s1*
10357222	0.0057	0.078	14.2	*Gm101*
10548829	0.0497	0.078	15.8	*Gucy2c*
10477179	0.0256	0.078	23.4	*Cox4i2*
10425335	0.0043	0.078	7.4	*Syngr1*
10538887	0.0280	0.078	1	*Igkv2-112*
10557035	0.0024	0.078	14.2	*Polr3e*
10534202	0.0061	0.078	18.5	*Ncf1*
10447510	0.0482	0.078	3.6	*Amd1*
10603245	0.0364	0.078	24	*Gm2182*
10428912	0.0244	0.078	4.6	*Fam84b*
10515920	0.0316	0.078	10.1	*Ppcs*

10455259	0.0315	0.078	11.9	*Arhgap26*
10543411	0.0395	0.078	16	*Slc13a1*
10514789	0.0477	0.078	3.5	*Gm12722*
10384685	0.0279	0.078	12.3	*1700093K21Rik*
10379968	0.0150	0.078	17.1	*Tubd1*
10440593	0.0491	0.078	15	*Rwdd2b*
10488655	0.0261	0.078	15.1	*Bcl2l1*
10382653	0.0216	0.078	21.2	*Myo15b*
10506148	0.0435	0.078	20.9	*Gm12689*
10559764	0.0167	0.078	2.2	*Zfp787*
10525471	0.0231	0.078	4.7	*Ndufb11*
10438308	0.0499	0.078	13.7	*Ranbp1*
10542407	0.0169	0.078	1.4	*Pde6h*
10363231	0.0325	0.078	3.1	*Smpdl3a*
10413932	0.0151	0.078	1.7	*Vstm4*
10550870	0.0326	0.078	4.6	*Lypd5*
10526427	0.0154	0.077	23.3	*Dtx2*
10451993	0.0266	0.077	22.6	*D17Wsu104e*
10567343	0.0089	0.077	12.5	*Knop1*
10551750	0.0012	0.077	2.6	*4932431P20Rik*
10400470	0.0409	0.077	4.9	*Cox6c*
10557450	0.0486	0.077	9.3	*Bola2*
10392437	0.0172	0.077	7.3	*Gm11696*
10520288	0.0362	0.077	10.5	*Galnt11*
10541494	0.0205	0.077	4.5	*Rps27a*
10529034	0.0049	0.077	12.1	*Cgref1*
10472686	0.0037	0.077	4.3	*Rpl9*
10564614	0.0344	0.077	12.1	*Gm26176*
10370242	0.0383	0.077	3.3	*Pcbp3*
10570141	0.0181	0.077	4	*1700128E19Rik*
10598055	0.0385	0.077	2.4	*ND3*
10436182	0.0088	0.077	3.1	*Cd47*
10464287	0.0489	0.077	14.2	*Ccdc172*
10458589	0.0443	0.077	19.3	*Prelid2*
10570180	0.0321	0.077	2.4	*Gm5607*
10455098	0.0495	0.077	17.4	*Pcdhb14*
10570002	0.0097	0.077	14.4	*2410089E03Rik*
10583952	0.0061	0.077	14.1	*Ncapd3*
10409345	0.0142	0.077	5.6	*Cltb*
10397052	0.0401	0.077	23.4	*Gm10006*
10441858	0.0201	0.077	17	*Smok2a*
10560709	0.0329	0.077	18.5	*Pvr*
10606439	0.0311	0.077	1	*Gm25797*
10399379	0.0193	0.077	4.4	*Pgk1*
10498313	0.0193	0.077	4.4	*Pgk1*
10575476	0.0004	0.077	13.4	*Vac14*
10431137	0.0000	0.077	16.2	---
10503134	0.0232	0.077	4.3	*Sdcbp*

10497817	0.0207	0.077	4.9	*Anxa5*
10445607	0.0387	0.077	1	*Pex6*
10415844	0.0301	0.077	3.6	*Ctsb*
10573128	0.0301	0.077	12.5	*Tbc1d9*
10476614	0.0386	0.077	16.5	*Rpl26-ps5*
10600536	0.0094	0.077	18	*Gm4937*
10487510	0.0080	0.077	14.5	*Gm14006*
10348062	0.0398	0.077	5.2	*B3gnt7*
10601044	0.0071	0.077	1	*Gdpd2*
10439442	0.0436	0.077	4	*Pla1a*
10402444	0.0180	0.076	7	*Dicer1*
10412665	0.0477	0.076	2.9	*Gm12618*
10601086	0.0001	0.076	15.3	*Gm23541*
10474984	0.0252	0.076	16.2	*Nusap1*
10427657	0.0082	0.076	18.2	*Spef2*
10576386	0.0004	0.076	10.8	*Rhou*
10414787	0.0033	0.076	15.9	*Trav5-4*
10414897	0.0033	0.076	15.9	*Trav5-4*
10479950	0.0368	0.076	12.4	*Celf2*
10562709	0.0244	0.076	4.9	*Cd33*
10518989	0.0299	0.076	15.5	*Trp73*
10371240	0.0183	0.076	17.8	*Tle6*
10410690	0.0125	0.076	20.6	---
10489186	0.0039	0.076	3	*Rpl35a*
10455108	0.0299	0.076	15.6	*Pcdhb16*
10370541	0.0456	0.076	15.6	---
10543802	0.0145	0.076	11.9	*Plxna4*
10421922	0.0273	0.076	24	---
10448416	0.0113	0.076	1.6	*Kctd5*
10356457	0.0058	0.076	8.8	*Dnajb3*
10583044	0.0091	0.076	16.9	*Mmp13*
10418341	0.0071	0.076	15.3	*Il17rb*
10444788	0.0031	0.076	2.5	*H2-Q1*
10338008	0.0222	0.076	14.9	---
10439891	0.0466	0.076	2.7	*Gm6767*
10600892	0.0196	0.076	17.6	*Pgr15l*
10558936	0.0016	0.076	17.6	*Efcab4a*
10458958	0.0092	0.076	2.9	*Gm26959*
10508444	0.0022	0.076	7.6	*Zbtb8os*
10406825	0.0338	0.076	5.2	*Ankra2*
10368473	0.0396	0.076	16.8	*Gm22370*
10513190	0.0308	0.076	16.1	*D630039A03Rik*
10379026	0.0035	0.076	15.3	*Mir144*
10528648	0.0459	0.076	9.3	*Abcf2*
10368056	0.0405	0.076	11.9	*Ect2l*
10473498	0.0068	0.076	16.9	*Olfr1038-ps*
10429329	0.0138	0.076	16	*Ago2*
10598547	0.0440	0.076	21.1	*Gm6798*

10484205	0.0239	0.075	12.7	*Ccdc141*
10438098	0.0223	0.075	23.4	*Sdf2l1*
10533569	0.0054	0.075	16.4	*Kdm2b*
10381218	0.0437	0.075	14.7	*Hsd17b1*
10418004	0.0274	0.075	8.8	*Ap3m1*
10358283	0.0320	0.075	3.7	*Crb1*
10580061	0.0204	0.075	4.7	*Il27ra*
10489569	0.0018	0.075	8.6	*Pltp*
10478799	0.0230	0.075	10	*Cse1l*
10361007	0.0253	0.075	8.4	*Smyd2*
10362416	0.0016	0.075	16.2	*Trdn*
10443704	0.0473	0.075	18.7	*Umodl1*
10603702	0.0177	0.075	1.7	*Llph*
10515086	0.0043	0.075	15.6	*9630013D21Rik*
10591781	0.0287	0.075	21.2	*Anln*
10484783	0.0065	0.075	16.9	*Olfr1219*
10460926	0.0304	0.075	14.1	*Sf1*
10531707	0.0120	0.075	14.4	*Lin54*
10460513	0.0080	0.075	4.7	*B3gnt1*
10516544	0.0498	0.075	11.2	*Hpca*
10371502	0.0288	0.075	5.7	*Fabp3*
10492294	0.0434	0.075	17.1	*Aadacl2*
10392040	0.0061	0.075	16.1	*Mar 10*
10568865	0.0012	0.075	19.1	*6430531B16Rik*
10404731	0.0454	0.075	5.6	*Tmem14c*
10443369	0.0315	0.075	18.8	*Armc12*
10431558	0.0380	0.075	15.8	*Odf3b*
10460257	0.0460	0.075	22	*Aldh3b2*
10339741	0.0013	0.075	5.5	---
10490159	0.0004	0.075	12.4	*Pmepa1*
10441565	0.0343	0.075	1	*Rps6ka2*
10578521	0.0272	0.075	15.5	*Snx25*
10425207	0.0430	0.075	7.2	*H1f0*
10379633	0.0069	0.075	16.9	*Slfn1*
10582694	0.0297	0.075	5.8	*Fam89a*
10500999	0.0419	0.075	15.2	*Pifo*
10479379	0.0115	0.075	1.6	*Slco4a1*
10551953	0.0035	0.075	2.3	*Arhgap33os*
10558914	0.0292	0.075	9	*Rplp2*
10382243	0.0008	0.075	14.6	*Gna13*
10559373	0.0420	0.075	15.2	*Fgf15*
10567823	0.0468	0.075	2.8	*Gm12618*
10561712	0.0444	0.075	6.9	*Spint2*
10556200	0.0115	0.074	4.5	*Rpl27a*
10566609	0.0046	0.074	20.2	*Olfr706*
10577528	0.0481	0.074	10	*Smim19*
10409629	0.0375	0.074	12.9	*Klhl3*
10492662	0.0454	0.074	9.1	*Gm17359*

10351298	0.0253	0.074	15.3	*Gpr161*
10392936	0.0375	0.074	10.3	*Nt5c*
10502973	0.0207	0.074	16.1	*Tyw3*
10379176	0.0149	0.074	16.1	*Unc119*
10511325	0.0332	0.074	5.8	*Gm4997*
10403220	0.0496	0.074	3.1	*Abcb5*
10598664	0.0402	0.074	6.3	*Atp6ap2*
10391697	0.0307	0.074	6.1	*Itga2b*
10365887	0.0092	0.074	3.2	*Ndufa12*
10354542	0.0380	0.074	9.5	*Pms1*
10608675	0.0386	0.074	4.2	---
10499309	0.0279	0.074	5.8	*Apoa1bp*
10503584	0.0141	0.074	4.2	*Coq3*
10469951	0.0141	0.074	8.8	*Rnf208*
10493555	0.0105	0.074	1	*Kcnn3*
10430358	0.0325	0.074	3.9	*C1qtnf6*
10447139	0.0481	0.074	2.8	*Gm12618*
10542872	0.0153	0.074	13.4	*Rps4l*
10604674	0.0012	0.074	15	*Gm773*
10409204	0.0076	0.074	6.3	*Gm10784*
10408850	0.0199	0.074	2.4	*Nedd9*
10495316	0.0069	0.074	24	*Psrc1*
10488567	0.0186	0.074	13.7	*Rad21l*
10552286	0.0027	0.074	15.9	*Gm25784*
10552306	0.0148	0.074	20.5	*Gm9246*
10473363	0.0237	0.074	24	*Timm10*
10528691	0.0258	0.074	5.6	*Rheb*
10518428	0.0388	0.074	12.4	*Clcn6*
10367579	0.0145	0.074	20.1	*Gm3213*
10560217	0.0263	0.074	15	*Dhx34*
10436041	0.0044	0.074	9.6	---
10347662	0.0074	0.074	13	*Tmem198*
10461594	0.0441	0.073	15.9	*Ms4a4c*
10338028	0.0368	0.073	14.6	---
10568361	0.0237	0.073	7.2	*Yipf5*
10368577	0.0464	0.073	18.1	*Rnf217*
10517988	0.0362	0.073	17.2	*Slc25a34*
10436734	0.0209	0.073	22.1	*Bach1*
10448998	0.0499	0.073	2.7	*Gm25083*
10492824	0.0261	0.073	15.9	*Tmem154*
10427895	0.0380	0.073	12.4	*Basp1*
10425757	0.0079	0.073	7.1	*Smdt1*
10498273	0.0463	0.073	6.4	*Tm4sf1*
10402659	0.0320	0.073	4.3	*Ankrd9*
10488550	0.0098	0.073	8.7	*Nanp*
10436945	0.0429	0.073	20.6	*Slc5a3*
10467216	0.0493	0.073	7.7	*Cpeb3*
10381619	0.0271	0.073	17	*Adam11*

10365288	0.0030	0.073	7.1	*Taf10*
10446207	0.0043	0.073	12.7	*Clpp*
10595048	0.0399	0.073	16	*Gm25300*
10372091	0.0498	0.073	15.6	*Anapc15*
10342281	0.0440	0.073	17.5	---
10508614	0.0391	0.073	5.1	*Fabp3*
10568260	0.0487	0.073	1	*Zfp629*
10587651	0.0471	0.073	2.7	*Gm12618*
10404612	0.0151	0.073	17	*Rreb1*
10576152	0.0363	0.073	6.6	*Trappc2l*
10338433	0.0004	0.073	13.5	---
10515481	0.0293	0.073	14.3	*Dmap1*
10551077	0.0439	0.073	12	*9130221H12Rik*
10552752	0.0151	0.073	18.9	*Akt1s1*
10468231	0.0008	0.073	4.5	*Arl3*
10588195	0.0347	0.073	22.2	*9630041A04Rik*
10338048	0.0047	0.073	15.5	---
10425263	0.0317	0.073	4.5	*Gm10864*
10473750	0.0271	0.073	10.6	*C1qtnf4*
10512830	0.0110	0.072	14.5	*Anks6*
10428336	0.0189	0.072	9.1	*Mir692-3*
10426924	0.0252	0.072	24	*Slc4a8*
10424404	0.0334	0.072	9.3	*Pvt1*
10556208	0.0455	0.072	17.5	*Akip1*
10565689	0.0333	0.072	19.9	*Capn5*
10364909	0.0362	0.072	4.7	*Oaz1*
10600602	0.0007	0.072	16.1	*Gm22686*
10566315	0.0286	0.072	18.3	*E030002O03Rik*
10428204	0.0140	0.072	14.9	*Ywhaz*
10367179	0.0039	0.072	23.3	*Timeless*
10426298	0.0203	0.072	22.3	---
10426601	0.0382	0.072	8.7	*Olfr279*
10513166	0.0048	0.072	15.5	*Ptpn3*
10505213	0.0298	0.072	15.1	*E130308A19Rik*
10338012	0.0004	0.072	15.4	---
10497343	0.0483	0.072	5.9	*Gm16399*
10440977	0.0391	0.072	6.7	*Atp5o*
10508829	0.0277	0.072	12.7	*Map3k6*
10543017	0.0201	0.072	16.8	*Pdk4*
10524955	0.0205	0.072	6.2	*Tesc*
10567316	0.0065	0.072	12.8	*Tmc7*
10454805	0.0082	0.072	3.7	*Uba52*
10425430	0.0080	0.072	18.5	*Tnrc6b*
10397476	0.0039	0.072	12.6	*Cipc*
10431486	0.0415	0.072	11.4	*Sbf1*
10544452	0.0425	0.072	21.2	*Fam115c*
10484207	0.0448	0.072	14.9	*Ccdc141*
10485466	0.0299	0.072	10.4	*Cat*

10483249	0.0499	0.072	23.7	*Galnt3*
10502686	0.0482	0.072	19.3	*Dnase2b*
10598422	0.0229	0.072	13.3	*Gripap1*
10373016	0.0467	0.072	2.5	*LOC100504608*
10503021	0.0185	0.072	17.1	*Gm23725*
10477485	0.0456	0.072	1.4	*Bpifa5*
10514388	0.0296	0.072	15.7	---
10338021	0.0004	0.072	15.4	---
10338011	0.0071	0.072	13.8	---
10376425	0.0222	0.072	14.6	*LOC102642717*
10601390	0.0333	0.072	4.7	*Pgk1*
10408138	0.0079	0.072	16.7	*Vmn1r202*
10539933	0.0276	0.072	7.5	*Txnrd3*
10574934	0.0371	0.072	14.1	*Nrn1l*
10431220	0.0251	0.072	13.1	*Cdpf1*
10571889	0.0058	0.071	23.1	*Gm26164*
10602586	0.0467	0.071	12.7	*Mirlet7f-2*
10482866	0.0316	0.071	21.7	---
10409599	0.0422	0.071	2.8	---
10528289	0.0406	0.071	17.4	*Ccdc146*
10425319	0.0355	0.071	1	*Gm10856*
10521036	0.0033	0.071	3.4	*Rpl35a*
10439130	0.0045	0.071	1.7	*Umps*
10345368	0.0174	0.071	1	*D1Ertd448e*
10486185	0.0293	0.071	24	*Dnajc17*
10522868	0.0172	0.071	15.7	*Prr27*
10353630	0.0208	0.071	2.9	*COX2*
10512265	0.0259	0.071	14	*Fam219a*
10397416	0.0379	0.071	15.6	*Ift43*
10351832	0.0387	0.071	16.1	*Ccdc19*
10531261	0.0034	0.071	17.3	*Rassf6*
10526482	0.0205	0.071	13.1	*Polr2j*
10461568	0.0280	0.071	14.1	*Prpf19*
10387909	0.0311	0.071	15.9	*Chrne*
10415678	0.0079	0.071	15	*Cab39l*
10513805	0.0077	0.071	12.6	*Brinp1*
10534551	0.0113	0.071	5.4	*Lrwd1*
10437765	0.0199	0.071	11.5	*Cpped1*
10524588	0.0466	0.071	16.6	*Ankrd13a*
10393544	0.0156	0.071	15.5	*Cyth1*
10513955	0.0162	0.071	15.2	*Gm22864*
10587226	0.0345	0.071	14.5	*Lysmd2*
10396740	0.0272	0.071	14.6	*Gphn*
10588855	0.0333	0.071	23.5	*Mst1*
10440037	0.0084	0.071	7.5	*Nit2*
10520124	0.0453	0.071	3.7	*Sumo2*
10460376	0.0186	0.071	6.3	*Ppp1ca*
10475218	0.0208	0.070	16.8	*Stard9*

10469793	0.0457	0.070	17.3	*Il1f6*
10360737	0.0047	0.070	3.2	*Rpl35a*
10427849	0.0420	0.070	10.8	*6030458C11Rik*
10485307	0.0116	0.070	15	*Mir129-2*
10377927	0.0376	0.070	5	*Rnf167*
10566926	0.0341	0.070	5.6	*Rnf141*
10607486	0.0430	0.070	1	*Ptchd1*
10398193	0.0137	0.070	3.3	*3110018I06Rik*
10530151	0.0423	0.070	16.2	*Tlr6*
10493789	0.0128	0.070	4.8	*S100a13*
10417027	0.0247	0.070	4.3	*Cldn10*
10504562	0.0171	0.070	13.7	*Exosc3*
10488673	0.0437	0.070	24	*Foxs1*
10512739	0.0477	0.070	10.5	*Xpa*
10409265	0.0354	0.070	5.7	*Auh*
10597575	0.0466	0.070	13.2	*Plcd1*
10349208	0.0440	0.070	1	*Cntnap5a*
10484261	0.0149	0.070	14	*Cerkl*
10412333	0.0368	0.070	16.6	*Gm10733*
10410506	0.0063	0.070	4.3	*Rpl9*
10427162	0.0242	0.070	13.5	*Mfsd5*
10382830	0.0137	0.070	24	*Gm11744*
10383133	0.0217	0.070	2.4	*Slc26a11*
10386352	0.0325	0.070	2.4	*Gjc2*
10436239	0.0212	0.070	19.4	*Zbtb11*
10525489	0.0104	0.070	15.8	*Setd1b*
10465424	0.0389	0.070	14.5	*Gm14964*
10451884	0.0334	0.070	3.1	*Gm12618*
10468639	0.0394	0.070	17.6	*Dclre1a*
10589503	0.0484	0.070	5.1	*Elp6*
10338010	0.0008	0.070	15.5	---
10556246	0.0354	0.070	16.6	*Zfp143*
10511180	0.0391	0.070	13.5	*Mxra8*
10352292	0.0132	0.069	14.9	*G370120E05Rik*
10413398	0.0243	0.069	15.4	*Il17rd*
10478219	0.0206	0.069	12.8	*Plcg1*
10349661	0.0049	0.069	1	*5430435G22Rik*
10568436	0.0439	0.069	3.6	*Fgfr2*
10469637	0.0194	0.069	2.6	*Myo3a*
10578222	0.0438	0.069	17.4	*Dlc1*
10500802	0.0386	0.069	16.6	*Atg4a*
10503382	0.0135	0.069	16.6	*Runx1t1*
10372748	0.0170	0.069	15.1	*Gm10744*
10490164	0.0006	0.069	18.4	*Ankrd60*
10348119	0.0376	0.069	15.1	---
10476653	0.0411	0.069	4.6	*Banf2*
10487969	0.0013	0.069	13.7	*Trmt6*
10344741	0.0250	0.069	2.9	*Hnrnpa3*

10515113	0.0250	0.069	2.9	*Hnrnpa3*
10517808	0.0444	0.069	2.9	*Padi3*
10574676	0.0001	0.069	10.4	*Nol3*
10360076	0.0245	0.069	6.1	*Ndufs2*
10383472	0.0477	0.069	14.4	*Rac3*
10604602	0.0021	0.069	14.9	*Mir450b*
10569830	0.0173	0.069	3.3	*Camsap3*
10526356	0.0090	0.069	9.6	*Rhbdd2*
10458081	0.0304	0.069	16.2	---
10598562	0.0124	0.069	5.9	*B630019K06Rik*
10555414	0.0190	0.069	4.1	*Rab6a*
10563085	0.0160	0.069	10.5	*Fcgrt*
10606735	0.0133	0.069	11.8	*Armcx2*
10398972	0.0389	0.069	11.7	*Mta1*
10515797	0.0299	0.069	6.1	*Tmem125*
10488382	0.0197	0.069	1.6	*Cd93*
10464914	0.0009	0.069	3.6	*Tmem151a*
10414612	0.0497	0.069	21.4	*Slc39a2*
10400544	0.0480	0.069	2.9	*Gm12618*
10443215	0.0352	0.069	12	*Snrpc*
10383756	0.0197	0.069	1.7	*Ifitm2*
10345206	0.0049	0.069	1.6	*Gm7910*
10451856	0.0300	0.069	8.2	*Vmn2r118*
10369038	0.0473	0.069	17.8	*4933411G06Rik*
10560719	0.0235	0.069	1.7	*Igsf23*
10490856	0.0172	0.069	8.4	*Ralyl*
10528880	0.0461	0.068	9.4	*Lmbr1*
10387816	0.0105	0.068	5.6	*Rnasek*
10515201	0.0246	0.068	22.4	*Cyp4b1*
10464218	0.0140	0.068	16.5	*Fam160b1*
10581214	0.0392	0.068	17.1	*Lrrc29*
10598638	0.0344	0.068	7	*Mid1ip1*
10537231	0.0193	0.068	18.3	*Stra8*
10425601	0.0148	0.068	13.5	*Tef*
10338006	0.0011	0.068	15.3	---
10461782	0.0423	0.068	2.8	*Rmnd1*
10496262	0.0400	0.068	24	*Slc9b2*
10543120	0.0398	0.068	1.2	*Ica1*
10597573	0.0189	0.068	6.8	*Eif1*
10368566	0.0117	0.068	13.6	*Tpd52l1*
10588874	0.0211	0.068	6.8	*F630040L22Rik*
10357220	0.0177	0.068	1.4	*Tmem177*
10384233	0.0290	0.068	15.4	*Tns3*
10394735	0.0260	0.068	21.7	*Pdia6*
10500445	0.0100	0.068	15.4	*Chd1l*
10457798	0.0271	0.068	18.1	*Ccdc178*
10494769	0.0428	0.068	12.5	*Trim45*
10531274	0.0249	0.068	12.4	*Btc*

10559987	0.0041	0.068	15.3	*Mir297-1*
10338044	0.0156	0.068	24	---
10363563	0.0485	0.068	8.9	*Slc25a16*
10441680	0.0224	0.068	16.8	*Pde10a*
10505436	0.0173	0.068	3	*Mir455*
10484685	0.0184	0.068	14.1	*Olfr1135*
10563014	0.0327	0.068	16.8	*Prmt1*
10452356	0.0074	0.068	15.7	*Gpr108*
10407535	0.0106	0.068	5.1	*Rpl10a*
10371796	0.0124	0.068	3.4	*Slc17a8*
10483439	0.0433	0.068	19	*Lrp2*
10516753	0.0456	0.068	2.7	*Gm853*
10381776	0.0030	0.068	3.5	*Mapt*
10552932	0.0236	0.068	12.5	*Kcna7*
10428353	0.0422	0.068	12	*Lrp12*
10541484	0.0455	0.068	8.7	*M6pr*
10466865	0.0473	0.068	15.2	*Rfx3*
10442638	0.0397	0.068	6.6	*Mrps34*
10489179	0.0306	0.068	21.3	*Ghrh*
10357946	0.0260	0.068	17.7	*Ppp1r12b*
10349913	0.0128	0.068	14.5	*Kiss1*
10529661	0.0085	0.068	22.9	*Zbtb49*
10429452	0.0367	0.068	16.2	*Sf3b4*
10440600	0.0334	0.068	6.1	*Cct8*
10491977	0.0097	0.067	14.8	*Proser1*
10401488	0.0092	0.067	16.7	*Abcd4*
10405890	0.0051	0.067	4	*Rpl9*
10526055	0.0066	0.067	7.6	*Gbas*
10518642	0.0012	0.067	11.7	*Ube4b*
10500990	0.0076	0.067	9.1	*Atp5f1*
10338067	0.0022	0.067	15.1	---
10552284	0.0418	0.067	4.8	*Pin4*
10398759	0.0233	0.067	21.2	*Tdrd9*
10499945	0.0192	0.067	14.7	*Lce1l*
10464905	0.0328	0.067	17	*Npas4*
10369174	0.0238	0.067	16.7	*Gm25602*
10505282	0.0049	0.067	14.3	*Prpf4*
10426603	0.0160	0.067	16.8	*9330020H09Rik*
10437664	0.0134	0.067	8.8	*Dexi*
10595718	0.0292	0.067	13.4	*Chst2*
10432439	0.0317	0.067	4.6	*Fmnl3*
10542470	0.0463	0.067	8.3	*Mgst1*
10518781	0.0017	0.067	15.2	*Per3*
10533734	0.0451	0.067	6.3	*Abcb9*
10424349	0.0155	0.067	6.1	*Sqle*
10491062	0.0299	0.067	4.3	*Rpl27a*
10338023	0.0169	0.067	15.5	---
10541968	0.0150	0.067	14.8	*Ano2*

10456904	0.0171	0.067	17.5	*Pstpip2*
10554321	0.0306	0.067	22.4	*Gm10616*
10564631	0.0054	0.066	2.5	*Slco3a1*
10517488	0.0223	0.066	15.2	*Ephb2*
10417912	0.0319	0.066	15.9	*Usp54*
10574412	0.0178	0.066	5	*Sap18*
10338052	0.0016	0.066	15.7	---
10435271	0.0328	0.066	11.4	*Heg1*
10445237	0.0297	0.066	7	*Ptchd4*
10599309	0.0340	0.066	15.7	*Rhox10*
10507677	0.0007	0.066	17.4	*Hivep3*
10376726	0.0190	0.066	24	*Dhrs7b*
10375667	0.0257	0.066	7.6	*Rnf130*
10535653	0.0221	0.066	11.2	*Zkscan14*
10368144	0.0114	0.066	1.6	*Tnfaip3*
10470390	0.0187	0.066	22.2	*Gm13397*
10473224	0.0073	0.066	1	*Dusp19*
10367106	0.0116	0.066	4.4	*Atp5b*
10608083	0.0093	0.066	15.3	---
10514568	0.0027	0.066	6.4	*Tm2d1*
10473539	0.0349	0.066	13.7	*Olfr1124*
10500780	0.0183	0.066	13.8	*Nr1h5*
10598059	0.0421	0.066	3	*ND4*
10397482	0.0037	0.066	13.6	*Tmem63c*
10542596	0.0415	0.066	8.1	*Slco1c1*
10546001	0.0309	0.066	14.1	*Gkn3*
10553299	0.0260	0.066	2	*Ifitm2*
10398392	0.0408	0.066	13.7	*Mir329*
10497590	0.0024	0.066	16	*Mecom*
10360684	0.0062	0.066	10.6	*Ephx1*
10582390	0.0493	0.066	4.3	*Aprt*
10600576	0.0472	0.066	14.5	*Gm14743*
10433719	0.0003	0.066	13.6	*Mir484*
10470182	0.0384	0.066	8	*Bmyc*
10499080	0.0352	0.066	15.7	*Arfip1*
10455338	0.0491	0.066	2.4	*Gm12618*
10495931	0.0008	0.066	13.9	*Mir367*
10405287	0.0379	0.065	5.1	*Higd2a*
10441195	0.0182	0.065	15.1	*Dscam*
10422249	0.0402	0.065	18.6	*Dct*
10444665	0.0027	0.065	12.8	*Ddah2*
10376017	0.0256	0.065	18.3	*Gm9945*
10390831	0.0231	0.065	15.3	*Krt10*
10449549	0.0034	0.065	3.6	*Rpl35a*
10485685	0.0034	0.065	3.6	*Rpl35a*
10533626	0.0034	0.065	3.6	*Rpl35a*
10538080	0.0034	0.065	3.6	*Rpl35a*
10574087	0.0035	0.065	13	*Herpud1*

10528610	0.0282	0.065	3.6	*Tmub1*
10407097	0.0430	0.065	11.2	*Pde4d*
10387014	0.0464	0.065	4.4	*Map2k4*
10523056	0.0008	0.065	15.7	---
10541075	0.0133	0.065	17.6	*Cxcl12*
10433721	0.0107	0.065	14.3	*Nde1*
10461108	0.0439	0.065	7.9	*Iscu*
10355806	0.0330	0.065	2.8	*Tuba4a*
10457487	0.0492	0.065	15	*Mir1a-2*
10364373	0.0225	0.065	5.4	*Lsm7*
10518976	0.0226	0.065	1	*Ccdc27*
10512714	0.0293	0.065	18.4	*Shb*
10410016	0.0183	0.065	17.3	*Fancc*
10356778	0.0500	0.065	4	*Rpl17*
10347226	0.0390	0.065	23.1	*Tmem169*
10502606	0.0407	0.065	14.9	*Gm6289*
10465474	0.0167	0.065	4.5	*Prdx5*
10451679	0.0420	0.065	19.5	*Daam2*
10596680	0.0472	0.065	22.5	*Sema3b*
10480381	0.0494	0.065	13.4	*Arhgap21*
10432431	0.0396	0.065	20.4	*Fam186b*
10578613	0.0348	0.065	6.5	*Rps16*
10555746	0.0221	0.065	14.5	*Olfr568*
10407507	0.0180	0.065	3.5	*Rpl35a*
10593865	0.0000	0.065	10.6	*Rn45s*
10498921	0.0470	0.064	12.6	*Tdo2*
10470741	0.0300	0.064	3.8	*Coq4*
10520173	0.0339	0.064	8	*Asic3*
10441361	0.0160	0.064	19.8	*Tiam2*
10361846	0.0323	0.064	13.5	*Reps1*
10430105	0.0206	0.064	1.9	*C030006K11Rik*
10406205	0.0064	0.064	10.3	*Erap1*
10518344	0.0378	0.064	2.9	*Cdv3*
10389786	0.0053	0.064	12.2	*Hlf*
10338007	0.0068	0.064	15.1	---
10527213	0.0250	0.064	18.4	*Daglb*
10338031	0.0006	0.064	15.3	---
10403765	0.0393	0.064	4.9	*Vps41*
10552516	0.0097	0.064	2.7	*Klk6*
10460057	0.0068	0.064	12.4	*Tshz1*
10563220	0.0248	0.064	7	*Ppfia3*
10434643	0.0199	0.064	6.3	*Psmb3*
10374529	0.0415	0.064	16.2	*Wdpcp*
10606495	0.0175	0.064	15.6	*Pof1b*
10570590	0.0106	0.064	23.7	*Spag11b*
10424804	0.0448	0.064	7.2	*Exosc4*
10534743	0.0032	0.064	24	*Zan*
10382802	0.0464	0.064	2.7	*Sphk1*

10417605	0.0336	0.064	18.3	*Fhit*
10604604	0.0001	0.064	16	*Mir450-1*
10489049	0.0029	0.064	4.6	*Rpl9*
10446715	0.0099	0.064	3.8	*Fam179a*
10461164	0.0316	0.064	7.6	*Wdr74*
10338022	0.0013	0.064	15.5	---
10455454	0.0351	0.063	15.8	*Gm10542*
10338032	0.0008	0.063	15.5	---
10550605	0.0380	0.063	15.2	*Eml2*
10439063	0.0385	0.063	14.4	*Fbxo45*
10458245	0.0282	0.063	16.5	*Gm26109*
10435187	0.0075	0.063	3.3	*Rpl35a*
10408684	0.0484	0.063	4.8	*Tmed10*
10338034	0.0207	0.063	15.7	---
10549546	0.0191	0.063	6.1	*Ndufa3*
10410124	0.0260	0.063	7.5	*Ctsl*
10468783	0.0262	0.063	16.6	*Vax1*
10559500	0.0179	0.063	16.8	*D030047H15Rik*
10463661	0.0148	0.063	24	*Trim8*
10534152	0.0272	0.063	11.6	*Wbscr17*
10338005	0.0008	0.063	15.2	---
10338014	0.0007	0.063	15.4	---
10376144	0.0376	0.063	12	*Fnip1*
10558248	0.0111	0.063	8.3	*Bub3*
10392241	0.0355	0.063	15.3	*Polg2*
10525495	0.0103	0.063	15.2	*Setd1b*
10411395	0.0396	0.063	13.7	*Arhgef28*
10338057	0.0021	0.063	15.6	---
10489253	0.0313	0.063	16.2	*Zhx3*
10369783	0.0231	0.063	8.2	*Zfp365*
10511779	0.0464	0.063	19.5	*Atp6v0d2*
10452643	0.0309	0.063	3.5	*Myl12a*
10459452	0.0221	0.063	12.8	*A330084C13Rik*
10586971	0.0394	0.063	17.2	*Prtg*
10478075	0.0418	0.063	2.8	*Gm25129*
10436941	0.0046	0.063	16.9	*Mrps6*
10429448	0.0243	0.063	19.1	---
10497149	0.0120	0.063	3.7	*Wls*
10473160	0.0022	0.063	16.7	*Ssfa2*
10598279	0.0338	0.063	13.3	*Akap4*
10594904	0.0098	0.062	4.4	*Uba52*
10562152	0.0165	0.062	7	*Mag*
10580155	0.0120	0.062	6.2	*D8Ertd738e*
10376813	0.0311	0.062	2.2	*Specc1*
10460746	0.0464	0.062	17.4	*Naaladl1*
10424573	0.0298	0.062	5	---
10388042	0.0340	0.062	7.8	*6330403K07Rik*
10570556	0.0076	0.062	4.3	*Mcph1*

10442283	0.0037	0.062	14.1	*Gm9805*
10396936	0.0104	0.062	13.8	*Smoc1*
10496475	0.0338	0.062	10.4	*Adh5*
10458398	0.0487	0.062	11	*Hars*
10384603	0.0179	0.062	5.2	*Mdh1*
10354598	0.0289	0.062	15.4	*Hecw2*
10531100	0.0106	0.062	15.5	*Sult1d1*
10481453	0.0315	0.062	8.6	*Dolk*
10428744	0.0240	0.062	3.8	*9130401M01Rik*
10444066	0.0190	0.062	7.6	*Zbtb22*
10420225	0.0087	0.062	19	*Cma1*
10415319	0.0272	0.062	17.6	*Irf9*
10543781	0.0482	0.062	14.3	*Mir29b-1*
10435455	0.0029	0.062	2.8	*Rpl35a*
10387568	0.0132	0.062	15	*Tnfsf13*
10491191	0.0303	0.062	5.8	*Rpl22l1*
10419450	0.0317	0.062	22.6	*Olfr733*
10514195	0.0336	0.062	16.8	*Fam154a*
10338046	0.0209	0.062	15.7	---
10541862	0.0109	0.062	12.3	*Iffo1*
10544540	0.0141	0.062	3.6	*Rpl35a*
10532407	0.0445	0.062	12.8	*Ep400*
10450055	0.0198	0.062	3.7	*Pfdn6*
10494662	0.0131	0.062	5	*Ywhah*
10406111	0.0460	0.062	3.4	*Slc12a7*
10512061	0.0369	0.062	6.7	*Taf9*
10541191	0.0407	0.062	16.7	*Rad52*
10481566	0.0487	0.061	4.7	*Fibcd1*
10556901	0.0374	0.061	13	*Abca16*
10525726	0.0231	0.061	12.4	*2810006K23Rik*
10479869	0.0351	0.061	6.1	*Cdc123*
10345580	0.0177	0.061	13.3	*Inpp4a*
10561806	0.0253	0.061	8.4	*Mif*
10447742	0.0255	0.061	21.1	*Map3k4*
10559946	0.0488	0.061	14.2	*Vmn1r66*
10356577	0.0157	0.061	11.9	*Ilkap*
10558961	0.0447	0.061	13.2	*Tspan4*
10388938	0.0446	0.061	5.6	*Wsb1*
10546805	0.0365	0.061	23.8	*Ddx18*
10482109	0.0210	0.061	13.4	*Rbm18*
10389680	0.0054	0.061	15.2	*Msi2*
10502214	0.0410	0.061	14.5	*Cyp2u1*
10601314	0.0403	0.061	16	*Tsx*
10574033	0.0391	0.061	10.9	*Nup93*
10338030	0.0004	0.061	15.4	---
10424066	0.0403	0.061	15.7	*Gm25007*
10386665	0.0338	0.061	1.5	*Slc47a2*
10516081	0.0318	0.061	15.2	*Ppie*

10374223	0.0342	0.061	4.4	*Igfbp1*
10598103	0.0394	0.061	3.7	*Rpl23a*
10601819	0.0277	0.061	7.1	*Armcx5*
10407038	0.0141	0.061	18.6	*Gm10739*
10554475	0.0346	0.061	13.3	*Zfp592*
10470388	0.0439	0.061	10.7	*Cacfd1*
10338015	0.0007	0.061	15.4	---
10338064	0.0020	0.061	15.4	---
10417617	0.0002	0.061	12.5	*Gm12070*
10481474	0.0374	0.061	10.8	*Crat*
10409170	0.0391	0.061	4.8	*Fgd3*
10389522	0.0374	0.060	6.4	*Rpl13a*
10568078	0.0031	0.060	15.2	*Taok2*
10590245	0.0307	0.060	13.9	*Slc25a38*
10466573	0.0164	0.060	6.5	*Ostf1*
10373616	0.0234	0.060	2.6	*Olfr770*
10559420	0.0197	0.060	24	*Tmc4*
10535331	0.0141	0.060	4.8	*Mmd2*
10405916	0.0275	0.060	6.6	*Zfp87*
10559241	0.0419	0.060	23.4	*Igf2os*
10554693	0.0109	0.060	18.2	*Stard5*
10556940	0.0041	0.060	6	*Uqcrc2*
10338043	0.0001	0.060	15.9	---
10549714	0.0035	0.060	5.2	*Rpl28*
10397792	0.0275	0.060	14.9	*Catsperb*
10473517	0.0479	0.060	14.4	*Gm13735*
10552418	0.0364	0.060	8.7	*Etfb*
10516466	0.0219	0.060	20.5	*Zmym1*
10461934	0.0390	0.060	17.4	*Trpm6*
10383932	0.0313	0.060	14.2	*Gas2l1*
10470584	0.0249	0.060	13.8	*Tsc1*
10583441	0.0095	0.060	15.6	*Olfr872*
10338054	0.0354	0.060	15	---
10494296	0.0304	0.060	5.8	*Rps10*
10472994	0.0448	0.060	7.4	*Mtx2*
10394394	0.0413	0.060	16.5	*Apob*
10419759	0.0276	0.060	12.8	*Prmt5*
10433953	0.0097	0.060	2.2	*Ypel1*
10394186	0.0193	0.060	12.6	*Dtnb*
10465226	0.0281	0.060	13.5	*Scyl1*
10599416	0.0003	0.059	15.1	*Gm10483*
10552356	0.0362	0.059	16.1	*Gm10351*
10408926	0.0021	0.059	15.7	*Gm23104*
10342118	0.0134	0.059	12.3	---
10587368	0.0270	0.059	5.1	*Mto1*
10373367	0.0417	0.059	11.4	*Coq10a*
10485170	0.0361	0.059	13.9	*Cry2*
10530278	0.0398	0.059	16.6	---

10450344	0.0160	0.059	18.5	*C2*
10548455	0.0009	0.059	14.4	*Klra1*
10575750	0.0301	0.059	19.7	*Bcmo1*
10374998	0.0196	0.059	14.7	*Gpr75*
10376721	0.0271	0.059	4.9	*Smcr8*
10403948	0.0232	0.059	18.8	*Hist1h2bc*
10441038	0.0258	0.059	13.9	*Hlcs*
10480032	0.0001	0.059	12.2	*Gm12070*
10491967	0.0001	0.059	12.2	*Gm12070*
10496626	0.0001	0.059	12.2	*Gm12070*
10501857	0.0001	0.059	12.2	*Gm12070*
10545765	0.0001	0.059	12.2	*Gm12070*
10529732	0.0299	0.059	5.8	*Hs3st1*
10404717	0.0072	0.059	5.7	*Pak1ip1*
10400254	0.0141	0.059	12.2	*Heatr5a*
10587733	0.0176	0.059	1.4	*Ctsh*
10559649	0.0254	0.059	14.9	*Cox6b2*
10559790	0.0499	0.059	17.5	*Zim1*
10537770	0.0018	0.059	13.6	*Zyx*
10346799	0.0204	0.059	6	*Icos*
10554231	0.0422	0.059	15.5	*Mir7-2*
10550574	0.0310	0.059	16	*Dmpk*
10565921	0.0001	0.058	12.1	*Gm12070*
10485151	0.0098	0.058	7.5	*Mapk8ip1*
10437627	0.0095	0.058	4	*Rpl35a*
10599886	0.0335	0.058	16	*Gm23000*
10406854	0.0269	0.058	3.6	*Rpl27a*
10520527	0.0106	0.058	18.6	*Dpysl5*
10548143	0.0001	0.058	12	*Gm12070*
10376474	0.0351	0.058	3.2	*Mrpl55*
10507105	0.0496	0.058	21.6	*Gm12830*
10338065	0.0087	0.058	15.8	---
10356240	0.0175	0.058	17.4	*Slc16a14*
10486061	0.0141	0.058	15.1	*Rasgrp1*
10473244	0.0190	0.058	13.4	*Zfp804a*
10410625	0.0383	0.058	5.2	*Sdha*
10338050	0.0004	0.058	15.8	---
10338013	0.0017	0.058	16.1	---
10517373	0.0168	0.058	11.4	*Rcan3*
10498529	0.0429	0.058	12.1	*Gm25755*
10597969	0.0295	0.058	6.8	*Hnrnpa1*
10338066	0.0065	0.058	14.7	---
10469255	0.0256	0.058	5.3	*Prkcq*
10393887	0.0312	0.058	3.6	*Pycr1*
10590407	0.0400	0.057	19.7	*Zfp651*
10381603	0.0175	0.057	13.3	*Fzd2*
10584634	0.0018	0.057	13.2	*Usp2*
10449370	0.0356	0.057	5.7	*Rps10*

10382565	0.0050	0.057	6.3	*Mrps7*
10532720	0.0404	0.057	10.4	*Sart3*
10557992	0.0314	0.057	1	*Bag3*
10348963	0.0338	0.057	16.9	*Cntnap5b*
10356677	0.0380	0.057	9.8	*Myeov2*
10378802	0.0233	0.057	12.5	*Blmh*
10423346	0.0262	0.057	8.9	*Zfp622*
10561842	0.0331	0.057	4.6	*Capns1*
10512024	0.0001	0.057	2.3	*Mob3b*
10601343	0.0286	0.057	5.9	*Magee1*
10526181	0.0223	0.057	12.7	*Gatsl2*
10395005	0.0171	0.057	4.5	*Kidins220*
10538629	0.0296	0.057	5	*Rps15*
10343086	0.0012	0.057	13.2	---
10420488	0.0200	0.057	18	*Phf11d*
10456995	0.0368	0.057	6.8	*Txnl4a*
10519264	0.0367	0.056	17.9	*Mir429*
10374315	0.0372	0.056	16.6	*Vwc2*
10572456	0.0484	0.056	5.2	*Jund*
10347232	0.0377	0.056	10	*Xrcc5*
10347115	0.0148	0.056	16.4	*Gm26010*
10412943	0.0468	0.056	15.4	*Nudt13*
10590892	0.0495	0.056	10	*Cep57*
10515072	0.0079	0.056	8.5	*Rnf11*
10378420	0.0356	0.056	15.8	*Zfp616*
10516275	0.0403	0.056	7.5	*Rpl28*
10429178	0.0037	0.056	19.9	*Zfat*
10417920	0.0357	0.056	16.1	*Usp54*
10514000	0.0447	0.056	23.4	*Mpdz*
10410460	0.0248	0.056	8.3	*Ube2ql1*
10393379	0.0173	0.056	3.8	*Mxra7*
10504692	0.0464	0.056	7	*Tmod1*
10580590	0.0001	0.056	12.2	*Gm12070*
10593913	0.0477	0.056	15	*1700017B05Rik*
10549473	0.0096	0.056	16.8	*Caprin2*
10511149	0.0274	0.056	11.7	*Mrpl20*
10371987	0.0301	0.056	9	*Metap2*
10348004	0.0197	0.056	10.2	*Psmd1*
10471191	0.0259	0.056	13.7	*Exosc2*
10528167	0.0001	0.056	11.9	*Gm12070*
10476648	0.0480	0.056	7.3	*Dstn*
10380773	0.0033	0.055	7.3	*Arhgap23*
10521136	0.0390	0.055	7	*Whsc1*
10447483	0.0355	0.055	8.4	*Nanp*
10373594	0.0466	0.055	10.6	*Bloc1s1*
10508454	0.0405	0.055	11.3	*Bsdc1*
10427816	0.0092	0.055	19.5	*Pdzd2*
10345509	0.0336	0.055	3.4	*Zap70*

10528546	0.0315	0.055	5.5	*Gabarapl2*
10441646	0.0308	0.055	5.6	*Sft2d1*
10453562	0.0308	0.055	5.6	*Sft2d1*
10466400	0.0102	0.055	13.5	*Gm26026*
10414078	0.0363	0.055	3.5	*Rpl23a*
10417813	0.0425	0.055	17.4	*Ecd*
10516605	0.0238	0.055	7.1	*Hdac1*
10463005	0.0136	0.055	15.9	*Cyp2c55*
10596925	0.0335	0.055	7.5	*Ndufaf3*
10355173	0.0149	0.055	5.9	*Rpl10a*
10524568	0.0373	0.055	17.6	*Fam222a*
10508253	0.0332	0.055	15.8	*Dlgap3*
10408210	0.0304	0.055	18.5	*Hist1h2bc*
10377534	0.0072	0.055	8.4	*Chd3os*
10526726	0.0483	0.055	15.3	*Zkscan1*
10421707	0.0322	0.055	3.7	*Rpl23a*
10367337	0.0161	0.055	14.7	*Rnf41*
10385542	0.0022	0.055	20.7	*Btnl9*
10566438	0.0004	0.054	4.5	*Fam160a2*
10450089	0.0166	0.054	4.6	*Ring1*
10447729	0.0048	0.054	11.9	*Qk*
10391301	0.0249	0.054	21.2	*Stat3*
10441093	0.0117	0.054	17.8	*Erg*
10520638	0.0382	0.054	11.7	*Atraid*
10582811	0.0394	0.054	1	*Irf2bp2*
10590972	0.0315	0.054	9.4	*Mif*
10553956	0.0052	0.054	8.7	*Tm2d3*
10360970	0.0369	0.054	15.7	---
10481011	0.0464	0.054	7.9	*Inpp5e*
10430127	0.0247	0.054	9.8	*Zfp251*
10594460	0.0192	0.054	5.9	*Dis3l*
10511136	0.0337	0.054	9.1	*B930041F14Rik*
10496387	0.0427	0.054	7.5	*Dnajb14*
10568109	0.0339	0.054	16.3	*Asphd1*
10484912	0.0128	0.054	5.6	*Ndufs3*
10376292	0.0334	0.053	13.6	*Larp1*
10367973	0.0135	0.053	7.1	*Aig1*
10511894	0.0023	0.053	15.4	*Gm26254*
10402294	0.0042	0.053	8.4	*Gm20604*
10526493	0.0363	0.053	1.7	*Myl10*
10527229	0.0376	0.053	9	*Fam220a*
10601888	0.0273	0.053	3.9	*Plp1*
10434148	0.0307	0.053	12.7	*Trmt2a*
10600210	0.0193	0.053	11.5	*Slc6a8*
10582823	0.0186	0.053	13.5	*Rbm34*
10535936	0.0465	0.053	16.4	*Gm25284*
10377431	0.0181	0.053	10.5	*Vamp2*
10554017	0.0109	0.053	22	*Lins*

10341829	0.0026	0.053	11.5	---
10516823	0.0432	0.053	15.8	*Epb4.1*
10380732	0.0236	0.053	10.6	*Mrpl10*
10468909	0.0254	0.053	12.4	*Disp1*
10411171	0.0083	0.053	15.9	*Pde8b*
10359571	0.0440	0.052	16.9	*Fmo1*
10348493	0.0310	0.052	13.3	*Lrrfip1*
10551872	0.0184	0.052	21.1	*Syne4*
10375360	0.0421	0.052	8.9	*Ebf1*
10561799	0.0460	0.052	3.3	*Zfp74*
10394676	0.0182	0.052	16.3	*Gm25414*
10545109	0.0198	0.052	13.8	*Gm23625*
10603009	0.0302	0.052	5	*Uba52*
10439790	0.0286	0.052	15.4	*Trat1*
10440964	0.0451	0.052	5.1	*Cryzl1*
10526179	0.0111	0.052	5.2	*Rpl28*
10561323	0.0233	0.052	13.8	*Map3k10*
10355197	0.0143	0.052	12.8	---
10388776	0.0485	0.052	4.4	*Rpl23a*
10447975	0.0263	0.052	14.8	*Gm23352*
10362823	0.0271	0.052	4.4	*Snx3*
10475866	0.0342	0.052	5	*Bcl2l11*
10526508	0.0061	0.052	5.1	*Fis1*
10525187	0.0375	0.052	7.3	*Gm5428*
10403202	0.0489	0.051	9.3	*Abcb5*
10396306	0.0151	0.051	10.7	*Jkamp*
10369748	0.0357	0.051	23.1	*Gm7075*
10511342	0.0174	0.051	4.8	*Uba52*
10363460	0.0086	0.051	16.9	*Tbata*
10361816	0.0275	0.051	3.8	*Rpl23a*
10601404	0.0472	0.051	20.5	*Gm5127*
10573160	0.0198	0.051	14.5	*4933434I20Rik*
10386171	0.0241	0.051	7	*Ndufs3*
10435094	0.0467	0.051	2.8	*Tnk2*
10589913	0.0303	0.051	10.7	*Dync1li1*
10498155	0.0022	0.051	3.2	*Rps14*
10429666	0.0399	0.051	19.2	*Pycrl*
10517517	0.0337	0.051	4.5	*C1qa*
10538082	0.0006	0.051	5.7	*Atp6v0e2*
10387757	0.0368	0.051	6	*Elp5*
10453600	0.0499	0.051	8.8	---
10458731	0.0140	0.051	14.4	*Mcc*
10384552	0.0009	0.050	11.5	*Gm12070*
10523354	0.0009	0.050	11.5	*Gm12070*
10532027	0.0009	0.050	11.5	*Gm12070*
10561085	0.0264	0.050	10	*Hnrnpul1*
10579302	0.0278	0.050	5.3	*Uba52*
10503845	0.0363	0.050	9.6	*Ube2j1*

10390430	0.0109	0.050	14.1	*Srcin1*
10512774	0.0302	0.050	16.6	*Coro2a*
10359375	0.0180	0.050	19.3	*Gpr52*
10399308	0.0166	0.050	8.5	*Fkbp1b*
10338001	0.0000	0.050	1.1	---
10555662	0.0484	0.050	24	*Art1*
10454881	0.0476	0.050	5.6	*Cystm1*
10443360	0.0333	0.050	6.4	*Rpl10a*
10482766	0.0450	0.050	21.8	*Rprm*
10501319	0.0308	0.050	7.4	*Celsr2*
10581495	0.0223	0.050	7.9	*Rplp1*
10551496	0.0446	0.050	14.4	*Paf1*
10577604	0.0429	0.050	13.9	*Agpat6*
10371959	0.0267	0.050	4	*Elk3*
10552121	0.0162	0.050	19.3	*Scgb1b27*
10544089	0.0350	0.050	15.5	*Zc3hav1*
10604932	0.0219	0.050	7	*Cd99l2*
10572906	0.0242	0.050	20.2	*Mcm5*
10590646	0.0262	0.049	18.8	---
10482089	0.0114	0.049	4.8	*Ndufa8*
10511156	0.0170	0.049	14.7	*Ccnl2*
10427310	0.0255	0.049	6.8	*Hnrnpa1*
10559676	0.0135	0.049	6.8	*Ube2s*
10391518	0.0099	0.049	12	*Mpp3*
10477061	0.0028	0.049	5.9	*Srxn1*
10432398	0.0172	0.049	4.5	*Tuba1c*
10548541	0.0275	0.049	16.4	*Gm20997*
10400926	0.0362	0.049	6.4	*Rtn1*
10375704	0.0315	0.049	5.2	*3010026O09Rik*
10469559	0.0362	0.049	9.7	*Msrb2*
10359136	0.0189	0.049	15.4	*Axdnd1*
10383564	0.0438	0.048	2.1	*Fn3k*
10391854	0.0432	0.048	14.5	*1700023F06Rik*
10470109	0.0314	0.048	7.7	*Fbxw5*
10482273	0.0381	0.048	18.1	*Golga1*
10505064	0.0276	0.048	19.4	*Tmem38b*
10438672	0.0463	0.048	13.4	*Tbccd1*
10515696	0.0012	0.048	15.9	*Szt2*
10546702	0.0314	0.048	4.8	*Tpt1*
10366476	0.0250	0.048	13.9	*Ptprb*
10536563	0.0463	0.048	22.9	*Cftr*
10356351	0.0277	0.048	7	*Gm5428*
10340862	0.0417	0.048	5.5	---
10511269	0.0369	0.048	6.6	*Sdf4*
10534301	0.0307	0.048	12	*Syna*
10356601	0.0322	0.048	15	*Per2*
10585586	0.0066	0.047	7.2	*Ube2s*
10474669	0.0306	0.047	4.2	*Rpl27a*

10566502	0.0232	0.047	15.4	*Arfip2*
10574246	0.0092	0.047	18.8	*Gpr114*
10381647	0.0149	0.047	17.5	*Gm22683*
10579769	0.0001	0.047	12.2	*Gm12070*
10466970	0.0349	0.047	10.9	*9930021J03Rik*
10353413	0.0137	0.047	18.6	*Gm24162*
10422161	0.0005	0.047	11.4	*Gm12070*
10411804	0.0382	0.047	18.4	*Mast4*
10411452	0.0011	0.047	10.2	*Gm12070*
10425726	0.0218	0.046	9.9	*Sep 03*
10571248	0.0265	0.046	23	---
10397002	0.0089	0.046	5	*Sipa1l1*
10433660	0.0464	0.046	4.3	*3110001I22Rik*
10338060	0.0059	0.046	15.4	---
10350990	0.0303	0.046	3.9	*Rpl23a*
10463930	0.0390	0.046	16.5	*Mxi1*
10415282	0.0407	0.046	12.7	*Psme1*
10601569	0.0129	0.046	11.2	*Pcdh11x*
10426650	0.0417	0.045	4.5	*Tuba1c*
10455238	0.0393	0.045	6.8	*Ndfip1*
10598721	0.0319	0.045	6.5	*Rpl3*
10589723	0.0234	0.045	14.9	*Lrrfip2*
10589061	0.0418	0.045	9.3	*Dalrd3*
10371107	0.0350	0.045	12.4	*Mrpl54*
10387372	0.0361	0.045	16.7	*Kdm6b*
10584350	0.0321	0.044	4.6	*Tpt1*
10416496	0.0434	0.044	4.8	*Tpt1*
10373407	0.0143	0.044	2.6	*Esyt1*
10478124	0.0089	0.044	8	*Slc32a1*
10410063	0.0390	0.044	5.7	*Rpl10a*
10398267	0.0038	0.044	13.1	*Evl*
10435581	0.0276	0.044	2.2	*Polq*
10530142	0.0469	0.044	17.1	---
10545538	0.0325	0.044	10.2	*Ctnna2*
10349478	0.0256	0.044	5.2	*Rpl28*
10549615	0.0473	0.044	10.1	*Leng8*
10430058	0.0195	0.044	11.9	*Cyhr1*
10434436	0.0371	0.043	3.5	*Vwa5b2*
10452415	0.0005	0.043	11.8	*Gm12070*
10557758	0.0308	0.043	8	*Setd1a*
10567591	0.0469	0.043	21.1	*Usp31*
10448204	0.0462	0.043	12.5	*Gm9772*
10338047	0.0133	0.043	16.1	---
10547869	0.0150	0.042	6.8	*Leprel2*
10579307	0.0259	0.042	12.7	*Kxd1*
10338056	0.0040	0.042	15.2	---
10360648	0.0240	0.042	3.7	*Psen2*
10441396	0.0353	0.042	6.2	*Ppia*

10377593	0.0219	0.041	13.8	*Zbtb4*
10444756	0.0107	0.041	5.8	*Atp6v1g2*
10468517	0.0283	0.041	16.8	*Gm10197*
10516507	0.0357	0.041	21.7	*Zscan20*
10378154	0.0215	0.041	18.5	*Zzef1*
10338037	0.0031	0.041	15.6	---
10608690	0.0028	0.040	12	---
10353250	0.0017	0.040	10.6	*Gm12070*
10390237	0.0288	0.040	8.9	*Atp5g1*
10504316	0.0483	0.040	9.8	*Tesk1*
10471677	0.0209	0.039	11.2	*Dab2ip*
10447699	0.0081	0.038	13	*Gm12070*
10579996	0.0405	0.038	7.1	*Tecr*
10445565	0.0372	0.037	11.6	*Mrpl2*
10379795	0.0436	0.037	13.4	*Synrg*
10388896	0.0388	0.037	11.6	*Gm11197*
10382271	0.0424	0.036	8.9	*Arsg*
10373519	0.0412	0.036	4.5	*LOC102635048*
10438959	0.0418	0.035	12.1	*Lsg1*
10512315	0.0367	0.035	10	*Ccl27b*
10489484	0.0154	0.032	16.1	*Sdc4*
10469197	0.0179	0.031	18.9	*Mir669p-2*
10469201	0.0179	0.031	18.9	*Mir669p-2*
10469205	0.0179	0.031	18.9	*Mir669p-2*
10469209	0.0179	0.031	18.9	*Mir669p-2*
10469211	0.0179	0.031	18.9	*Mir669p-2*
10469215	0.0179	0.031	18.9	*Mir669p-2*
10469219	0.0179	0.031	18.9	*Mir669p-2*
10469223	0.0179	0.031	18.9	*Mir669p-2*
10469229	0.0179	0.031	18.9	*Mir669p-2*
10469233	0.0179	0.031	18.9	*Mir669p-2*
10469235	0.0179	0.031	18.9	*Mir669p-2*
10469241	0.0179	0.031	18.9	*Mir669p-2*
10469245	0.0179	0.031	18.9	*Mir669p-2*
10598025	0.0214	0.029	11.5	*mt-Rnr2*
10462039	0.0059	0.027	24	*Trpm3*
10549282	0.0376	0.027	18	*Itpr2*
10338059	0.0001	0.023	1.2	---
