# Supplementary material for: Circadian regulation of hedonic appetite in mice by clocks in dopaminergic neurons of the VTA
Source: Nat Commun. 2020 Jun 17;11:3071. doi: 10.1038/s41467-020-16882-6 (PMC7299974; doi:10.1038/s41467-020-16882-6)
Supplement: Supplementary file 2 — Peer Review File [file 41467_2020_16882_MOESM2_ESM.docx]

Peer Review Information

**Journal:** Nature Communications

**Manuscript title:** Circadian regulation of hedonic appetite in mice by clocks in dopaminergic neurons of the VTA

**Corresponding author name(s):** Prof Henrik Oster

**Editorial notes:**

**Reviewer comments & decisions:**

**Reviewer comments, first version:**

Reviewers' comments:

Reviewer #2 (Remarks to the Author):

Koch et al. use a series of behavioral and genetic approaches to demonstrate that food consumption driven by hedonic properties differs from homeostatic food consumption with respect to circadian rhythms. The authors show that in mice with modifications to genes associated with circadian cycles (Per1/2 and Bmal1), this pattern of rhythmic feeding is disrupted. They then go on to argue that the rhythmic pattern of hedonic overconsumption is driven by Bmal1 expression within the VTA. While the questions being addressed are important, the experiments used in this study offer little novel insight into the underlying biology. I have many concerns that preclude publication of the manuscript in its current form.

Major concerns:

The inclusion of ‘dopaminergic’ in the title is not justified, and the relationship between the present results and dopamine is overstated as it is currently written. The only direct measurement of dopamine is in Fig. 5e, and this fails to reach statistical significance. The rest of the dopamine-related data rely on tyrosine hydroxylase as a proxy for dopaminergic neurons, and should be discussed as such.

Why is Per1/2 used as the comparison group for Fig. 4d, but then causal manipulations in Fig. 5 are focused on Bmal1? The disconnect between these figures should be addressed.

Is the rhythmic clock gene expression within the VTA altered in Bmal1-KO mice? Since these mice also have altered hedonic overconsumption patterns (Fig. 3d), it would stand to reason that their circadian mRNA expression would also be disrupted.

Figure 3a and 3b seem to contradict the authors’ claims that hedonic overconsumption during the rest phase is controlled by circadian cues. The pattern of overconsumption is the roughly the same regardless of whether mice are in DD conditions or not (comparing 2c and 3b).

Methodological details are unclear at many points in the manuscript. Specifically for Fig. 4, how are the data for different timepoints collected? According to the methods, mice were sacrificed every six hours for 24 hours, yet the figures seem indicate the presence of many more time points (~40 in 4a, 24 in 4b, and 8 in 4d). This should be clearly explained in both the main text and the figure legends. Why are data in 4d “double plotted”? It is unclear from the description of the statistics how the comparisons were made, but any comparisons should be made on only one copy of the data and double plotting should be removed. In general, the methods for collecting, processing, analyzing, and presenting the data in Fig. 4 are unclear. As it is currently written, it is unclear from the figure legend whether data in Fig. 4 came from repositories or were collected as part of this study.

What are the control mice used for viral injection experiments (Fig. 5)? Histological validation of Cre-recombinase or Bmal1 expression restricted to the VTA should be included. In addition, the lack of expression should be demonstrated in control mice. Demonstrating the efficacy and specificity of the viral knockdown strategy is especially important for the TH-specific Bmal1 knockdown experiment (Fig. 5f-h).

The statistics are insufficiently described throughout. Specifically, the meaning of the letters indicating statistical significance in 2b and 2c is unclear. Exactly what comparisons does each letter correspond to? The use of one-way ANOVAs instead of two-way ANOVAs in Fig. 2c should be justified or corrected. In 2c, the critical comparison for the authors’ argument seems to be whether hedonic overconsumption is significantly different between timepoints within the same group. For example, was chocolate+chow consumption during the rest phase increased relative to chocolate+chow consumption during the active phase? This should be directly addressed in the results, as the current description of the results focuses only on elevation of intake relative to homeostatic baseline conditions.

Minor concerns:

The legend for Fig. 2d should be added to indicate that the colored bars reflect preference for chocolate, sucrose, or sucralose rather than chocolate+chow, sucrose+water, and sucralose+water.

Are the metabolic phenotypes (e.g., temperature, body weight, energy expenditure) of Per1/2 and Bmal1-KO mice similar to those of the WT controls? Additionally, is hedonic choice (i.e., sucrose or chocolate preference) altered independently of changes in circadian rhythm?

The claim that “TH expression in the VTA correlates with hedonic appetite regulation in WT and clock-deficient mice” (p. 8) should be justified with the appropriate analysis and presentation or else the statement should be removed.

Was nucleus accumbens DOPAC altered in the VTA-Bmal1-KD mice (similar to the pattern observed in Fig. 5e)?

p. 5: How can the measurement food intake in the choice paradigm be considered ‘memory-independent”? One would assume that experience with the hedonic (chocolate, sucrose, sucralose) stimuli would influence future behavior in a learned fashion even within a single experiment.

p 7: reference to Fig. 4b is incorrect.


Reviewer #3 (Remarks to the Author):

NCOMMS-19-03435
Dopaminergic regulation of circadian feeding rhythms in mice

Koch CE, Kiehn JT, Griewahn L, Begemann K, Mauer J, Hess ME, Moser A, Schmid SM, Bruning JC, Oster H.

In this study, the authors nicely show that homeostatic and hedonic feeding not only involves the control by different brain areas, but they also show a different daily rhythmicity. Whereas the drive for homeostatic feeding is strongest after waking up at the start of the activity phase, an increased sensitivity to palatable food is found during the rest phase. Moreover, they provided evidence that the hedonic feeding rhythm is driven by dopaminergic neurons in the ventral tegmental area (VTA). Not only did the authors show a clear daily rhythmicity in the activity of these dopaminergic neurons, they also showed that an arrest of the molecular clock in these neurons disrupts the daily rhythm in hedonic feeding, but leaves intact the rhythm in homeostatic feeding.
The authors conclude that the identification of this function for the dopaminergic VTA neurons may also provide new therapeutic targets to counteract hyperphagia in an obesogenic environment.

Methodological remarks
- Why did the authors use both a sucrose and a sucralose group? The rationale for the sucralose group is not mentioned or discussed.
- What is the conclusion of the authors about the possible mechanism involved in hedonic feeding based on the similar results for the sucrose and sucralose group?
- What is the conclusion of the authors about the possible mechanism involved in hedonic feeding based on the timing differences between chocolate and sucrose/sucralose?
- Based on the last sentence on page 5, i.e., no circadian variation of sweet taste, shouldn’t the conclusion be that there is no anti-phasic homeostatic and hedonic feeding rhythm, but only a homeostatic feeding rhythm? In fact, the daily rhythm in overconsumption is only and totally due to the daily rhythm in homeostatic feeding.
- Why do the clock knock-out animals, Per1/2 double-KO and Bmal1-KO, still display daily rhythms in feeding activity? Is this masking by the L/D-cycle?
- How are the two transcript clusters shown in fig.4b related to homeostatic and hedonic feeding?
- The experiment with the crossing of the Bmal1flx/flx mice with the CRE-dopamine transporter mice should be removed from the manuscript. Not only did this experiment not produce any useful data, but also in case of a correct response of the control mice the results of this experiment would not have added any specificity to the data produced by the Bmal1flx/flx mice and VTA injections, since all dopaminergic neurons are affected, not only the ones in the VTA.
- Finally, why would homeostatic and hedonic feeding have an opposite daily rhythm? At the end of the Discussion the authors argue that from an evolutionary perspective it might be beneficial as much high-energy food as possible at the beginning of the rest phase for energy storage, but why wouldn’t the same argument hold for less energy-dense foods?
- The authors clearly show that DA activity in the VTA is rhythmic. However, more important for their line of reasoning is whether DA release in the VTA target areas is rhythmic. The only information regarding DA “release” is presented in fig.5e, showing lower DA concentrations in the morning in the nucleus accumbens of the VTA-Bmal1-KD mice as compared to controls. The authors should also present the daily variation of DA concentrations in the nucleus accumbens of control animals and preferably also some additional VTA target areas such as the prefrontal cortex and the amygdala.

Additional remarks
- The 2nd and 3rd time fig.4b is mentioned on page 7, this should be fig.4c
- In fig.5 it is unclear to me how overconsumption can change (fig.5g) in the experimental group, if chow intake (fig.5f) and chocolate preference (fig.5h) are not changing?
- With regard to the above, it is confusing that no data are provided on the absolute intake of palatable food or drinking, only absolute data on regular chow and water intake are available. This also means it is not clear whether the interventions affect the absolute intake of the palatable food or only its rhythm.
- Why is overall caloric intake increased in DD conditions (fig.S1)?

**Author rebuttal, first version:**

Reviewer #2:

Koch et al. use a series of behavioral and genetic approaches to demonstrate that food consumption driven by hedonic properties differs from homeostatic food consumption with respect to circadian rhythms. The authors show that in mice with modifications to genes associated with circadian cycles (Per1/2 and Bmal1), this pattern of rhythmic feeding is disrupted. They then go on to argue that the rhythmic pattern of hedonic overconsumption is driven by Bmal1 expression within the VTA. While the questions being addressed are important, the experiments used in this study offer little novel insight into the underlying biology. I have many concerns that preclude publication of the manuscript in its current form.

Major concerns:

4. The inclusion of ‘dopaminergic’ in the title is not justified, and the relationship between the present results and dopamine is overstated as it is currently written. The only direct measurement of dopamine is in Fig. 5e, and this fails to reach statistical significance. The rest of the dopamine-related data rely on tyrosine hydroxylase as a proxy for dopaminergic neurons, and should be discussed as such.

*Reply: We thank the reviewer for this comment. We now provide more data on the role of dopamine in the observed hedonic overconsumption rhythms. Nevertheless, we cannot conclusively show that dopamine is driving the observed rhythms in overconsumption under chow conditions. We modified the title accordingly, reflecting that we primarily address the role of VTA clocks (and clocks in VTA dopaminergic neurons) in this regulation. Finally, we have modified the wording throughout the ms to better reflect our findings.*

5. Why is Per1/2 used as the comparison group for Fig. 4d, but then causal manipulations in Fig. 5 are focused on Bmal1? The disconnect between these figures should be addressed.

*Reply: We have added Bmal1-KO data to Fig. 4. Similar to Per1/2 mutants these mice show arrhythmic VTY mRNA expression levels and low Th expression.*

6. Is the rhythmic clock gene expression within the VTA altered in Bmal1-KO mice? Since these mice also have altered hedonic overconsumption patterns (Fig. 3d), it would stand to reason that their circadian mRNA expression would also be disrupted.

*Reply: pls. see answer above. We added these data to Figure 4.*

7. Figure 3a and 3b seem to contradict the authors’ claims that hedonic overconsumption during the rest phase is controlled by circadian cues. The pattern of overconsumption is the roughly the same regardless of whether mice are in DD conditions or not (comparing 2c and 3b).

*Reply: We are sorry that our wording was confusing here. We actually think that the fact that LD and DD data are very comparable supports our conclusion that the observed rhythms are regulated by an endogenous circadian timing system (and not an external factor such as the light-dark cycle). We revised the corresponding section to, hopefully, make this clearer.*

8. Methodological details are unclear at many points in the manuscript. Specifically for Fig. 4, how are the data for different timepoints collected? According to the methods, mice were sacrificed every six hours for 24 hours, yet the figures seem indicate the presence of many more time points (~40 in 4a, 24 in 4b, and 8 in 4d). This should be clearly explained in both the main text and the figure legends. Why are data in 4d “double plotted”? It is unclear from the description of the statistics how the comparisons were made, but any comparisons should be made on only one copy of the data and double plotting should be removed. In general, the methods for collecting, processing, analyzing, and presenting the data in Fig. 4 are unclear. As it is currently written, it is unclear from the figure legend whether data in Fig. 4 came from repositories or were collected as part of this study.

*Reply: We thank the reviewer for these comments. We have re-plotted the data omitting the double plot view. This should also clarify the confusing statistics. All data in Figure 4 were collected as part of this study. We updated the methods section to make this clearer.*

9. What are the control mice used for viral injection experiments (Fig. 5)? Histological validation of Cre-recombinase or Bmal1 expression restricted to the VTA should be included. In addition, the lack of expression should be demonstrated in control mice. Demonstrating the efficacy and specificity of the viral knockdown strategy is especially important for the TH-specific Bmal1 knockdown experiment (Fig. 5f-h).

*Reply: We now include a more detailed description for the control injections (scramble AAV) in the Methods and the Figure. We also provide data for virus-driven expression of GFP (VTA) or CRE-mediated recombination (DA neurons) in situ and from in laser capture dissections of the VTA and the NAc and the mediobasal hypothalamus (as control regions) (Fig. 5, S3 & S4).*

10. The statistics are insufficiently described throughout. Specifically, the meaning of the letters indicating statistical significance in 2b and 2c is unclear. Exactly what comparisons does each letter correspond to? The use of one-way ANOVAs instead of two-way ANOVAs in Fig. 2c should be justified or corrected. In 2c, the critical comparison for the authors’ argument seems to be whether hedonic overconsumption is significantly different between timepoints within the same group. For example, was chocolate+chow consumption during the rest phase increased relative to chocolate+chow consumption during the active phase? This should be directly addressed in the results, as the current description of the results focuses only on elevation of intake relative to homeostatic baseline conditions.

*Reply: We apologize for this confusion. We now in more detail describe the statistics for Figure 2. In line with the argument of the reviewer, we now use CircWave cosine analysis to test for 24-h rhythmicity in chow and overconsumption time courses (Figs. 2, 3, 5 – and for gene expression in Fig. 4). 2-way ANOVAs with post-tests were used for time point comparisons between different genotypes (Figs. 3-5). The confusing letter labeling was removed.*

*Regarding the idea that rhythms in hedonic overconsumption should be determined by comparing absolute consumption of chow and chocolate at the different time point, we beg to differ. Chocolate/sucrose intake is simultaneously regulated by both homeostatic and hedonic factors and, thus, we think that overconsumption should be weighed against chow/water intake under non-choice conditions to reflect the vulnerability of the system to a hedonic stimulus under specific homeostatic baseline conditions. In fact, raw chocolate (or sucrose) consumption is usually higher during the activity phase under both choice and non-choice conditions – driven to a substantial part by homeostatic appetite. The fact that these circadian changes in relative overconsumption are blunted (to a much higher degree than homeostatic intake rhythms) when the clock system is disrupted and that place preference (which does not involve any caloric intake during test days) is regulated the same way further support this idea. We added the raw intake data for most choice experiments (Fig.2c-e, 3b/e, S1c, S3c, S4b). We also changed the methods, results and discussion to better reflect our reasoning for the relative overconsumption readout.*

Minor concerns:

11. The legend for Fig. 2d should be added to indicate that the colored bars reflect preference for chocolate, sucrose, or sucralose rather than chocolate+chow, sucrose+water, and sucralose+water.

*Reply: We have added this information.*

12. Are the metabolic phenotypes (e.g., temperature, body weight, energy expenditure) of Per1/2 and Bmal1-KO mice similar to those of the WT controls? Additionally, is hedonic choice (i.e., sucrose or chocolate preference) altered independently of changes in circadian rhythm?

*Reply: We added references on metabolic phenotypes of Per1/2 and Bmal1-KO mice in the methods section. Importantly, we similar chocolate preferences and total caloric intake in our mutants (Figure S1c which we also now mention in the text.* *A complete metabolic characterization including energy expenditure/oxygen consumption and temperature profiles, we believe, would be beyond the scope of the current study.*

13. The claim that “TH expression in the VTA correlates with hedonic appetite regulation in WT and clock-deficient mice” (p. 8) should be justified with the appropriate analysis and presentation or else the statement should be removed.

*Reply: We agree that TH mRNA expression rhythms are slightly advanced relative to hedonic overconsumption (Figure 2f vs. 4d). We modified this statement accordingly.*

14. Was nucleus accumbens DOPAC altered in the VTA-Bmal1-KD mice (similar to the pattern observed in Fig. 5e)?

*Reply: We added these data (Fig. 5g) and discuss them in the context of the in-vivo microdialysis data we obtained from WT mice.*

15. p. 5: How can the measurement food intake in the choice paradigm be considered ‘memory-independent”? One would assume that experience with the hedonic (chocolate, sucrose, sucralose) stimuli would influence future behavior in a learned fashion even within a single experiment.

*Reply: We thank the reviewer for this notion. We agree that this is not a good argument for the introduction of the food/drink choice experiments. We changed the wording of this sentence.*

16. p 7: reference to Fig. 4b is incorrect.

*Reply: We fixed this.*

Reviewer #3 (Remarks to the Author):

In this study, the authors nicely show that homeostatic and hedonic feeding not only involves the control by different brain areas, but they also show a different daily rhythmicity. Whereas the drive for homeostatic feeding is strongest after waking up at the start of the activity phase, an increased sensitivity to palatable food is found during the rest phase. Moreover, they provided evidence that the hedonic feeding rhythm is driven by dopaminergic neurons in the ventral tegmental area (VTA). Not only did the authors show a clear daily rhythmicity in the activity of these dopaminergic neurons, they also showed that an arrest of the molecular clock in these neurons disrupts the daily rhythm in hedonic feeding, but leaves intact the rhythm in homeostatic feeding. The authors conclude that the identification of this function for the dopaminergic VTA neurons may also provide new therapeutic targets to counteract hyperphagia in an obesogenic environment.

Methodological remarks
17. Why did the authors use both a sucrose and a sucralose group? The rationale for the sucralose group is not mentioned or discussed.

*Reply: We added the sucralose paradigm as a non-calorie dependent test. We added an explanatory sentence to the text.*

18. What is the conclusion of the authors about the possible mechanism involved in hedonic feeding based on the similar results for the sucrose and sucralose group?

*Reply: Along the argument above, we conclude that the hedonic regulation of appetite does not depend on the caloric value of the stimulus but, in this case, would rather reflect sweet taste. We added this statement to the discussion.*

19. What is the conclusion of the authors about the possible mechanism involved in hedonic feeding based on the timing differences between chocolate and sucrose/sucralose?

*Reply: We currently have no strong explanation for these differences in timing. One possibility would be that baseline (homeostatic) rhythms are not exactly the same for food and water intake or that downstream circuits may differ. For example, in Figure 2b, water intake drops towards the end of the night relative to food. Alternatively, some of these apparent differences may result from the coarse sampling interval of 6h used for the food intake studies. In other words, the maximum effect may roughly occur around ZT6 for both readouts, but slight differences in the distribution may change the apparent shape of the profile. We added a statement on this to the discussion.*

20. Based on the last sentence on page 5, i.e., no circadian variation of sweet taste, shouldn’t the conclusion be that there is no anti-phasic homeostatic and hedonic feeding rhythm, but only a homeostatic feeding rhythm? In fact, the daily rhythm in overconsumption is only and totally due to the daily rhythm in homeostatic feeding.

*Reply: We believe that under choice conditions hedonic overconsumption should be normalized against (non-choice) homeostatic intake as chocolate/sugar intake will always be the consequence of, both, homeostatic (energetic) and hedonic (in this case – likely – sweet) regulation. Pls. also refer to our answer to point 10.*

21. Why do the clock knock-out animals, Per1/2 double-KO and Bmal1-KO, still display daily rhythms in feeding activity? Is this masking by the L/D-cycle?

*Reply: Yes, we believe that this is the case as these rhythms are lost immediately upon release into constant darkness (DD). We now mention this in the text.*

22. How are the two transcript clusters shown in fig.4b related to homeostatic and hedonic feeding?

*Reply: We thank the reviewer for this point. We have removed this confusing statement as the phase clustering is just a general description of the distribution of the diurnal VTA transcriptome.*

23. The experiment with the crossing of the Bmal1flx/flx mice with the CRE-dopamine transporter mice should be removed from the manuscript. Not only did this experiment not produce any useful data, but also in case of a correct response of the control mice the results of this experiment would not have added any specificity to the data produced by the Bmal1flx/flx mice and VTA injections, since all dopaminergic neurons are affected, not only the ones in the VTA.

*Reply: We omitted these data.*

24. Finally, why would homeostatic and hedonic feeding have an opposite daily rhythm? At the end of the Discussion the authors argue that from an evolutionary perspective it might be beneficial as much high-energy food as possible at the beginning of the rest phase for energy storage, but why wouldn’t the same argument hold for less energy-dense foods?

*Reply: Thank you for this notion. We think that this should be seen under conditions of choice (which is what we tested) – so our argument would make sense if as much energy-dense food as possible is consumed at the beginning of the rest phase instead of low-energy food. We have modified this in the discussion and hope that this is clearer now.*

25. The authors clearly show that DA activity in the VTA is rhythmic. However, more important for their line of reasoning is whether DA release in the VTA target areas is rhythmic. The only information regarding DA “release” is presented in fig.5e, showing lower DA concentrations in the morning in the nucleus accumbens of the VTA-Bmal1-KD mice as compared to controls. The authors should also present the daily variation of DA concentrations in the nucleus accumbens of control animals and preferably also some additional VTA target areas such as the prefrontal cortex and the amygdala.

*Reply: We have now added in-vivo microdialysis data on DA levels in the NAcc under baseline conditions and after chocolate intake showing a diurnal regulation with elevated baseline levels – but reduced chocolate responses – in the evening (Fig. 2).*

Additional remarks
26. The 2nd and 3rd time fig.4b is mentioned on page 7, this should be fig.4c

*Reply: We have fixed this.*

27. In fig.5 it is unclear to me how overconsumption can change (fig.5g) in the experimental group, if chow intake (fig.5f) and chocolate preference (fig.5h) are not changing?

*Reply: Please note that chow references are different for the overconsumption (chow intake under non-choice conditions) and the preference (chow intake under choice conditions). Also following remark 10 of reviewer 2, we now also show raw chow and chocolate intake data for the choice experiments to make this more transparent. We also now make this clearer in the text.*

28. With regard to the above, it is confusing that no data are provided on the absolute intake of palatable food or drinking, only absolute data on regular chow and water intake are available. This also means it is not clear whether the interventions affect the absolute intake of the palatable food or only its rhythm.

*Reply: We have fixed this - pls. refer to remarks 10 and 27 for details. Absolute data under choice conditions are now shown in Figs. 2, 3, S3, and S4.*

**Reviewer comments, second version:**

Reviewer #2 (Remarks to the Author):

The authors have sufficiently addressed my comments regarding insufficient methodological details and statistical analyses. Overall, the manuscript has been improved by the additional experiments and control data. While I agree with the author’s interpretation of many of the results, a specific relationship between the present data and dopamine signaling is still overstated throughout the Results section. In many places, the authors refer to correlations that were not demonstrated and extrapolate beyond what data are presented when linking their results to dopamine function.

Some examples:

• The link between “anti-phasic circadian regulation of homeostatic and hedonic appetite correlating with diurnal regulation of dopamine responses in the NAc” (p.7) is not directly demonstrated. Showing the amount of chocolate consumed and the relationship to dopamine signaling is critical to support this point.

• Likewise, the statement that “Th expression in the VTA correlates with hedonic overconsumption in WT mice” is not directly supported by the data. This analysis was not performed.

• “…diurnal food choice regulation correlating with changes in DA signaling in the NAc” (p. 10). The data do not directly support this claim.

• “Increased vulnerability to palatable food during the normal rest phase correlated with increased Th expression in the VTA and dopamine responses in the NAc” (p. 11). The data do not directly support this claim.

These statements should either be rephrased to accurately reflect the results, or the appropriate analyses should be included.

Additional comments:

Fig. 4a should have 24 columns (4 mice/timepoint x 6 timepoints). I count 23. Please correct the missing column. As it is now, the lines denoting the timepoints do not correctly align with the columns.

Please include additional methodological details pertaining to the microdialysis experiment. Specifically, how was dopamine induction calculated? Why is the effect of chocolate on dopamine levels (Fig. 2j) shown as dopamine induction and not dopamine concentration? Using the same metric for Figs. 2i and 2j would be helpful to permit comparisons between the two experiments. If two different metrics are to be shown, please reconsider referring to both as “dopamine levels” in the figure legend to avoid confusion. Was chocolate available during the entire sample period in 2j? Did chocolate consumption differ between the two epochs?


Reviewer #3 (Remarks to the Author):

NCOMMS-19-03435-Revised
Circadian regulation of hedonic appetite in mice by clocks in dopaminergic neurons of the ventral tegmentum

Koch CE, Kiehn JT, Griewahn L, Begemann K, Mauer J, Hess ME, Moser A, Schmid SM, Bruning JC, Oster H.

The authors appropriately addressed my initial concerns, I have only a few remaining remarks.

In the abstract the authors mention an increased circadian vulnerability to palatable foods during the “rest phase”, but at the end of the introduction they mention “early rest phase”. It seems “rest phase” is more appropriate since the early rest phase is specific for chocolate intake.
Lines 92-92: Any idea why the daily rhythm in chow intake was not more pronounced?
Lines 111-113: This sentence starts with “Similar to the increased chow-induced place preference during the active phase”, but according to the information in line 92 no significant increase was observed.
Line 129: “Dopamine” does not need a capital here.
Lines 131-134: The authors report a higher responsiveness of the NAc dopamine system during the rest phase. On the other hand, basal dopamine levels are higher during the active phase. The authors should explain why the higher basal levels do not results in a higher intake of palatable food during the active phase.
Lines 163-165: The final sentence of this paragraph can be removed as it is a repeat and not a conclusion based on the information in this paragraph.
Lines 171-173: Why was this experiment not performed under circadian, ie DD, conditions?
Lines 196-197 and line 320: The authors mention that maximal TH expression at the beginning of the rest phase is phase-aligned with a higher hedonic intake. However, this only holds for chocolate intake, as sucrose/sucralose intake is higher during the second halve of the rest phase.
Lines 216-271: Here the authors report reduced VTA TH mRNA levels. Why would these lower TH levels only affect the diurnal rhythmicity of hedonic overconsumption and not overconsumption itself?
Lines 339-344, I think in their conclusion the authors should stress more clearly first, that the current results provide a nice explanation for why a HFD may result in more food intake during the light period and chronodisruption, and second that their findings implicate that the negative health effects of palatable foods are the result of two different mechanisms, not only does it result in hyperphagia and overconsumption, but it also results in feeding at-the-wrong time of day, i.e. chronodisruption. Here, the authors also might want to take into account the recent paper on chocolate for breakfast of Escobar et al., Sci Reports, 2020.
Line 350: Isn’t it more logical to assume such an increased preference at the end of the active phase instead of beginning of the rest phase?

**Author rebuttal, second version:**

***Reviewer #2*** (Remarks to the Author):

The authors have sufficiently addressed my comments regarding insufficient methodological details and statistical analyses. Overall, the manuscript has been improved by the additional experiments and control data. While I agree with the author’s interpretation of many of the results, a specific relationship between the present data and dopamine signalling is still overstated throughout the Results section. In many places, the authors refer to correlations that were not demonstrated and extrapolate beyond what data are presented when linking their results to dopamine function.

***Reply:*** *We thank the reviewer for making this point. We agree that we overstressed the point of lower Th expression supporting the idea that dopamine function may directly control hedonic overeating. We also did not do any formal correlation analyses. We carefully reworded the respective passages to reflect this.*

Some examples:

• The link between “anti-phasic circadian regulation of homeostatic and hedonic appetite correlating with diurnal regulation of dopamine responses in the NAc” (p.7) is not directly demonstrated. Showing the amount of chocolate consumed and the relationship to dopamine signaling is critical to support this point.

***Reply:*** *We have reworded this sentence. It now reads: "In line with an increased hedonic overconsumption during the rest phase under choice conditions chocolate induced dopamine responses in the NAc were elevated in the morning."*

• Likewise, the statement that “Th expression in the VTA correlates with hedonic overconsumption in WT mice” is not directly supported by the data. This analysis was not performed.

***Reply:*** *We have reworded this sentence. It now reads: "Th expression in the VTA was rhythmic in WT, but not in clock mutant mice. Similarly, hedonic overconsumption was dependent on clock function. Together, this makes the VTA clock a promising candidate for the diurnal regulation of hedonically motivated food intake."*

• “…diurnal food choice regulation correlating with changes in DA signaling in the NAc” (p. 10). The data do not directly support this claim.

***Reply:*** *We have reworded this sentence. It now reads: "Together, these findings suggest a reward-related impact of clocks in the VTA on diurnal food choice regulation and time-of-day dependent changes in DA signaling in the NAc."*

• “Increased vulnerability to palatable food during the normal rest phase correlated with increased Th expression in the VTA and dopamine responses in the NAc” (p. 11). The data do not directly support this claim.

***Reply:*** *We have modified this sentence. It now reads: "Increased vulnerability to palatable food during the normal rest phase coincided with increased Th expression in the VTA and dopamine responses in the NAc."*

These statements should either be rephrased to accurately reflect the results, or the appropriate analyses should be included.

***Reply:*** *We thank the reviewer for this comment. For changes see above.*

Additional comments:

Fig. 4a should have 24 columns (4 mice/timepoint x 6 timepoints). I count 23. Please correct the missing column. As it is now, the lines denoting the timepoints do not correctly align with the columns.

***Reply:*** *We apologize for this mistake. The missing data set has been added.*

Please include additional methodological details pertaining to the microdialysis experiment. Specifically, how was dopamine induction calculated? Why is the effect of chocolate on dopamine levels (Fig. 2j) shown as dopamine induction and not dopamine concentration? Using the same metric for Figs. 2i and 2j would be helpful to permit comparisons between the two experiments. If two different metrics are to be shown, please reconsider referring to both as “dopamine levels” in the figure legend to avoid confusion. Was chocolate available during the entire sample period in 2j? Did chocolate consumption differ between the two epochs?

***Reply:*** *We have added this information to the respective methods and results sections and reworded the text in the figure legend. In brief, dopamine level changes (Fig. 2j) were calculated against pre-chocolate baseline levels for each individual. This normalization was necessary because of rather variable individual baseline dopamine concentrations in the dialysate (Fig. 2i) – in line with what was shown before (e.g. Ferris et al. Proc Natl Acad Sci U S A. 2014 Jul 1;111(26):E2751-9). Such normalization was not possible for the baseline levels because measurements were done on different animals. Chocolate was available during the whole sampling period. Animals ate about 75 % (0.45 g) of the snack without significant differences between time points.*

Reviewer #3 (Remarks to the Author):

NCOMMS-19-03435-Revised

Circadian regulation of hedonic appetite in mice by clocks in dopaminergic neurons of the ventral tegmentum

Koch CE, Kiehn JT, Griewahn L, Begemann K, Mauer J, Hess ME, Moser A, Schmid SM, Bruning JC, Oster H.

The authors appropriately addressed my initial concerns, I have only a few remaining remarks.

In the abstract the authors mention an increased circadian vulnerability to palatable foods during the “rest phase”, but at the end of the introduction they mention “early rest phase”. It seems “rest phase” is more appropriate since the early rest phase is specific for chocolate intake.

***Reply:*** *We thank the reviewer for noting this. We have changed the sentence accordingly.*

Lines 92-92: Any idea why the daily rhythm in chow intake was not more pronounced?

***Reply:*** *One would assume that inside the place preference system environment food intake is somewhat suppressed during the short 15-min test interval and this may mask any diurnal rhythms. Even for the snack condition, we tried several types of hedonic foods, but mice clearly have a different taste than humans. They like certain types of chocolate and cookies, though.*

Lines 111-113: This sentence starts with “Similar to the increased chow-induced place preference during the active phase”, but according to the information in line 92 no significant increase was observed.

***Reply:*** *The sentence in line 111-3 refers to the place preference (PI; Fig. 1d) which was indeed significantly higher during the active phase while line 92 reports chow intake itself, which was only slightly, but not significantly elevated.*

Line 129: “Dopamine” does not need a capital here.

***Reply:*** *We have changed this.*

Lines 131-134: The authors report a higher responsiveness of the NAc dopamine system during the rest phase. On the other hand, basal dopamine levels are higher during the active phase. The authors should explain why the higher basal levels do not results in a higher intake of palatable food during the active phase.

***Reply:*** *The reviewer is correct here. Our data suggest that it is the dopamine response rather than the baseline level that determines palatable food intake. However, experiments specifically manipulating dopamine levels in the NAc during snack conditions are required to address this directly. We have added a sentence on this to the discussion (p. 15).*

Lines 163-165: The final sentence of this paragraph can be removed as it is a repeat and not a conclusion based on the information in this paragraph.

***Reply:*** *We have removed this sentence.*

Lines 171-173: Why was this experiment not performed under circadian, ie DD, conditions?

***Reply:*** *The experiment aimed at identifying rhythmic regulation of VTA functions under standard experimental conditions, not so much identifying only those genes controlled by the circadian clock system. It can be expected that some of the rhythmic transcripts would not continue to oscillate in DD conditions. It would be interesting to further dissect VTA transcriptome regulation by comparing different light, food and clock gene deletion conditions (like the Menet group did for liver - Cell Rep. 2019 Apr 16;27(3):649-657), but this would be beyond the scope of this manuscript.*

Lines 196-197 and line 320: The authors mention that maximal TH expression at the beginning of the rest phase is phase-aligned with a higher hedonic intake. However, this only holds for chocolate intake, as sucrose/sucralose intake is higher during the second halve of the rest phase.

***Reply:*** *We modified this sentence to be more specific here.*

Lines 216-271: Here the authors report reduced VTA TH mRNA levels. Why would these lower TH levels only affect the diurnal rhythmicity of hedonic overconsumption and not overconsumption itself?

***Reply:*** *Total overconsumption is also reduced in the KD mice. We added a short statement in the results section.*

Lines 339-344, I think in their conclusion the authors should stress more clearly first, that the current results provide a nice explanation for why a HFD may result in more food intake during the light period and chronodisruption, and second that their findings implicate that the negative health effects of palatable foods are the result of two different mechanisms, not only does it result in hyperphagia and overconsumption, but it also results in feeding at-the-wrong time of day, i.e. chronodisruption. Here, the authors also might want to take into account the recent paper on chocolate for breakfast of Escobar et al., Sci Reports, 2020.

***Reply:*** *We thank the reviewer for this suggestion. We have modified the conclusion accordingly and also cite the Escobar paper.*

Line 350: Isn’t it more logical to assume such an increased preference at the end of the active phase instead of beginning of the rest phase?

***Reply:*** *Quite clearly, mice have to be awake to eat so this would imply that they are in their active phase when snacking. It was shown in mice (e.g. Kohsaka et al Cell Metab (*[*Cell Metab.*](https://www.ncbi.nlm.nih.gov/pubmed/?term=kohsaka+AND+bass+AND+high-fat) *2007 Nov;6(5):414-21) and suggested for humans (Gill et al* [*Cell Metab.*](https://www.ncbi.nlm.nih.gov/pubmed/?term=panda+AND+gill+ANd+smartphone) *2015 Nov 3;22(5):789-98) that food intake periods – and, thus active phases – might be much extended. We added a sentence on this to the discussion and also refer to the "normal" rest phase to make this distinction.*

**Reviewer comments, third version:**

Reviewer #3 (Remarks to the Author):

My concerns have appropriately been addressed. I have no further remarks.

**Author rebuttal, third version:**
